# Effect of temperature and glia in brain size enlargement and origin of allometric body-brain size scaling in vertebrates

**DOI:** 10.1186/s12862-014-0178-z

**Published:** 2014-10-03

**Authors:** Yuguo Yu, Jan Karbowski, Robert NS Sachdev, Jianfeng Feng

**Affiliations:** Centre for Computational Systems Biology, State Key Laboratory of Medical Neurobiology, Institutes of Brain Science, Department of Physiology and Biophysics, School of Life Sciences, Fudan University, Shanghai, 200433 People’s Republic of China; Department of Neurobiology, Yale school of Medicine, Charité Universitätsmedizin Berlin, 333 Cedar Street, New Haven CT 06510, Berlin, 10117 Germany; Institute of Applied Mathematics and Mechanics, University of Warsaw, Warsaw, 02-097 Poland; Institute of Biocybernetics and Biomedical Engineering, Polish Academy of Sciences, Warsaw, Poland

**Keywords:** Brain-body size, Allometric scaling, Glia, Body temperature, Brain enlargement, Metabolism

## Abstract

**Background:**

Brain signaling requires energy. The cost of maintaining and supporting energetically demanding neurons is the key constraint on brain size. The dramatic increase in brain size among mammals and birds cannot be understood without solving this conundrum: larger brains, with more neurons, consume more energy.

**Results:**

Here we examined the intrinsic relationships between metabolism, body-brain size ratios and neuronal densities of both endothermic and ectothermic animals. We formulated a general model to elucidate the key factors that correlate with brain enlargement, and the origin of allometric body-brain size scaling. This framework identified temperature as a critical factor in brain enlargement via temperature-regulated changes in metabolism. Our framework predicts that ectothermic animals living in tropical climates should have brain sizes that are several times larger than those of ectothermic animals living in cold climates. This prediction was confirmed by data from experiments in fish brains. Our framework also suggests that a rapid increase in the number of less energy-demanding glial cells may be another important factor contributing to the ten-fold increase in the brain sizes of endotherms compared with ectotherms.

**Conclusions:**

This study thus provides a quantitative theory that predicts the brain sizes of all the major types of animals and quantifies the contributions of temperature-dependent metabolism, body size and neuronal density.

**Electronic supplementary material:**

The online version of this article (doi:10.1186/s12862-014-0178-z) contains supplementary material, which is available to authorized users.

## Background

Brain size is a traditional metric of intelligence. Species with larger brain-body size ratios are often associated with greater intelligence, better tool-making skills, more social interactions, and other enhanced characteristics [1]. Although many correlations between large brains and complex behavioral measures have been posited over the past century, the driving forces and key factors underlying the development of larger brain-body size ratios in mammals and birds are still an open issue [2-13]. The evolution of large brains in mammals and birds coincided with the development of endothermy [14] at the end of the Triassic period (~200 million years ago) [15] during which time the first mammals evolved from therapsids (i.e., the mammal-like reptiles) and has continued in primates and humans, which have the largest brain-to-body weight ratios [16], to the present day [17]. In 1891, Snell [18] first described the allometric scaling relationship that emphasizes the dependence of brain size on body size. The most striking observation is that mammals and birds have developed larger brains that are approximately 5–50 times heavier than those of the lower-level vertebrates (e.g., reptiles, fish and amphibians) with similar body mass. It is surprising that mammals and birds developed larger brains, because, brain tissue is extremely demanding in terms of metabolic consumption, because the neuronal electronic signaling that occurs within the brain may have a per-neuron energy cost as high as ten times that of other cells in the body. This difference has attracted much attention to the search for links between brain size, body size and metabolic production. Martin [19] proposed that brain size is linked to maternal metabolic turnover via a 3/4 allometric scaling law [20]. This work indicated that the total amount of energy available from the body to supply the brain may constrain the size of the brain; however, species with larger brains do not have basal metabolic rates significantly higher than those in species with smaller brains [4,9,21-23]. There is also no significant difference in the percentage of energy supplied by the body to the brain between endotherms and ectotherms [24,25]. Given these similar metabolic processes and energy supply ratios, why do mammals and birds have larger brains than fish and reptiles of similar weight? How do mammals and birds meet the energy demands of brains that are 5–50 times larger? Although much research has focused on the several-fold differences in the sizes of the brains of primates and those of low-level mammals by considering the improved energy source acquisition styles of primates, such as improvements in food hunting style due to the use of tools, the consumption of cooked food, and differences in metabolic allocation, few studies have focused on the proximate causes of the differences in brain size between lower-level animals (e.g., fish, reptiles and amphibians) and higher-level animals (e.g., primates, mammals and birds). What is the role of temperature and metabolism in promoting or constraining brain size? What is the relative contribution of neuron and glial densities in increasing brain size?

In the present study, we re-examined the relationships between temperature, metabolism, neuronal density, energy cost of individual neurons, brain size and body size across of all the major types of vertebrates. Here we specifically focus on the critical effect of temperature and growth of glia cells in the development of large brain-body size ratio in mammals and birds and origin of allometric body-brain size scaling in vertebrates. We then develop a general model to describe the intrinsic relationship among the examined factors.

## Results

### Body temperature, metabolism, external temperature and brain size

**Figure 1:**
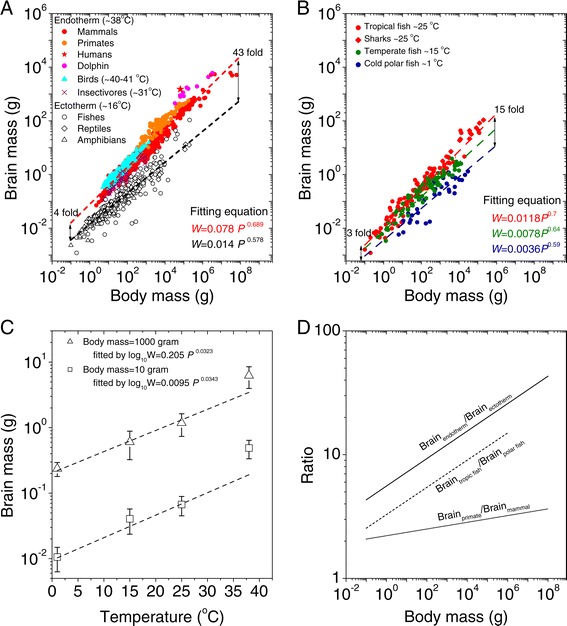
**Allometric relationships of the brain and body weights of vertebrate animals on a log-log plot. A**. Endothermic animals included 678 mammal species [3,16,22,61] (red open circle), 600 bird species [10] (cyan triangle) and 33 insectivore species [16] (purple cross). Ectothermic animals included 110 fish species [44] (black open circle), 71 reptilian species [11] (black open diamond), and 86 amphibian species [11] (black open triangle). The fitting functions are *W* = 0.078*P* ^0.717^ (adjusted- R^2^ = 0.95, p < 0.01) for endotherms and *W* = 0.014*P* ^0.578^ (adjusted- R^2^ = 0.91, p < 0.01) for ectotherms. All these datasets are provided in Additional file 1: Table S1. **B**. The relationship of the brains and bodies of randomly selected fish. Data from 30 polar-water fish species (blue, temperature ~1°C), 70 temperate species (green, 10–20°C), 88 tropical species (red, temperature 20–30°C) and 17 shark species that live in sub-tropical waters (red, core body temperature range 20–30°C [26,27]) were randomly selected from a fish database (http://fishbase.org) [44]. The fitting functions are *W* = 0.0036*P* ^0.59^ (adjusted- R^2^ = 0.82, p < 0.01) for polar-water fish, *W* = 0.0078*P* ^0.64^ (adjusted- R^2^ = 0.92, p < 0.01) for temperate-water fish, and *W* = 0.0118*P* ^0.7^ (adjusted- R^2^ = 0.94, p < 0.01) for tropical-water fish and the sharks. All these datasets are provided in Additional file 2: Table S2. **C**. Brain size as a function of temperature for fixed body masses, i.e., 10 grams and 1000 grams respectively. The open triangles represent the averaged data from 29 fish species with body masses of 5–15 grams, and the open squares represent the average of 33 species with body masses of 500–1500 grams from three temperature conditions. The data for 38°C are from 46 endotherms with body masses of 5–15 grams (solid square) and 52 endotherms with body masses of 500–1500 grams (solid triangle). The fitting equations are log_10_ *W* = 0.205*P* ^0.0323^ for *P* = 1000 g (adjusted- R^2^ = 0.98, p < 0.01 for only fish data) and log_10_ *W* = 0.0095*P* ^0.0343^ for 1000 g (adjusted- R^2^ = 0.91, p < 0.01, only for the fish data). **D**. The brain size ratios as a function of body sizes: Brain_endotherm_/Brain_ectotherm_ (black line), Brain_tropic fish_/Brain_polar fish_ (black dashed line) and Brain_primate_/Brain_mammal_ (black dotted line).

We first re-plotted the relationships between brain size and body size across different species (Figure 1A and Additional file 1: Table S1). In each type of species, the relationship between brain size and body size is highly ordered. Brain size increases with body size, and the relationship can be fit using the following allometric equation [16,18]:

1 $$ W=C{P}^{\alpha } $$

where exponent α is the allometric slope, C is a constant, and W and P are brain and body weight, respectively. Analyses have shown that C = 0.078 and α = 0.689 for endotherms (including mammals, birds, insectivores, primates, dolphins and humans), and C = 0.014 and α = 0.578 for ectotherms (primarily fish, reptiles and amphibians). The parameters C and α vary across species (see Additional file 1: Table S1). A closer examination of the distributions of body mass versus brain mass reveals that the warm-blooded, endothermic animals with relatively constant body temperatures of approximately 8°C (e.g., mammals, birds, primates and dolphins) form one distinct distribution, and cold-blooded ectothermic animals with body temperatures that vary with the environment and average approximately 16°C (i.e., fish, reptiles and amphibians) form a clear second distribution. Warm-blooded species have brains that are 4–43 times larger than those of ectothermic animals. The fact that the most apparent difference between the two groups is temperature suggests that it is a key factor that correlates with brain size.

If temperature is important, we might predict that cold-blooded species living in disparate environmental temperatures might also have different brain sizes. To examine whether the extrinsic, i.e., environmental, temperature also explains the variation in brain size, we used fish brain and body-size data from locations of different temperatures, obtained from a fish database (http://fishbase.org), and plotted the body-brain mass relationships. The fish and environments examined included the following: 1) 35 fish species living in polar conditions in which the average temperature was ~1°C, 2) 70 fish species living in temperate conditions with an average water temperature of ~15°C, 3) 88 species living in tropical conditions with an average water temperature of 25°C, and 4) 17 shark species living in sub-tropical waters that maintain their body temperatures at 20-30°C via steady contractile activity of muscles [26,27] (Additional file 2: Table S2; these data were randomly selected from the fish database). Surprisingly, we found that the fish brain-to-body mass ratio data fell into 3 distinct groups (Figure 1B). Fish living in warm tropical water had brains that were 2–5 times larger than those of the temperate-water fish and 3–15 times larger than those of the fish living in cold polar water. The fit equation for the fish species living in tropical conditions had an allometric slope of 0.7 and a constant of 0.0118 (red line and symbols), the equation for fish living in the temperate waters had an allometric slope of 0.64 and a constant of 0.0078 constant (green line and symbols), and the fish living in the polar waters had the shallowest allometric slope (0.59) and a constant of 0.0036.

Figure 1C directly quantifies the allometric relationship between the environmental temperature (from 1°C to 25°C) of the fish and their average brain mass for two body masses (10 g and 1,000 g). The fit equations indicate that fish brain mass more than doubles for each 10°C increase in temperature. As a comparison, the average mammalian brain sizes in animals weighing 10 g and 1,000 g are plotted in this figure (with a constant temperature of 38°C). This figure shows a nice fit between body mass and brain temperature for three points, but not for the largest brain sizes and highest temperatures. The data from the larger brain sizes/higher temperatures falls above the fit lines, indicating that temperature alone may not be the sole contributor to the development of larger brains. Other factors may contribute to the development of larger brains in mammals.

Together, these data indicate that temperature may be a critical factor in the marked changes in brain size during evolution in different climate conditions. Specifically, for a given body mass, warmer living conditions should result in larger brains.

In addition to this temperature-driven brain enlargement, the difference in brain sizes of endotherms and ectotherms increases as body size increases (Figure 1D). For small body sizes (below 1 gram), the brains of endotherms are atleast 4 times larger than those of ectotherms (black line in Figure 1D). Similarly, tropical-water fish have brains that are at least 3 times larger than those of cold-water fish (dashed line). In contrast to these effects in animals with small body sizes, for animals with body sizes over 100 kilograms, the brains of endotherms are approximately 20 to 40 times larger than those of ectotherms, and tropical-water fish have brains that are more than 10 to 15 times larger than those of cold-water fish. In comparison, the brains of primates are, relatively consistently, 2–3 times larger than those of other mammals, and this difference is less dependent on body size differences (dotted line in Figure 1D).

The emergence of intrinsically constant warm body temperatures together with an increasing brain size, with increasing body mass may be related to a temperature-dependent regulation of body metabolism. It is possible, that changes in body or environmental temperature affects the metabolic rate and consequently affected the evolution of large brains. This effect may depend on body mass.

**Figure 2:**
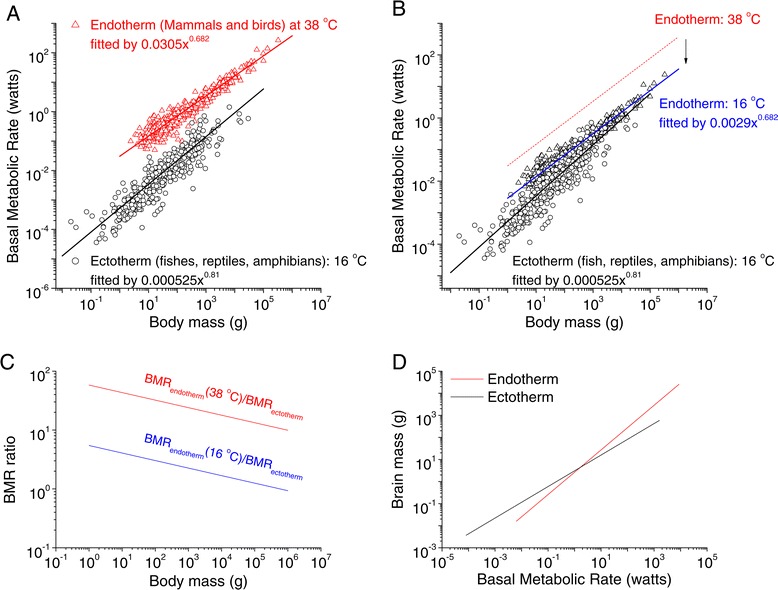
**Temperature-regulated basal metabolic rate (BMC) (watts) and brain size (gram). A**. Allometric relationships between basal metabolic rate (BMR) (watts) and body mass (gram) for the endotherms (including 525 mammals [43] and 320 birds [37,42]) (plotted in red triangles) and the ectotherms (including 265 fishes [37,42,44], 155 reptiles [37,42] and 158 amphibians [37,42]) (plotted in black open circles). The BMR versus body mass data were well fit by the equation 0.0305×^0.682^ (adjusted-R^2^ = 0.933, p < 0.01) for the endotherms and by 0.00525×^0.81^ (adjusted- R^2^ = 0.91, p < 0.01) for the ectotherms. The datasets are given in Additional file 3: Table S3. All the data plotted here for ectotherms were normalized to the temperature of 16°C, and the data for the endotherms were normalized to 38°C based on Eq. (2) (see also the methods in references [28,37]) to minimize the errors induced by measuring temperatures. **B**. When the metabolic rate data for the endotherms were normalized to 16°C, they merged into the group of ectothermic metabolic data. However, there some difference between these groups remained, particularly for the small animals. **C**. The BMR ratios as a function of body size for the endotherms to ectotherms was BMR_endotherm_(38°C)/BMR_ectotherm_ (16°C) (plotted in red line) and BMR_endotherm_(16°C)/BMR_ectotherm_ (16°C) (blue line). **D**. The brain masses (grams) as a function of body BMRs (watts) for the ectotherms (black line) and endotherms (red line) were based on the fit equations in Figures 1A and 2A.

The basal metabolic rate of the body (*M*_body_) has been found to be governed primarily by two interacting processes [28]: the Boltzmann-Arrhenius factor, which describes the temperature dependence of biochemical processes [29], and an allometric relation that describes how biological rate processes scale with body size for all vertebrates [20,28,30-42].

2 $$ {M}_{body}={C}_1{P}^{\gamma }{e}^{-\frac{E_v}{kT}} $$

where *P* is body weight, *C*_1_ is a constant (whose value is different for different species), *γ* is the allometric scaling factor, T is the absolute temperature, E_v_ is the activation energy (defined as the minimum amount of energy required to initiate a particular process. It is usually used in the context of chemical reactions, i.e., as the minimum amount of energy that chemical reactants must possess before they can undergo a chemical reaction. For the life metabolic process regulated by the temperature, *E*_*v*_ is expected to be around 0.65 electron Volts (eV) [28]), and k is Boltzmann’s constant (8.6×10^−5^ eV/K, here K is the temperature in Kelvin units). Earlier analyses have revealed that *C*_1_ = 0.0305 and *γ* = 0.682 for endotherms (these data, which are primarily from mammals and birds, were taken from references [42,43]), measured and scaled at 38°C, and *C*_1_ = 0.000525 and *γ* = 0.81 for ectotherms when normalized to a temperature of 16°C (see Figure 2A and Additional file 3: Table S3); these findings are consistent with those of recent reports [37,42,44]. When the metabolic rates of the endotherms are normalized to a common temperature of 16°C using Eq. (2), the metabolic rates of the endotherms (e.g., mammals and birds) remain several times higher than those of ectotherms (e.g., fish, amphibians and reptiles), particularly for animals with small body sizes (Figure 2B). In large animals, the metabolic rates of the endotherms and ectotherms were similar (Figure 2B and C). These findings suggest that at similar metabolic rates, large endotherms should support brain masses several times greater than those of ectotherms (Figure 2D), given that brain size differences increase with body size among endotherms and ectotherms (Figure 1D).

These analyses suggest that temperature-dependent body metabolism alone cannot explain the differences in brain size among endotherms than among ectotherms. These results also indicate that other factors may play a role in explaining the differences in brain size between warm blooded endothermic animals and cold blooded exothermic animals. It is possible that neural, glial density or the energy demands on these cells are dependent on body temperature.

### A general model linking brain and body sizes to metabolism and temperature

**Figure 3:**
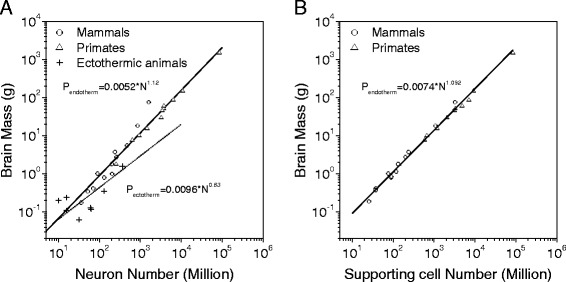
**Scaling relationship between the total number of neurons or supporting cells in the brain and brain mass. A** Brain mass (in gram) compared with the total number of neurons in the brain for 12 low-level mammal species [47,62] (solid circle), 11 primates [46,63] (solid triangle) and 7 ectotherms [44,48-50]. The fit function (in black line) gives *W* = 0.0052 *N* ^1.12^ (adjusted- R^2^ = 0.94, p < 0.01) for the endotherms (where P_endotherm_ is the brain mass in grams, *N* is total number of neurons in the brain in millions, and P_ectotherm_ = 0.0096 *N* ^0.82^ (adjusted- R^2^ = 0.65, p < 0.05) for the endotherms. **B** Brain mass in grams compared with the total number of non-neuron cells in the brain for 12 low-level mammal species [47,62] (solid circle), 11 primates [46,63] (solid triangle) and 7 ectotherms [44,48-50]. The fit functions (in black lines) have P_endotherm_ = 0.0074 *N* ^1.092^ (adjusted- R^2^ = 0.993, p < 0.01) for the endotherms (where P_endotherm_ is brain mass in grams, and *N* is the total number of non-neuron cells in millions. The datasets for this figure are given in Additional file 4: Table S4.

In this section, we derive a general model to quantify the relationships between brain size, temperature-dependent metabolism, individual neuronal metabolism and body size to account for the origin of Eq. (1). We began by considering the relationship between brain mass and the number of neurons and supporting cells in the brain. Brain mass (*W*) is determined by the total number of neurons and supporting cells; hence *W = Nw+ N*_*1*_*w*_*1*_, where *N* and *w* are the number and weight of brain neurons, respectively, and *N*_1_ and *w*_1_ are the corresponding quantities for the supporting cells. If we assume that the weight and number of the supporting cells are proportional to those of the neurons, i.e., if *N*_*1*_*= k*_*1*_*N*, and *w*_*1*_*= k*_*2*_*w*, then the relationship between brain mass and neurons simplifies to *W = C*_*2*_*N*, where *C*_*2*_* = (1+k*_*1*_*k*_*2*_*)w*. This theoretical picture in which *W* is proportional to *N* is supported by a recent analysis of mammalian and primate brains (see also Figure 3A and Additional file 4: Table S4). Analyses of these data yielded.

3 $$ W={C}_2{N}^{\beta } $$

where β is close to 1 (here C_2_ = 0.0052 and β = 1.12 for both rodents and primates) as reported in recent studies [23,45-47, 63]. The numbers of supporting non-neuronal cells in endotherms are proportional to the numbers of neurons and are also strongly correlated with brain mass (Figure 3B). The supporting cells play an important role in providing mechanical and trophic support to neurons. Additionally, data from several ectothermic animals [44,48-50] indicate that that C_2_ = 0.0096 and β = 0.83 (see Additional file 4: Table S4; the numbers of non-neuronal cells are not available for ectotherms).

**Figure 4:**
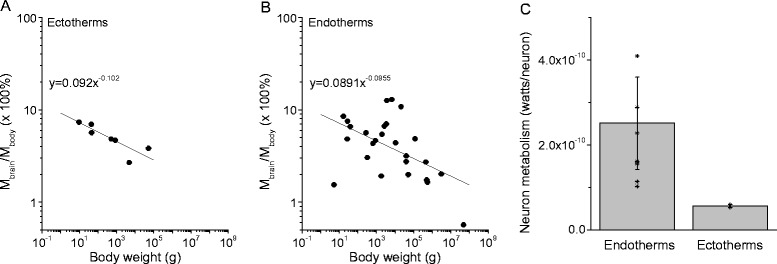
**The dependence of brain metabolism on body size and invertebrate species.** **A)** The ratios of brain metabolism to resting body metabolism as a function of body mass (grams) for 7 ectotherms [25] were fit by y = 0.092×^-0.102^ (adjusted-R^2^ = 0.63, p < 0.05), and for 26 endotherms [25] which were fit by y = 0.0891×^-0.0955^ (adjusted- R^2^ = 0.231, p < 0.05), **B)**. **C**. The average metabolic cost of an individual neuron for endotherms was 2.51 ±1.09 × 10^−10^ watts, and for ectotherms this cost was 0.563 ±0.03 × 10^−10^ watts [25]. The datasets used for this figure are given in Additional file 5: Table S5.

Neurons are the brain’s major energy consumer, and most (~80%) of the brain’s energy is consumed by signaling processes within neurons [51,52]. Brains are energetically costly. Experimental investigations have shown that the brain’s metabolic consumption accounts for 1-10% of the total body metabolic rate (*M*_body_) in most animal species, although brain mass accounts for no more than 1% of an animal’s body weight [24,25]. Hence, the total energy required to support the brain can be quantified as

4 $$ {M}_{brain}=\varphi {M}_{body} $$

where *M*_brain_ is the brain’s basal metabolic rate, *M*_body_ is the body’s basal metabolic rate as defined in Eq. (2), and *φ* is a variable that varies with species. Re-examination of the data reported by Mink et al. (1981) suggested that *φ* can be described by the following equation for both ectotherms and endotherms

5 $$ \varphi ={C}_3{P}^{\zeta } $$

where *P* is the body mass, C_3_ = 0.092 and *ζ* = −0.102 for ectotherms, and C_3_ = 0.081 and *ζ* = −0.092 for endotherms (see Figure 4A and B and Additional file 5: Table S5 [25]).

The total amount of energy supplied to the brain (*M*_brain_) should be equal to the total energy consumption of the brain’s neurons. Note that here we make a simplifying assumption, by considering the metabolic costs of neurons only because neurons consume most of the energy while the supporting cells account for 10-20% of total brain energy. If the total number of neurons in the brain is *N* and the metabolic consumption of each individual neuron is *M*_neuron_, we can describe the relationship as follows:

6 $$ N=\frac{M_{brain}}{M_{neuron}}=\frac{\phi {M}_{body}}{M_{neuron}} $$

where the total number of neurons in the brain (*N*) contributes directly to the brain’s mass (see Eq. 2). By inserting Eqs. (2)-(5) into Eq. (6), we obtained a general model of the relationships between brain mass (*W*), body mass (*P*) and temperature (*T*):

7 $$ W=\lambda {P}^{\alpha }{e}^{\frac{-{T}_v}{T}} $$

where $\lambda ={C}_2{\left(\frac{C_1{C}_3}{M_{neuron}}\right)}^{\beta }$, *α* = *βγ* + *βξ*, and ${T}_v=\frac{\beta {E}_v}{k}$, called effective temperature. Equation (7) links body temperature, body mass, brain mass and neuron metabolic rate (*M*_neuron_), provides a general theoretical framework for quantifying changes in brain size during evolution, and provides an origin of the formula of Eq. (1) that was derived from experimental data. The temperature range used for Eq. (7) is 0 ~ 42°C, corresponds to what is normally observed in polar fish to the body temperature of birds. The equations are not valid for temperatures above or below these values, because all the scaling parameters have been derived from animal data acquired within this temperature range.

Equation (7) links brain mass with the metabolic consumption rate of individual neurons (*M*_neuron_). Recent studies suggest that the energy cost per neuron (*M*_neuron_) in the brain is stable and nearly invariant across mammal species [23]. Calculations based on the available data revealed that *M*_neuron_ is, on average, 2.51 × 10^−10^ watts for endotherms and 0.563 × 10^−10^ watts for ectotherms (see Figure 4C and Additional file 5: Table S5).

**Figure 5:**
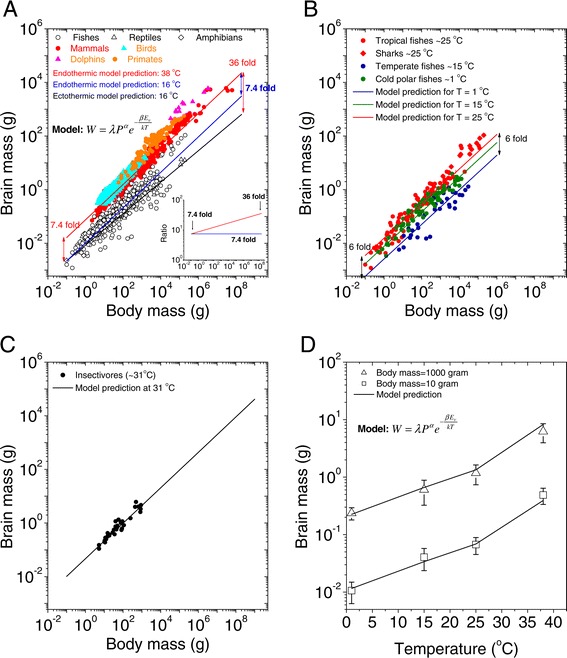
**Model prediction for allometric scaling between the brain and body weights of vertebrate animals on a log-log plot. A**. For the brain-body mass data listed in Figure 1A, the model predictions based on Eq. (7) for endotherms in 38°C (in red line) and for ectotherms in 16°C (in black line) are displayed. Multiple linear regression analysis (here body mass P is the regressor variable while temperature T is fixed as a constant) for the endothermic prediction yielded adjusted-R^2^ = 0.958, p < 10^−5^, and for ectothermic prediction, these results were adjusted- R^2^ = 0.933, p < 10^−5^. Insert: ratios of predicted endothermic brain sizes to ectothermic brain sizes as a function of body mass (red line). For comparison, the ratios of predicted endothermic brain sizes at 38°C to the predicted ectothermic brain sizes at 16°C using all the endothermic parameters are presented (blue line). **B**. For the fish brain-body mass data listed in Figure 1B, the model predictions based on Eq. (7) for fish in 1°C (in blue line), 15°C (in green line) and 25°C (in red line) conditions are shown. Least-squares regression analysis of the model predictions and the actual data revealed that, for the tropical-water fish, *N* = 105, CC = 0.981, adjusted- R^2^ = 0.962, p < 10^−5^, for the temperate-water fish *N* = 70, CC = 0.961, adjusted- R^2^ = 0.923, p < 10^−5^, and for the polar-water fish *N* = 35, CC = 0.889, adjusted- R^2^ = 0.79, p < 10^−5^. **C**. For the brain-body mass data of the insectivores listed in Figure 1A, the model predictions based on Eq. (7) and the endothermic parameters at the temperature of 31°C (in black line) produced regression analysis results of CC = 0.952, adjusted- R^2^ = 0.91, p < 1×10^−5^. **D**. For the fish data in Figure 1C, the model predictions for the relationship between brain size and temperature for the two body sizes of 10 grams and 1000 grams based on Eq. (7) are given in black lines. Multiple linear regression analysis was used here (temperature T is the regressor variable) between log_10_(actual brain mass) and log_10_(model prediction).

We inserted empirically determined parameters (see the analyses in Figures 3 and 4) into Eq. (7) to determine how well this data-based theoretical model predicts actual animal brain size. Figure 5A plots the results of linerar regression analysis between log_10_ (brain mass) and log_10_ (brain mass prediction) for endotherms (red line) -- mammals, birds, primates and dolphins, primarily -- at 38°C and endothermic animals at 16°C (black line). The predicted parameters: for endotherms were *λ* = 0.0749 and α = 0.6608, and the analysis results were a correlation coefficient CC = 0.979, an adjusted-R^2^ = 0.958, and a mean squared error MSE = 0.042, p-value p <1×10^−5^). For endothermic animals at 16°C, the predicted parameters were: *λ* = 0.0085 and α = 0.5876) and the multiple linear regression analysis results were CC = 0.966, adjusted-R^2^ = 0.933, and a MSE = 0.0552 (p-value <1×10^−5^). In this analysis body mass *P* was the regressor variable and temperature was a constant. The model predictions closely matched the real brain data, with data-derived parameters of *C*_1_ = 0.0305, *γ* = 0.682, *C*_2_ = 0.0052, *β* = 1.12, *C*_3_ = 0.081, and *ζ* = −0.092 for the endotherm-based data analysis that resulted in the equation $W=0.0749{P}^{0.6608}{e}^{\left(\frac{1.12{E}_v}{k}\left(\frac{1}{T+273.15}-\frac{1}{38+273.15}\right)\right)}$ (where *P* represents body mass in gram) based on Eq. (7) and *C*_1_ = 0.000525, *γ* = 0.81, *C*_2_ = 0.0096, *β* = 0.83, *C*_3_ = 0.092 and *ζ* = −0.102 for ectotherms, which led to the following equation: $W=0.0087{P}^{0.5876}{e}^{\left(\frac{0.83{E}_v}{k}\left(\frac{1}{T+273.15}-\frac{1}{16+273.15}\right)\right)}$. The difference in brain size of endotherms compared to ectotherm (i.e., Brain_endotherm_/Brain_ectotherm_) also increased from 7.4 to 36-fold with increasing body masses (inset of Figure 5A). If we constrain all parameters in ectotherms, in the same fashion as we have done for endotherms with the exception of the temperature difference, the brain size prediction for the ectotherms is only 7.4-fold lower than that for the endotherms (blue line in Figure 5A and, the inset). Furthermore the scaling parameter of α = 0.6608 from Eq. (7) is close to the measured value of 0.689 for endotherms, and α = 0.5876 is close to the measured value of 0.578 for ectotherms (Figure 1). Thus, temperature-regulated metabolism can only partially explain the 4-40-fold brain size difference shown in Figure 1A, and other factors, such as increases in non-neuron cell (mostly glial) numbers (as reflected in Figure 4B), may be essential factors in explaining brain size differences. Thus, contributions from metabolism, neural density and temperature together explain most of the differences in brain mass between endotherms and ectotherms shown in Figure 1A.

Note that model predictions for endothermic brain sizes are generally 2–5 times lower than actual brain masses of primates and dolphins, suggesting there other evolutionary factors mat contribute to the larger brain size in these species, as has been indicated in recent reports [12,13,53]. The model in the present study does not make any attempt to capture these effects.

The model predictions for tropical-water fish, temperate-water fish, and cold-water fish are shown in Figure 5B, which predicts that brain size at 25°C should be 6-fold higher than that in fish at 1°C and that at 15°C brain size should be 3-fold higher than at 1°C. The model used actual data-derived parameters of C_1_ = 0.00051, *γ* = 0.879, C_2_ = 0.0096, β = 0.83, C_3_ = 0.092 and *ζ* = −0.102, which for fish results in the equation $W=0.0085{P}^{0.645}{e}^{\left(\frac{0.83{E}_v}{k}\left(\frac{1}{T+273.15}-\frac{1}{16+273.15}\right)\right)}$ based on Eq. (7). Note, data for fish brain, glial density data are currently not available, so the fitting is imperfect, and only includes the temperature- related differences.

The model prediction for insectivores (with body temperatures of approximately 31°C) is perfectly aligned with the actual data with predicted parameters of *λ* = 0.0409 and α = 0.6608 (CC = 0.952, adjusted- R^2^ = 0.91, MSE = 0.0211, p < 1×10^−5^). Figure 5D quantifies the allometric relationship between the temperatures that fish live at, and their average brain mass for two body masses (10 g and 1,000 g). The equations for the fish were given by $W=0.0085{P}^{0.645}{e}^{\left(\frac{0.83{E}_v}{k}\left(\frac{1}{T+273.15}-\frac{1}{16+273.15}\right)\right)}$ and for mammals were given by $W=0.0749{P}^{0.6608}{e}^{\left(\frac{1.12{E}_v}{k}\left(\frac{1}{T+273.15}-\frac{1}{38+273.15}\right)\right)}$. At a given body weight, as temperature increases, brain mass of fish increases, but the increase of brain mass with temperature is larger in mammals. This relationship is well captured by the model predictions (see the black line in Figure 5D), with multiple linear regression analysis -- here temperature T is the regressor variable -- between log_10_ (actual brain mass) and log_10_(model prediction) for 10 g: CC = 0.998, adjusted- R^2^ = 0.995, MSE = 0.0041, p < 0.0023; and for 1000 g: CC = 0.9999, adjusted- R^2^ = 0.9998, MSE = 0.0049, p < 0.0001.

**Figure 6:**
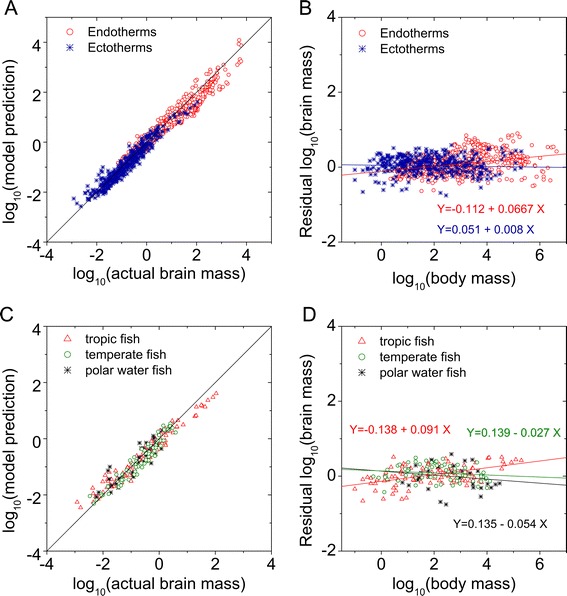
**Regression residual analysis of model prediction and actual brain mass. A**. Least-squares regression of the log_10_(model prediction) versus log_10_(actual brain mass) of the endotherms (including mammals, birds, primates and dolphins) (*N* = 1335), and ectotherms (including fishes, reptiles and amphibians) (*N* = 367). **B**. Least-squares regression of the residuals of the log_10_(brain mass) = log_10_(actual brain mass)- log_10_(model prediction) versus the log_10_(body mass) for the endotherms (red triangles) (*N* = 1335, CC = 0.411, adjusted- R^2^ = 0.169, p < 0.01) and the ectotherms (blue plus) (*N* = 367, CC = 0.0488, adjusted- R^2^ = 0.0024, p < 0.35). **C**. Least-squares regression of the log_10_(brain mass) versus log_10_(body mass) for the tropical-water fish (*N* = 105, adjusted- R^2^ = 0.962, p < 10^−5^), temperate-water fish (*N* = 70, adjusted- R^2^ = 0.924, p < 10^−10^), and polar-water fish (*N* = 35, adjusted- R^2^ = 0.79, p < 10^−5^). **D**. Least-squares regression of the residuals of the log_10_(brain mass) versus log_10_(body mass) for the tropical-water fish (*N* = 105, adjusted- R^2^ = 0.278, p < 10^−5^), temperate-water fish (*N* = 70, adjusted- R^2^ = 0.0227, p < 10^−5^), and polar-water fish (*N* = 35, adjusted- R^2^ = 0.0302, p < 10^−5^).

By applying the general model described by Eq. (7) to all the major types of vertebrates (including endotherms: primates, dolphins, mammals and birds, and ectotherms: fishes, reptiles and amphibians), we found that the actual brain masses and model predictions were aligned well in a log-log plot (Figure 6A). The multiple linear regression results were CC = 0.979, adjusted- R^2^ = 0.958, MSE = 0.0421, p < 10^−5^ for endotherms and CC = 0.966, adjusted- R^2^ = 0.933, MSE = 0.0522, p < 10^−5^ for ectotherms. Figure 6B shows the residuals of the log brain sizes i.e., log_10_(actual brain mass)-log_10_(model prediction), for both the endotherms and ectotherms as a function of log body size with slopes close to 0, indicating that the model performed well in predicting actual brain mass. Figure 6C shows that actual brain masses of fishes and model predictions were also aligned in a log-log plot (CC = 0.966, adjusted- R^2^ = 0.932, MSE = 0.0674; p < 1×10^−5^). The model predictions were underestimates compared to the actual brain masses for the tropical fish with larger body sizes, and were overestimates compared to the actual brain masses for smaller tropical fish. The model underestimated the actual brain masses for fish from cold – i.e. polar -- water (Figure 6C), but for the temperate-water fish the model matched the real data well (Figure 6C). Note that the model is missing parameters, it is worth noting that the model relies on actual data, and that for both tropical-water and polar-water fish there is a dearth of data for neural and supporting cell densities. With adequate samples of all important measures, i.e. the kinds of data we have for mammals and primates, shown in Figure 3, the model predictions would be improved. Figure 6D shows the residuals of the log brain sizes, i.e., log_10_(actual brain mass)-log_10_(model prediction), for three groups of fishes as a function of log body sizes. This has a flat relationship with slopes close to 0, indicating that the model performed well in predicting actual fish brain mass.

**Figure 7:**
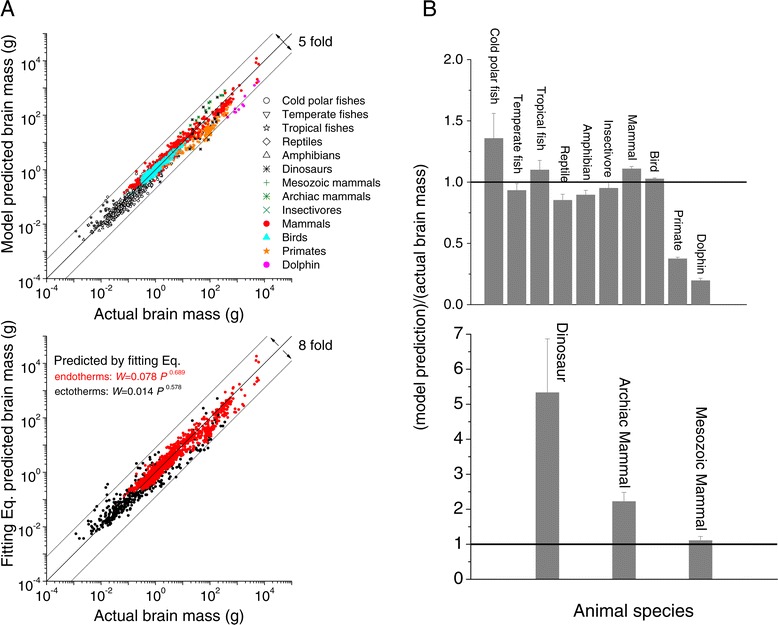
**Matching analysis between model predictions and actual brain mass for all the major type of vertebrates. A**. The model (based on Eq. (7)) predicted brain mass versus real brain mass for all the major types of vertebrates. The correlation coefficient CC and mean squared error MSE between model prediction and real brain mass are listed here: CC = 0.89 and MSE = 0.1 for cold polar fishes; CC = 0.961 and MSE = 0.05 for temperate fishes; CC = 0.981 and MSE = 0.068 for tropical fishes; CC = 0.958 and MSE = 0.052 for reptiles; CC = 0.943 and MSE = 0.028 for amphibians; CC = 0.803 and MSE = 0.51 for dinosaurs; CC = 0.97 and MSE = 0.0162 for mesozoic mammals; CC = 0.965 and MSE = 0.089 for archaic mammals; CC = 0.952 and MSE = 0.021 for insectivores; CC = 0.987 and MSE = 0.029 for mammals; CC = 0.966 and MSE = 0.007 for birds; CC = 0.956 and MSE = 0.23 for primates; CC = 0.97 and MSE = 0.544 for dolphins. **B**. The ratios defined as (Model predicted brain mass)/(Actual brain mass) for the 13 animal species.

Finally, we used Eq. (7) to predict brain sizes of all the types of vertebrates (also including 7 mesozoic and 18 archiac mammals (with body temperature around 31°C) and 17 dinosaurs (with temperature 16°C)). Top panel in Figure 7A shows that actual brain masses, and the model predictions tracked each other well in a log-log plot (multiple linear regression analysis results: CC = 0.978, adjusted- R^2^= 0.956; MSE = 0.0495; p < 1 × 10^−5^). Compared to the ten- to hundred-fold variance in brain size for the same body mass shown in Figure 1A, the model predictions and actual brain sizes exhibited a rather small variance, and only ~ 5-fold difference (Figure 7A). The predicted brain mass from the fitting equations in Figure 1, and the actual brain mass also tracked each other well, but the variance was a little bit higher, roughly 8 fold than in the analysis used in top panel. The multiple linear regression analysis of these data show that: CC = 0.973, adjusted- R^2^ = 0.946; MSE = 0.0544; p < 1 × 10^−5^. Note that the adjusted- R^2^ = 0.946 is slightly less than the value 0.956 in top panel using Eq. (7). This suggests that Eq. (7) fits the data better than do the fitting equations from Figure 1A because that both body mass and temperature play important roles in determining the brain mass.

For most of animal species (i.e., temperate and tropic fishes, reptiles, amphibians, insectivores, mammals and birds), model predictions matched real brain data well, with CC > 0.95, MSE = 0.07, p < 10^−5^) with ratios of (model prediction)/(actual brain mass) close to 1 (Figure 7B) while there are larger mean square errors for primates (MSE = 0.23, CC = 0.956) and dolphins (MSE = 0.544, CC = 0.97), and dinosaurs (MSE = 0.51, CC = 0.803) with ratios of (model prediction)/(actual brain mass) either much less or much more than 1 (Figure 7B). The regression analysis gives CC = 0.965, adjusted- R^2^ = 0.932, MSE = 0.089, p < 1 × 10^−5^ for archiac mammals, and CC = 0.97, adjusted- R^2^ = 0.94, MSE = 0.0162, p < 1 × 10^−3^ for mesozoic mammals.

The predictions of the model could be improved by more accurate measurements of the body temperature, body metabolism, neuronal metabolism and neuronal/glial densities of each group of species.

## Discussion and conclusion

There is increased interest in examining the key factors facilitating enlargement of the brain during evolution. Given that the energy cost of the brain’s neurons is more than ten times that of the cells of the body, the factors that regulate the brain’s energy supply and energy cost should be given thorough consideration. In this study, we re-examined data on brain size, body size and metabolic rates for all the major types of vertebrates, developed a theoretical framework based on examination of the real data, and identified several critical factors that may trigger the dramatic enlargement of the brains of birds and mammals as compared with fish, reptiles and amphibians. The most critical factor was temperature. When the endothermic animals evolved from ectothermic animals, two unique features were bound together in endotherms: larger brains and higher body temperatures (on average, 20°C higher than those of ectothermic animals). The warm temperature was critical in speeding the biochemical processes and making marked metabolic differences. Our analysis revealed that in small animals the basal metabolic rate of endotherms is 60 times greater than that of ectotherms, and in large animals it is ~ 10 times greate (Figure 2C). However, the brain sizes of small endotherms are only approximately 4 times greater than that of small ectotherms but the brains of large endotherms are more than 40 times greater than the brains of large ectotherms. Clearly, in large animals, the same basal metabolic rate of endothermic animals should support larger brain tissue in ectothermic animals, than it actually does (Figure 2D), suggesting that temperature was only one of the key factors in mediating the enlargement of the brains of endotherms. Note a similar analysis published recently points out that temperature correlates with brain size [54].

Other factors included in our analysis are brain metabolism and number of supporting glial neurons. Analysis of the brain metabolism data showed that that the average metabolic cost of individual neurons in ectothermic brains is ~ 5.63 × 10^−11^ watts, which is nearly 5 times lower than the average metabolic cost of endothermic brains (Figure 4C). The lower energy cost of individual neurons could be attributed to the effect of colder temperature on metabolism and the occurrence of fewer synapses in ectothermic brains [55], or the lower energy cost of individual neurons could be associated to the smaller number of glia in ectotherms [46,47,56]. Glia are the major non-neuronal supporting cells that maintain homeostasis, form myelin, and provide mechanical and tropic support to neurons. In the vertebrate CNS, most glia originate from portions of the developing neural tube—the exception being olfactory ensheathing glia [57]—and the energy cost of glia is generally only a few percent of that of neurons [56,58]. Recent data suggest that the evolution of the nervous system was accompanied by increases in the number and size of glia [46,47,56]. In vertebrates, the glia-to-neuron ratio in the cortex increases with the size of the brain [56,58]. Figure 3 and Eq. (3) show that brain mass increases much faster in endothermic brains than in ectothermic brains as the number of neurons increase, which is also suggestive of increases in the numbers of non-neuronal supporting cells. Glia contribute to the faster propagation of nerve signals and long-range communications, particularly via the evolution of myelinating glia in developed endotherms [59]. These energetically inexpensive glial cells surround neurons and occupy a relatively large portion of the brain [60], while also providing energy support to neurons and aiding efficient communication between cortical areas [59,60]. Thus, these cells may be one of the critical factors that promoted brain enlargement.

The evolution of the large brains of endotherms is associated with multiple factors. To quantify the contribution of each factor to the brains of mammals and birds that are tens of times larger than those of fish, reptiles and amphibians, we built up a general theoretical model to examine the origin of body-brain size allometric scaling relationship by linking the key factors of temperature-dependent metabolism, neuron energetics, neuronal density and body size. This basic framework suggests that temperature and the development of supporting non-neuronal cells may have been the critical factors that correlated with, or might even be causal, for the enlargement of the brains of endotherms (Figure 5A). This framework also predicts that the brains of cold-blooded animals living in warmer conditions should be several times larger than those of cold-blooded animals that live in colder conditions. This prediction was confirmed by the analysis of the brain sizes of fish living in various temperatures (Figures 1B and 5B). Indeed, owing to the absence of measurements of neuronal density (and glia-to-neuron ratios) for the brains of fish that live in different temperatures, the model prediction in Figure 5B did not capture the differences in the slopes between the tropical and polar fish that are shown in Figure 1B and D. However, a prediction from the endothermic brain would be that tropical fishes have larger numbers of glia in their brains than do the fish that live in cold conditions. These predictions can be tested by experimental investigations.

## Methods

### Ethics

No new animal/human data was generated in this study. All the data used in this study are from references and online database.

### Animal data sources

The brain and body size datasets for the endothermic and ectothermic animals (Figure 1A) are from references [3,16,22,61] and are listed in Additional file 1: Table S1. The body and brain size datasets for fish that live in different temperatures and were used to create Figure 1B were collected from the database at www.fishbase.org[44] and are listed in Additional file 2: Table S2. The metabolism-to-body scaling relationships of the endothermic and ectothermic animals were collected from published papers [37,42-44] that are listed in Additional file 3: Table S3 and are plotted in Figure 2. The data regarding the allometric scaling of the brain mass and total neuronal numbers for the endothermic and ectothermic animals were collected from published papers [47,62] and are listed in Additional file 4: Table S4 and plotted in Figure 3. The datasets for the ratios of brain metabolism to resting body metabolism are listed in Additional file 5: Table S5, are plotted in Figure 4 and were collected from published papers [25].

## Additional files

Additional file 1:
**Animal datasets for the allometric scaling relation of Brain-to-Body mass for endothermic and ectothermic animals.** Data are from personal communication with Harry J. Jerison and his previous publication [16], and collected from reference [3,16,22,61]. Endothermic animals include 678 mammal species (including primates and human brain) **(Table S1a)**, 600 bird species **(Table S1b)**, 33 insectivore species **(Table S1c)**, 18 archaic mammalian species **(Table S1d)**, 7 mesozoic mammals **(Table S1e)**, and 17 dinosaur species **(Table S1f)**. The samples for ectothermic animals include 110 fish species **(Table S1g)**, 71 reptilian species **(Table S1h)**, and 87 amphibian species **(Table S1i)**. Additional file 2:
**Animal datasets for the allometric scaling relation of fish Brain-to-Body mass in different water conditions.** Database www.fishbase.org was searched for fish brain-body mass data, of which 35 species living in polar ocean (0–5°C), 70 species living in temperate ocean (10 ~ 20°C), 88 species living in tropical water condition (20 ~ 30°C), and 17 shark species in sub-tropical water condition (15 ~ 25°C) are randomly choosed. These data are listed in **Table S2a**, **S2b**, **S2c**, and **S2d**. Additional file 3:
**Animal datasets for the allometric scaling relation of metabolism-to-body for endothermic and ectothermic animals.** Basal metabolic rates for amphibians **(Table S3a)** and reptiles **(Table S3b)** were modified from references [37,42]; for fishes **(Table S3c)** from references [37,42,44]; for birds **(Table S3d)** [37,42]; and for mamals [43] (which is Additional file 1: Table S1 in this reference, and data is not listed here). Additional file 4:
**Animal datasets for the allometric scaling relationship between brain mass and total neuronal number for endothermic and ectothermic animals.** Data from 12 mammals **(Table S4a)**, 11 primates **(Table S4b)** and 7 ectotherms **(Table S4c)**. Additional file 5:
**Animal datasets for the ratios of brain metabolism to resting body metabolism.** Data from 26 endotherms **(Table S5a)** and 7 ectotherms **(Table S5b)**.
