# Supplementary material for: Effect of temperature and glia in brain size enlargement and origin of allometric body-brain size scaling in vertebrates
Source: BMC Evol Biol. 2014 Oct 3;14:178. doi: 10.1186/s12862-014-0178-z (PMC4193995; doi:10.1186/s12862-014-0178-z)
Supplement: Additional file 1: — Animal datasets for the allometric scaling relation of Brain-to-Body mass for endothermic and ectothermic animals. Data are from personal communication with Harry J. Jerison and his previous publication [16], and collected from reference [3,16,22,61]. Endothermic animals include 678 mammal species (including primates and human brain) (Table S1a), 600 bird species (Table S1b), 33 insectivore species (Table S1c), 18 archaic mammalian species (Table S1d), 7 mesozoic mammals (Table S1e), and 17 dinosaur species (Table S1f). The samples for ectothermic animals include 110 fish species (Table S1g), 71 reptilian species (Table S1h), and 87 amphibian species (Table S1i). [file 12862_2014_178_MOESM1_ESM.docx]

**Supplement Table S1 Dataset:**

**Allometric scaling relation of Brain-to-Body mass for endothermic and ectothermic animals**

Data are from personal communication with Harry J. Jerison and his previous publication[^1^](#_ENREF_1), and collected from reference[^1-4^](#_ENREF_1). Endothermic animals include 678 mammal species (including primates and human brain) **(Table S1a)**, 600 bird species **(Table S1b)**, 33 insectivore species **(Table S1c)**, 18 archaic mammalian species **(Table S1d)**, 7 mesozoic mammals **(Table S1e)**, and 17 dinosaur species **(Table S1f)**. The samples for ectothermic animals include 110 fish species **(Table S1g)**, 71 reptilian species **(Table S1h)**, and 87 amphibian species **(Table S1i)**.

**Table S1a: 678 mammal species**[^1-4^](#_ENREF_1).

| **Order** | **Species** | **Body mass (gram)** | **Brain mass (gram)** |
| --- | --- | --- | --- |
| **Delphin** | *Phocoena* | 142430 | 1735 |
|  | *Lagenorhynchus* | 89750 | 1136.75 |
|  | *Stenella* | 63500 | 883.75 |
|  | *Tursiops* | 170480 | 1572.99683 |
|  | *Delphinus* | 65086.95652 | 797.2609 |
|  | *Stenella* | 33600 | 450 |
|  | *Phocoenoides* | 98333.33333 | 833.66667 |
|  | *Orcinus* | 3.273E6 | 6052 |
|  | *Delphinapterus* | 498250 | 1921 |
|  | *Pseudorca* | 2E6 | 4307 |
| **Primates** | *Saguinus tamarin* | 410 | 9.5 |
|  | *S. midas* | 350 | 10.4 |
|  | *S.Oedipus* | 413 | 9.8 |
|  | *Callicebus moloch* | 670 | 17.6 |
|  | *C.cupreus* | 514 | 14 |
|  | *Alouatta villosa* | 7824 | 65.5 |
|  | *A.seniculus* | 3560 | 46.8 |
|  | *Cebus capucinus* | 3765 | 73.8 |
|  | *C.albifrons* | 1640 | 80 |
|  | *C.apella* | 2400 | 75 |
|  | *Saimiri sciureus* | 630 | 24.8 |
|  | *S.oerstedii* | 893 | 26.4 |
|  | *Ateles paniscus* | 7400 | 106.4 |
|  | *A.belzebuth* | 8890 | 118.4 |
|  | *A.geoffroyi* | 7787 | 117 |
|  | *Cercopithecus aethiops* | 4819 | 73.2 |
|  | *C.pygerythrus* | 5670 | 72.6 |
|  | *C.lhoesti* | 8500 | 93 |
|  | *C.hamlyni* | 6000 | 72.2 |
|  | *C.mitis* | 8250 | 81.5 |
|  | *C.mona* | 5300 | 69.3 |
|  | *C.ascanius* | 4500 | 71.5 |
|  | *C.talopoin* | 1380 | 41.1 |
|  | *Macaca sinica* | 8392 | 84 |
|  | *M.nemestrina* | 8610 | 122 |
|  | *M.fascicularis* | 7080 | 80.5 |
|  | *M.mulatta* | 8719 | 106.4 |
|  | *Cercocebus albigena* | 10500 | 116 |
|  | *C.torquatus* | 8680 | 140 |
|  | *C.galeitus* | 10700 | 118.5 |
|  | *Papio cynocephalus* | 22220 | 213 |
|  | *P.anubis* | 35000 | 222 |
|  | *P.papio* | 21800 | 193 |
|  | *P.ursinus* | 24490 | 181 |
|  | *P.hamadryas* | 16000 | 179 |
|  | *Mandrillus sphinx* | 32000 | 179 |
|  | *Presbytis entellus* | 21319 | 119.4 |
|  | *P.cristatus* | 16500 | 81.7 |
|  | *P.obscurus* | 7030 | 64.7 |
|  | *Hylobates lar* | 5700 | 105 |
|  | *H.moloch* | 6228 | 97.4 |
|  | *H.agilis* | 7372 | 87.5 |
|  | *Symphalangus syndactylus* | 12744 | 133 |
|  | *Pongo pygmaeus* | 90720 | 395 |
|  | *Pan troglodytes* | 56690 | 440 |
|  | *Gorilla gorilla* | 172370 | 570 |
|  | *Pongo pygmaeus* | 44452 | 287.5 |
|  | *pan troglodytes* | 43990 | 325 |
|  | *Gorilla gorilla* | 90720 | 426 |
|  | *Homo sapiens* | 55500 | 1361 |
|  | *Ateles paniscus* | 3430 | 108.96 |
|  | *Cebus albifrons* | 1620 | 62.95 |
|  | *Macaca nigra* | 3452 | 97.5 |
|  | *Macaca nemestrina* | 4456 | 110 |
|  | *Cebus capucinus* | 2104.875 | 70.135 |
|  | *Macaca assamensis* | 3655 | 90.5 |
|  | *Cebus apella* | 2589 | 71.2988 |
|  | *Cebus olivaceus* | 2684.5 | 72.5 |
|  | *Lagothrix lagotricha* | 3905 | 89.35 |
|  | *Macaca sinica* | 1970 | 58.15 |
|  | *Theropithecus gelada* | 7710 | 130 |
|  | *Cercocebus agilis* | 4700 | 95.3 |
|  | *Mandrillus sphinx* | 11500 | 159.2 |
|  | *Ateles geoffroyi* | 5774 | 104.9625 |
|  | *Lophocebus albigena* | 5125 | 96.8 |
|  | *Pan paniscus* | 39700 | 329.7016 |
|  | *Cercopithecus cephus* | 3508.33333 | 76 |
|  | *Macaca mulatta* | 4612.77778 | 87.99418 |
|  | *Semnopithecus entellus* | 7010 | 111.5 |
|  | *Cercopithecus mona* | 3001 | 67 |
|  | *Macaca fascicularis* | 3109.45455 | 66.92727 |
|  | *Hylobates lar* | 5550 | 93.994 |
|  | *Hylobates muelleri* | 5954.875 | 95.3125 |
|  | *Saimiri sciureus* | 578.7 | 23.34632 |
|  | *Papio hamadryas* | 12020 | 142 |
|  | *Hylobates agilis* | 5528.75 | 88.1 |
|  | *Pongo pygmaeus* | 54229.04 | 341.98994 |
|  | *Chlorocebus aethiops* | 3452.66667 | 64.13333 |
|  | *Saimiri oerstedii* | 605 | 22.45 |
|  | *Ateles fusciceps* | 9026.5 | 113.6 |
|  | *Aotus lemurinus* | 9026.5 | 113.5 |
|  | *Pan troglodytes* | 60433.15789 | 354.80966 |
|  | *Erythrocebus patas* | 7376 | 100.2 |
|  | *Hylobates syndactylus* | 12172 | 134.8 |
|  | *Macaca maura* | 6846 | 94.48 |
|  | *Macaca arctoides* | 7630 | 100.7 |
|  | *Saimiri boliviensis* | 750 | 24.06 |
|  | *Pithecia monachus* | 1500 | 35 |
|  | *Gorilla gorilla* | 120975 | 454.55 |
|  | *Trachypithecus francoisi* | 9100 | 94.4 |
|  | *Cercopithecus mitis* | 6300 | 75 |
|  | *Eulemur rubriventer* | 1015 | 24.9 |
|  | *Alouatta seniculus* | 2827.5 | 45.5 |
|  | *Daubentonia madagascariensis* | 2800 | 45.15 |
|  | *Procolobus badius* | 7000 | 78 |
|  | *Pygathrix nemaeus* | 7500 | 77 |
|  | *Colobus guereza* | 10281.25 | 83.9 |
|  | *Macaca sylvanus* | 11200 | 87.7 |
|  | *Callicebus moloch* | 900 | 19 |
|  | *Aotus trivirgatus* | 701.68 | 16.04 |
|  | *Leontopithecus rosalia* | 512.375 | 13.05 |
|  | *Alouatta caraya* | 5012.5 | 50.7 |
|  | *Colobus angolensis* | 9670 | 74.4 |
|  | *Saguinus oedipus* | 327.14286 | 9.64286 |
|  | *Saguinus geoffroyi* | 634.66667 | 14.27333 |
|  | *Eulemur mongoz* | 1559.33333 | 24.03333 |
|  | *Varecia variegata* | 2705.5 | 31.2 |
|  | *Alouatta palliata* | 5952 | 50.035 |
|  | *Nycticebus coucang* | 655.5 | 12.7425 |
|  | *Callithrix pygmaea* | 134.75 | 4.64 |
|  | *Galagoides demidoff* | 81 | 3.38 |
|  | *Callithrix jacchus* | 347.45455 | 7.73182 |
|  | *Eulemur macaco* | 2086.16667 | 22.6 |
|  | *Lemur catta* | 2090 | 21.63333 |
|  | *Indri indri* | 6250 | 38.3 |
|  | *Propithecus verreauxi* | 3480 | 26.7 |
|  | *Perodicticus potto* | 929.33333 | 12.06667 |
|  | *Tarsius bancanus* | 77.6 | 2.7 |
|  | *Galago senegalensis* | 300.33333 | 5.9 |
|  | *Nycticebus pygmaeus* | 480 | 7.8 |
|  | *Loris tardigradus* | 322 | 6 |
|  | *Cheirogaleus major* | 450 | 6.8 |
|  | *Microcebus murinus* | 58 | 1.84 |
|  | *Avahi laniger* | 1285 | 10.49 |
|  | *Cheirogaleus medius* | 179.66667 | 3.34333 |
| **Low level mammals** | *Elephas* | 2.76546E6 | 5211.25 |
|  | *Helarctos* | 45020 | 385.5 |
|  | *Loxodonta* | 4.30134E6 | 5436.4 |
|  | *Arctocephalus* | 45950 | 291.25 |
|  | *Zalophus* | 91000 | 405 |
|  | *Phoca* | 73635 | 315 |
|  | *Bassariscus* | 2722 | 41.9 |
|  | *Lobodon* | 222250 | 586.25 |
|  | *Vulpes* | 3722.70588 | 49.64 |
|  | *Equus* | 250000 | 612 |
|  | *Odobenus* | 1.02223E6 | 1410.25 |
|  | *Ommatophoca* | 179400 | 495 |
|  | *Arctocephalus* | 96600 | 340 |
|  | *Lynx* | 6350 | 65 |
|  | *Rangifer* | 71700 | 278 |
|  | *Arctocephalus* | 140000 | 415 |
|  | *Hydrurga* | 345500 | 712.5 |
|  | *Damaliscus* | 62500 | 254.6 |
|  | *Arctocephalus* | 101250 | 326.25 |
|  | *Potos* | 2241.5 | 33.075 |
|  | *Otaria* | 222000 | 506.25 |
|  | *Arctocephalus* | 109690 | 320 |
|  | *Arctocephalus* | 103750 | 307.5 |
|  | *Nasua* | 3187.5 | 37.5 |
|  | *Panthera* | 84000 | 259.5 |
|  | *Alces* | 200000 | 436 |
|  | *Tayassu* | 32233.33333 | 145.8 |
|  | *Ursus* | 131022.66667 | 335.96667 |
|  | *Connochaetes* | 212230 | 443 |
|  | *Tapirus* | 14260 | 85 |
|  | *Odocoileus* | 65090 | 210 |
|  | *Cervus* | 200000 | 409.3 |
|  | *Phoca* | 87580 | 248.75 |
|  | *Tragelaphus* | 44225 | 165 |
|  | *Phoca* | 91500 | 253.75 |
|  | *Urocyon* | 3749 | 37.28 |
|  | *Monachus* | 173000 | 370 |
|  | *Neophoca* | 189275 | 388.75 |
|  | *Ailurus* | 5590 | 46.8 |
|  | *Callorhinus* | 150916.66667 | 334.83333 |
|  | *Arctocephalus* | 178750 | 369.375 |
|  | *Equus* | 234000 | 434 |
|  | *Leopardus* | 9525.5 | 63.1 |
|  | *Monachus* | 280500 | 480 |
|  | *Leptonychotes* | 378666.66667 | 564.16667 |
|  | *Ursus* | 317000 | 507 |
|  | *Canis* | 8000 | 54.8 |
|  | *Procyon* | 4975.66667 | 41.06667 |
|  | *Erignathus* | 281000 | 460 |
|  | *Phoca* | 69085 | 196.25 |
|  | *Cystophora* | 282840 | 455 |
|  | *Aepyceros* | 57610 | 175 |
|  | *Boselaphus* | 125000 | 271.75 |
|  | *Axis* | 88450 | 219 |
|  | *Madoqua* | 4570 | 37 |
|  | *Rattus* | 359.5 | 8.025 |
|  | *Nasua* | 6250 | 44.17 |
|  | *Crocuta* | 62370 | 175 |
|  | *Neurotrichus* | 10 | 0.91 |
|  | *Myrmecophaga* | 18940 | 84 |
|  | *Callosciurus* | 240 | 6.02 |
|  | *Agouti* | 4607 | 35.21667 |
|  | *Paradoxurus* | 2773.5 | 25.95 |
|  | *Tragelaphus* | 253000 | 389 |
|  | *Melursus* | 136080 | 267 |
|  | *Puma* | 54432 | 154 |
|  | *Mustela* | 156.43333 | 4.57 |
|  | *Lynx* | 14969 | 69.5 |
|  | *Tamiops* | 39 | 1.95 |
|  | *Eumetopias* | 643775 | 661.25 |
|  | *Gerbillus* | 20.5 | 1.32 |
|  | *Phocarctos* | 273500 | 393.75 |
|  | *Phoca* | 62750 | 162.5 |
|  | *Gazella* | 24370 | 91.8 |
|  | *Xerus* | 400 | 7.65 |
|  | *Panthera* | 48000 | 135 |
|  | *Pteropus* | 374.85714 | 7.30143 |
|  | *Syncerus* | 665440 | 647.5 |
|  | *Halichoerus* | 194000 | 307.5 |
|  | *Dremomys* | 240 | 5.41 |
|  | *Tupaia* | 70 | 2.58 |
|  | *Otocyon* | 3335 | 26.09 |
|  | *Phoca* | 89500 | 187.5 |
|  | *Leptailurus* | 11340 | 54.1 |
|  | *Mirounga* | 2.0065E6 | 1205 |
|  | *Megaptera* | 3.005E7 | 6100 |
|  | *Callosciurus* | 209.5 | 4.91 |
|  | *Kobus* | 188200 | 290 |
|  | *Gymnobelideus* | 90.5 | 2.9526 |
|  | *Marmota* | 1890 | 18.1 |
|  | *Pteropus* | 314.28571 | 6.12857 |
|  | *Genetta* | 1525 | 15.35 |
|  | *Tscherskia* | 25 | 1.29 |
|  | *Sciurus* | 516.31667 | 7.91167 |
|  | *Procavia* | 2321.66667 | 19.46667 |
|  | *Sus* | 86772.72727 | 169.8 |
|  | *Ichneumia* | 4400 | 28.3 |
|  | *Callosciurus* | 202 | 4.44 |
|  | *Sciurus* | 327.4 | 5.925 |
|  | *Funisciurus* | 200 | 4.38 |
|  | *Panthera* | 195000 | 269.73333 |
|  | *Ammospermophilus* | 70 | 2.31 |
|  | *Tamandua* | 4361 | 27.5 |
|  | *Heliosciurus* | 291 | 5.4 |
|  | *Dasyprocta* | 2873 | 21.26 |
|  | *Heliosciurus* | 326 | 5.75 |
|  | *Sciurus* | 503.16667 | 7.41 |
|  | *Moschiola* | 1997 | 16.925 |
|  | *Zaglossus* | 7500 | 37.34677 |
|  | *Atherurus* | 3152.5 | 21.885 |
|  | *Tamias* | 51.2 | 1.83 |
|  | *Onychogalea* | 1433 | 13.5 |
|  | *Callosciurus* | 439 | 6.52 |
|  | *Bradypus* | 4097 | 24.9 |
|  | *Tamias* | 60.8 | 1.99 |
|  | *Sciurus* | 595 | 7.81 |
|  | *Tragulus* | 2510 | 18.5 |
|  | *Choloepus* | 5070 | 28.2 |
|  | *Thylogale* | 3618 | 22.8956 |
|  | *Bettongia* | 1273 | 12.1212 |
|  | *Tamias* | 62.2 | 1.98 |
|  | *Cabassous* | 3930 | 23.68 |
|  | *Tamiasciurus* | 188.9 | 3.83 |
|  | *Tamias* | 89.4 | 2.44 |
|  | *Funisciurus* | 218 | 4.14 |
|  | *Iomys* | 155.5 | 3.38 |
|  | *Scalopus* | 39.6 | 1.48 |
|  | *Tamias* | 45.3 | 1.6 |
|  | *Sciurus* | 400 | 5.91 |
|  | *Phacochoerus* | 65320 | 125 |
|  | *Xerus* | 317.5 | 5.09 |
|  | *Rhinosciurus* | 240 | 4.28 |
|  | *Jaculus* | 98 | 2.5 |
|  | *Tamias* | 59.9 | 1.86 |
|  | *Macropus* | 4425 | 24.5532 |
|  | *Hydrochaeris* | 28500 | 75 |
|  | *Bettongia* | 981 | 9.90416 |
|  | *Scapanus* | 70 | 2.02 |
|  | *Ratufa* | 982.5 | 9.85 |
|  | *Thryonomys* | 1625 | 13.28 |
|  | *Giraffa* | 1.209E6 | 700 |
|  | *Proechimys* | 544.75 | 6.87 |
|  | *Funisciurus* | 268 | 4.48 |
|  | *Rhynchocyon* | 370 | 5.4 |
|  | *Dendrolagus* | 7897 | 33.4628 |
|  | *Tamias* | 82.3 | 2.16 |
|  | *Euphractus* | 7990 | 33.5 |
|  | *Lepus* | 1954 | 14.35 |
|  | *Hippopotamus* | 1.351E6 | 720 |
|  | *Lagorchestes* | 2015 | 14.504 |
|  | *Spermophilopsis* | 495 | 6.18 |
|  | *Allactaga* | 193 | 3.5 |
|  | *Ratufa* | 1320 | 11.01 |
|  | *Aeromys* | 1189 | 10.34 |
|  | *Choloepus* | 5048 | 24.535 |
|  | *Urogale* | 275 | 4.28 |
|  | *Petrogale* | 1407 | 11.396 |
|  | *Tupaia* | 170 | 3.2 |
|  | *Dasyprocta* | 3172 | 18.34 |
|  | *Funambulus* | 85.9 | 2.1 |
|  | *Dactylopsila* | 506 | 6.03988 |
|  | *Thylogale* | 3694 | 19.8912 |
|  | *Tamias* | 62.3 | 1.7 |
|  | *Ammospermophilus* | 105.9 | 2.33 |
|  | *Tamias* | 94.3 | 2.17 |
|  | *Erethizon* | 5397 | 24.6 |
|  | *Balaenoptera* | 3.66667E7 | 4900 |
|  | *Rhynchocyon* | 507.5 | 5.9 |
|  | *Rousettus* | 130 | 2.6 |
|  | *Lagidium* | 2460.5 | 15.075 |
|  | *Dolichotis* | 8000 | 30.5 |
|  | *Tachyglossus* | 4250 | 20.73437 |
|  | *Glaucomys* | 174 | 3.01 |
|  | *Macropus* | 12257.5 | 38.148 |
|  | *Petrogale* | 5990 | 24.7604 |
|  | *Vombatus* | 27192 | 61.124 |
|  | *Macropus* | 15194 | 42.7868 |
|  | *Tamias* | 50.8 | 1.39 |
|  | *Mustela* | 915 | 7.87 |
|  | *Heliosciurus* | 295 | 3.99 |
|  | *Ornithorhynchus* | 1389 | 10.08323 |
|  | *Condylura* | 50 | 1.37 |
|  | *Melomys* | 83.4 | 1.86 |
|  | *Petaurista* | 1811.5 | 11.765 |
|  | *Glaucomys* | 73.1 | 1.71 |
|  | *Cavia* | 306.45 | 4.035 |
|  | *Spermophilus* | 209.7 | 3.20656 |
|  | *Petaurista* | 924 | 7.76 |
|  | *Xerus* | 722.5 | 6.68 |
|  | *Trichosurus* | 1761 | 11.398 |
|  | *Hylopetes* | 87.5 | 1.87 |
|  | *Sylvilagus* | 712.4 | 6.58 |
|  | *Dipodomys* | 57.1 | 1.44628 |
|  | *Stenomys* | 59.4 | 1.48 |
|  | *Tatera* | 65 | 1.56 |
|  | *Hoplomys* | 330 | 4.13 |
|  | *Aplodontia* | 806 | 7.04 |
|  | *Lepus* | 2314.9 | 13.23 |
|  | *Pogonomelomys* | 61.4 | 1.49 |
|  | *Jaculus* | 64.2 | 1.53 |
|  | *Ochotona* | 120 | 2.22 |
|  | *Bradypus* | 3263 | 16.03333 |
|  | *Gerbillurus* | 24 | 0.84 |
|  | *Macropus* | 3760 | 17.4 |
|  | *Caluromys* | 277 | 3.626 |
|  | *Petrogale* | 5732 | 22.274 |
|  | *Dendrolagus* | 10119 | 31.1836 |
|  | *Gerbillus* | 27.5 | 0.9 |
|  | *Gerbillus* | 14 | 0.6 |
|  | *Melomys* | 49.7 | 1.27 |
|  | *Ratufa* | 1935 | 11.4 |
|  | *Lepus* | 1961 | 11.49 |
|  | *Thylamys* | 20.1 | 0.73556 |
|  | *Pogonomys* | 41.2 | 1.13 |
|  | *Dipodomys* | 39.4 | 1.09998 |
|  | *Lepus* | 1500 | 9.72 |
|  | *Dipodomys* | 74 | 1.59544 |
|  | *Myrmecobius* | 405.4 | 4.403 |
|  | *Cavia* | 476 | 4.83333 |
|  | *Desmodus* | 29 | 0.898 |
|  | *Microdipodops* | 12.9 | 0.54908 |
|  | *Macropus* | 31286 | 58.8448 |
|  | *Elephantulus* | 57 | 1.33 |
|  | *Caluromys* | 289 | 3.5224 |
|  | *Lepus* | 3274.5 | 15.095 |
|  | *Sylvilagus* | 1036 | 7.56 |
|  | *Galemys* | 57.5 | 1.33 |
|  | *Melomys* | 54.7 | 1.29 |
|  | *Sylvilagus* | 439 | 4.5 |
|  | *Cynopterus* | 29 | 0.88 |
|  | *Dipodomys* | 65.65 | 1.4361 |
|  | *Stenomys* | 42.1 | 1.1 |
|  | *Ammospermophilus* | 138.8 | 2.24 |
|  | *Spermophilus* | 262.9 | 3.28 |
|  | *Diaemus* | 34.6 | 0.97 |
|  | *Microdipodops* | 13.85 | 0.559 |
|  | *Phalanger* | 1672 | 9.88344 |
|  | *Spermophilus* | 156.4 | 2.3757 |
|  | *Podomys* | 33.46667 | 0.94 |
|  | *Macropus* | 35540 | 61.124 |
|  | *Cynopterus* | 53 | 1.23 |
|  | *Dipodomys* | 61.4 | 1.34 |
|  | *Paraxerus* | 216 | 2.85 |
|  | *Neotoma* | 193.3 | 2.66 |
|  | *Castor* | 27670 | 52.21 |
|  | *Eonycteris* | 50 | 1.18 |
|  | *Peromyscus* | 77.32 | 1.53 |
|  | *Spermophilus* | 587.5 | 5.12 |
|  | *Rhyncholestes* | 20 | 0.6734 |
|  | *Spilocuscus* | 3127 | 13.8824 |
|  | *Pseudomys* | 50 | 1.16 |
|  | *Peromyscus* | 13.56 | 0.53 |
|  | *Oryctolagus* | 2157.6 | 11.09333 |
|  | *Ototylomys* | 63 | 1.33 |
|  | *Sylvilagus* | 792.5 | 6.07 |
|  | *Ochotona* | 169 | 2.39 |
|  | *Notomys* | 37 | 0.96 |
|  | *Onychomys* | 27.385 | 0.80083 |
|  | *Noctilio* | 58.5 | 1.26 |
|  | *Cynomys* | 793.5 | 6.01 |
|  | *Phyllostomus* | 33 | 0.89 |
|  | *Cercartetus* | 7.8 | 0.37296 |
|  | *Neotoma* | 257.7 | 3.04 |
|  | *Rattus* | 92.7 | 1.64 |
|  | *Pogonomys* | 73.9 | 1.43 |
|  | *Trichechus* | 756000 | 364 |
|  | *Pseudocheirus* | 759 | 5.78088 |
|  | *Peromyscus* | 52.83 | 1.16 |
|  | *Balaenoptera* | 6.25E7 | 5100 |
|  | *Mephitis* | 1980 | 10.15 |
|  | *Rattus* | 101 | 1.7 |
|  | *Marmosa* | 60 | 1.2432 |
|  | *Parantechinus* | 60.5 | 1.2432 |
|  | *Artibeus* | 37.5 | 0.93 |
|  | *Peroryctes* | 977 | 6.55788 |
|  | *Lepus* | 4231 | 15.8 |
|  | *Rattus* | 76.2 | 1.41 |
|  | *Spalacopus* | 93 | 1.58 |
|  | *Leopoldamys* | 332.7 | 3.39 |
|  | *Grammomys* | 40.7 | 0.96 |
|  | *Desmana* | 440 | 4 |
|  | *Tatera* | 91.7 | 1.56 |
|  | *Marmosa* | 46.7 | 1.036 |
|  | *Peromyscus* | 27.35 | 0.75 |
|  | *Hypsignathus* | 340 | 3.4 |
|  | *Talpa* | 77.83333 | 1.40333 |
|  | *Antechinus* | 29.8 | 0.78736 |
|  | *Clethrionomys* | 16.9 | 0.56 |
|  | *Melomys* | 70 | 1.31 |
|  | *Massoutiera* | 182.6 | 2.32 |
|  | *Dorcopsis* | 7392 | 21.3416 |
|  | *Rattus* | 134.1 | 1.92 |
|  | *Spermophilus* | 343.66667 | 3.37385 |
|  | *Dipodomys* | 62.8 | 1.21624 |
|  | *Peromyscus* | 46.55 | 1.015 |
|  | *Malacomys* | 60 | 1.18 |
|  | *Metachirus* | 390 | 3.626 |
|  | *Peromyscus* | 39.73 | 0.92 |
|  | *Chrysochloris* | 50.05 | 1.055 |
|  | *Chiropodomys* | 25.3 | 0.7 |
|  | *Lepus* | 2412.5 | 10.78 |
|  | *Acomys* | 18.5 | 0.58 |
|  | *Mystromys* | 80 | 1.39 |
|  | *Clethrionomys* | 17.6 | 0.56 |
|  | *Phalanger* | 1674 | 8.60916 |
|  | *Ochotona* | 250 | 2.75 |
|  | *Casinycteris* | 40.5 | 0.92 |
|  | *Phalanger* | 1754 | 8.79564 |
|  | *Meriones* | 57.4 | 1.13 |
|  | *Dasycercus* | 98.4 | 1.554 |
|  | *Uroderma* | 16.4 | 0.53 |
|  | *Zapus* | 17.46667 | 0.54937 |
|  | *Distoechurus* | 45.4 | 0.97384 |
|  | *Onychomys* | 20.02 | 0.59466 |
|  | *Peromyscus* | 26.3 | 0.7 |
|  | *Praomys* | 37.2 | 0.86 |
|  | *Spermophilus* | 763.72 | 5.24554 |
|  | *Marmosa* | 60 | 1.1396 |
|  | *Phascogale* | 43.8 | 0.94276 |
|  | *Pteromyscus* | 400 | 3.55 |
|  | *Dipodomys* | 144.55 | 1.92744 |
|  | *Gerbillus* | 26.1 | 0.69 |
|  | *Peromyscus* | 27.47 | 0.71 |
|  | *Dipodomys* | 122.45 | 1.74024 |
|  | *Spermophilus* | 455.5 | 3.82 |
|  | *Wallabia* | 16400 | 32.7 |
|  | *Hybomys* | 57.5 | 1.1 |
|  | *Apodemus* | 20.1 | 0.585 |
|  | *Platyrrhinus* | 11.6 | 0.42 |
|  | *Reithrodontomys* | 10.7 | 0.4 |
|  | *Lophuromys* | 60.7 | 1.13 |
|  | *Microtus* | 30.13333 | 0.742 |
|  | *Peromyscus* | 21.21 | 0.6 |
|  | *Rattus* | 120.66667 | 1.7 |
|  | *Peromyscus* | 45 | 0.94 |
|  | *Cricetomys* | 1150 | 6.57 |
|  | *Micromys* | 6.4 | 0.29 |
|  | *Tatera* | 139.7 | 1.84 |
|  | *Gerbillus* | 40.9 | 0.88 |
|  | *Myoxus* | 148 | 1.9 |
|  | *Sturnira* | 17.1 | 0.52 |
|  | *Rattus* | 122 | 1.69 |
|  | *Orthogeomys* | 405 | 3.4706 |
|  | *Peromyscus* | 43.53333 | 0.91 |
|  | *Neofiber* | 270.5 | 2.72 |
|  | *Perognathus* | 8.15 | 0.33058 |
|  | *Cynomys* | 796.3 | 5.16 |
|  | *Oligoryzomys* | 26.6 | 0.67 |
|  | *Antechinus* | 63.5 | 1.12924 |
|  | *Uranomys* | 33.6 | 0.77 |
|  | *Spermophilus* | 725.3 | 4.84 |
|  | *Arvicanthis* | 84.5 | 1.33 |
|  | *Liomys* | 57.7 | 1.05672 |
|  | *Nyctomys* | 60 | 1.08 |
|  | *Phalanger* | 1448 | 7.27272 |
|  | *Clethrionomys* | 17.9 | 0.52 |
|  | *Chaetodipus* | 29.75 | 0.7017 |
|  | *Peromyscus* | 28.25 | 0.68 |
|  | *Glossophaga* | 11.2 | 0.39 |
|  | *Murexia* | 62 | 1.0878 |
|  | *Artibeus* | 58.7 | 1.05 |
|  | *Microtus* | 32.4 | 0.735 |
|  | *Spermophilus* | 488.2 | 3.7394 |
|  | *Orthogeomys* | 542.1 | 3.97824 |
|  | *Peromyscus* | 20.72 | 0.56 |
|  | *Cynomys* | 992.1 | 5.69 |
|  | *Peromyscus* | 66.2 | 1.12 |
|  | *Orthogeomys* | 630 | 4.30976 |
|  | *Peromyscus* | 21.65 | 0.57 |
|  | *Zygogeomys* | 545 | 3.94716 |
|  | *Peromyscus* | 19.93 | 0.54 |
|  | *Peromyscus* | 58.8 | 1.03 |
|  | *Carollia* | 16 | 0.47 |
|  | *Isthmomys* | 138 | 1.71 |
|  | *Antechinus* | 36.3 | 0.76664 |
|  | *Peromyscus* | 13.31667 | 0.42 |
|  | *Microtus* | 45.8 | 0.88 |
|  | *Chaetodipus* | 20.3 | 0.53872 |
|  | *Philander* | 570 | 3.96788 |
|  | *Synaptomys* | 23.8 | 0.59 |
|  | *Perognathus* | 17.3 | 0.48692 |
|  | *Chaetodipus* | 26 | 0.6216 |
|  | *Spermophilus* | 156.6 | 1.82 |
|  | *Dinomys* | 14000 | 26.95 |
|  | *Nycteris* | 10.6 | 0.36 |
|  | *Caluromys* | 384 | 3.1 |
|  | *Oryzomys* | 47.1 | 0.88 |
|  | *Apodemus* | 32.3 | 0.7 |
|  | *Pappogeomys* | 637 | 4.18544 |
|  | *Chaetodipus* | 16.25 | 0.46292 |
|  | *Chaetodipus* | 18.1 | 0.49274 |
|  | *Lophuromys* | 60 | 1.01 |
|  | *Sminthopsis* | 17.8 | 0.48692 |
|  | *Geocapromys* | 717.3 | 4.46 |
|  | *Spermophilus* | 199 | 2.06 |
|  | *Pectinator* | 180 | 1.93 |
|  | *Lemniscomys* | 46.2 | 0.85 |
|  | *Perognathus* | 8.4 | 0.3054 |
|  | *Hydromys* | 698 | 4.33 |
|  | *Antechinus* | 30.7 | 0.66304 |
|  | *Sminthopsis* | 19.8 | 0.50764 |
|  | *Heteromys* | 74.65 | 1.12534 |
|  | *Neotoma* | 223.1 | 2.17 |
|  | *Perameles* | 457 | 3.32556 |
|  | *Lagostomus* | 4270.75 | 12.66 |
|  | *Praomys* | 46.5 | 0.84 |
|  | *Myocastor* | 7052 | 17.09 |
|  | *Potamogale* | 650 | 4.0455 |
|  | *Holochilus* | 112.3 | 1.41 |
|  | *Dasyurus* | 513 | 3.50168 |
|  | *Microgale* | 15.2 | 0.42 |
|  | *Petauroides* | 900 | 4.85884 |
|  | *Isoodon* | 822 | 4.57912 |
|  | *Neotoma* | 331.3 | 2.65 |
|  | *Microtus* | 22 | 0.52 |
|  | *Microtus* | 24.9 | 0.56 |
|  | *Microtus* | 43.3 | 0.78 |
|  | *Chaetodipus* | 19.1 | 0.47656 |
|  | *Mormoops* | 15.6 | 0.42 |
|  | *Leptonycteris* | 24.5 | 0.55 |
|  | *Liomys* | 43.35 | 0.7735 |
|  | *Myomys* | 35 | 0.68 |
|  | *Cercartetus* | 9.5 | 0.3108 |
|  | *Isoodon* | 691 | 4.03004 |
|  | *Geomys* | 313.5 | 2.50712 |
|  | *Marmota* | 3624.75 | 10.825 |
|  | *Myotis* | 7.5 | 0.265 |
|  | *Saccostomus* | 49.35 | 0.82 |
|  | *Nannospalax* | 197 | 1.88 |
|  | *Sminthopsis* | 13.3 | 0.37296 |
|  | *Solenodon* | 900 | 4.67 |
|  | *Liomys* | 44.9 | 0.77 |
|  | *Chaetodipus* | 37.1 | 0.68188 |
|  | *Galea* | 375 | 2.72 |
|  | *Sorex* | 5.3 | 0.21 |
|  | *Hipposideros* | 10.7 | 0.32 |
|  | *Geomys* | 197.25 | 1.82808 |
|  | *Monodelphis* | 64.6 | 0.9324 |
|  | *Sorex* | 8.2 | 0.27 |
|  | *Limnogale* | 92 | 1.15 |
|  | *Sorex* | 6.3 | 0.23 |
|  | *Atelerix* | 280 | 2.24 |
|  | *Rattus* | 60.3 | 0.89 |
|  | *Thomomys* | 105.65 | 1.24408 |
|  | *Microtus* | 39.7 | 0.69 |
|  | *Rhabdomys* | 39.70952 | 0.69 |
|  | *Arvicola* | 168.3 | 1.64 |
|  | *Sminthopsis* | 17 | 0.4144 |
|  | *Neotoma* | 378.3 | 2.66 |
|  | *Pteronotus* | 10.6 | 0.31 |
|  | *Microgale* | 50.4 | 0.79 |
|  | *Sorex* | 3.9 | 0.17 |
|  | *Meriones* | 71.3 | 0.97 |
|  | *Beamys* | 89.75 | 1.11 |
|  | *Megadontomys* | 111 | 1.26 |
|  | *Cryptomys* | 98.4 | 1.17 |
|  | *Macrotis* | 1859 | 6.80652 |
|  | *Didelphis* | 1535 | 6.05 |
|  | *Ondatra* | 1136.45 | 5.03 |
|  | *Perameles* | 1002 | 4.662 |
|  | *Dasypus* | 2743.5 | 8.5 |
|  | *Microtus* | 43.8 | 0.71 |
|  | *Zapus* | 24.5 | 0.5 |
|  | *Napaeozapus* | 23.2 | 0.48 |
|  | *Cricetus* | 297 | 2.2 |
|  | *Rhinolophus* | 7.15 | 0.235 |
|  | *Aethomys* | 117 | 1.25 |
|  | *Myotis* | 7 | 0.23 |
|  | *Aethomys* | 146.3 | 1.42 |
|  | *Tachyoryctes* | 234 | 1.88 |
|  | *Microtus* | 30.4 | 0.55 |
|  | *Otomys* | 141 | 1.38 |
|  | *Erinaceus* | 720.43636 | 3.66545 |
|  | *Perameles* | 1273 | 5.13856 |
|  | *Sorex* | 11.9 | 0.31 |
|  | *Echymipera* | 798 | 3.86428 |
|  | *Myotis* | 4 | 0.16 |
|  | *Myotis* | 25 | 0.48 |
|  | *Lagurus* | 20.1 | 0.42 |
|  | *Peromyscus* | 39.6 | 0.63 |
|  | *Thomomys* | 162.95 | 1.47164 |
|  | *Surdisorex* | 18.6 | 0.4 |
|  | *Microgale* | 32.6 | 0.56 |
|  | *Hemiechinus* | 250 | 1.9 |
|  | *Microtus* | 47.4 | 0.7 |
|  | *Marmosa* | 50.5 | 0.7252 |
|  | *Clethrionomys* | 38.3 | 0.61 |
|  | *Myotis* | 7 | 0.22 |
|  | *Mesembriomys* | 1110 | 4.59 |
|  | *Myopus* | 33.5 | 0.56 |
|  | *Heliophobius* | 160 | 1.43 |
|  | *Lemmus* | 68.9 | 0.86 |
|  | *Chrysochloris* | 49 | 0.7 |
|  | *Mimon* | 14.8 | 0.34 |
|  | *Hipposideros* | 8.4 | 0.24 |
|  | *Antechinus* | 60.2 | 0.777 |
|  | *Mus* | 29.2 | 0.5 |
|  | *Antechinus* | 45.8 | 0.65268 |
|  | *Echymipera* | 1207 | 4.64128 |
|  | *Microtus* | 51.3 | 0.69 |
|  | *Aethomys* | 79.4 | 0.89 |
|  | *Dicrostonyx* | 73 | 0.84 |
|  | *Saccopteryx* | 8 | 0.22 |
|  | *Miniopterus* | 12.7 | 0.29 |
|  | *Sorex* | 5.3 | 0.17 |
|  | *Myotis* | 7.6 | 0.21 |
|  | *Marmota* | 5000 | 10.29 |
|  | *Mus* | 14.6 | 0.31 |
|  | *Noctilio* | 38.5 | 0.55 |
|  | *Chaerephon* | 13.3 | 0.29 |
|  | *Myotis* | 15 | 0.31 |
|  | *Sigmodon* | 132.5 | 1.14 |
|  | *Sorex* | 8.9 | 0.225 |
|  | *Oryzorictes* | 44.2 | 0.58 |
|  | *Didelphis* | 2797 | 6.96192 |
|  | *Planigale* | 6.1 | 0.17612 |
|  | *Atelerix* | 790 | 3.2 |
|  | *Murexia* | 296.5 | 1.74048 |
|  | *Dicrostonyx* | 68.4 | 0.72 |
|  | *Neomys* | 16.35 | 0.305 |
|  | *Sorex* | 8.9 | 0.21 |
|  | *Molossus* | 13 | 0.26 |
|  | *Setifer* | 248 | 1.51 |
|  | *Bathyergus* | 1175 | 3.75 |
|  | *Sorex* | 4.6 | 0.13 |
|  | *Blarina* | 17.7 | 0.28877 |
|  | *Notiosorex* | 6 | 0.15 |
|  | *Mormopterus* | 11.5 | 0.22 |
|  | *Hemicentetes* | 110 | 0.83 |
|  | *Myotis* | 4.5 | 0.12 |
|  | *Sorex* | 3.05 | 0.0935 |
|  | *Tenrec* | 907 | 2.835 |
|  | *Crocidura* | 10.45 | 0.19 |
|  | *Crocidura* | 5.6 | 0.13 |
|  | *Suncus* | 35 | 0.39 |
|  | *Rhynchonycteris* | 4.3 | 0.11 |
|  | *Sorex* | 5 | 0.12 |
|  | *Heterocephalus* | 60.8 | 0.52 |
|  | *Cryptotis* | 5.3 | 0.12 |
|  | *Suncus* | 2.24 | 0.071 |
|  | *Echinops* | 87.5 | 0.62 |
|  | *Otomys* | 95 | 0.42 |
|  | *Apodemus* | 33.3 | 0.2 |

**Table S1b: 600 bird species** [**^5^**](#_ENREF_5)

| **Species** | **Body (gram)** | **Brain (gram)** |
| --- | --- | --- |
| *Aegithalos caudatus* | 8.20 | 0.50 |
| *Aegithalos concinnus* | 6.10 | 0.38 |
| *Psaltriparus minimus* | 5.30 | 0.36 |
| *Eremophila bilopha* | 23.55 | 0.85 |
| *Galerida cristata* | 42.13 | 1.08 |
| *Mirafra javanica* | 23.00 | 0.76 |
| *Dulus dominicus* | 47.60 | 1.32 |
| *Auriparus flaviceps* | 6.60 | 0.46 |
| *Campylorhynchus bruneicapillus* | 38.90 | 1.39 |
| *Certhia familiaris* | 8.42 | 0.55 |
| *Cyphorhinus aradus* | 18.70 | 0.94 |
| *Donacobius atricapillus* | 34.80 | 1.19 |
| *Henicorhina leucosticta* | 13.90 | 0.81 |
| *Polioptila dumicola* | 7.60 | 0.43 |
| *Ramphocaenus melanurus* | 9.80 | 0.53 |
| *Thryomanes bewickii* | 9.80 | 0.54 |
| *Thryothurus ludovicianus* | 18.70 | 0.88 |
| *Cinclus mexicanus* | 57.80 | 1.45 |
| *Cisticola cherinus* | 10.00 | 0.45 |
| *Cisticola fulvicapilla* | 10.30 | 0.53 |
| *PriniaSchistolais leucopogon* | 13.80 | 0.61 |
| *Climacteris picumnus* | 30.30 | 1.04 |
| *Climacteris rufa* | 33.30 | 1.07 |
| *Cormobates leucophaeus* | 22.40 | 0.84 |
| *Conopophaga lineata* | 21.80 | 0.73 |
| *Aegithina tiphia* | 12.30 | 0.69 |
| *Aphelocoma caerulescens* | 76.00 | 2.95 |
| *Aphelocoma ultramarina* | 128.40 | 3.74 |
| *Artamus cinereus* | 38.00 | 0.95 |
| *Artamus cyanopterus* | 35.50 | 1.04 |
| *Artamus leucorhynchus* | 45.60 | 1.12 |
| *Artamus superciliosus* | 39.20 | 1.08 |
| *Batis capensis* | 12.80 | 0.70 |
| *Bias musicus* | 21.70 | 0.87 |
| *Campephaga phoenicea* | 28.50 | 1.09 |
| *Cicinnurus regius* | 52.00 | 1.80 |
| *Cinclosoma cinnamomeum* | 58.60 | 1.52 |
| *Cissa chinensis* | 106.50 | 4.33 |
| *Colluricincla harmonica* | 63.30 | 2.07 |
| *Coracina caledonica* | 145.30 | 3.10 |
| *Coracina novaehollandiae* | 127.70 | 2.49 |
| *Corcorax melanorhamphus* | 349.10 | 5.39 |
| *Corvus albicollis* | 900.00 | 12.00 |
| *Corvus albus* | 584.10 | 9.07 |
| *Corvus brachyrhynchos* | 438.50 | 7.43 |
| *Corvus corax* | 1405.00 | 15.31 |
| *Corvus corone* | 523.95 | 7.36 |
| *Corvus coronoides* | 675.00 | 10.18 |
| *Corvus cryptoleucos* | 534.00 | 9.27 |
| *Corvus frugilegus* | 509.30 | 8.13 |
| *Corvus mellori* | 300.00 | 8.81 |
| *Corvus monedula* | 211.29 | 4.69 |
| *Corvus orru* | 522.20 | 9.23 |
| *Corvus ossifragus* | 285.00 | 6.21 |
| *Cracticus torquatus* | 104.10 | 2.88 |
| *Cyanocitta cristata* | 89.10 | 3.03 |
| *Cyanocitta stelleri* | 128.00 | 3.67 |
| *Cyanocorax chrysops* | 157.00 | 4.30 |
| *Cyanocorax yncas* | 78.50 | 2.40 |
| *Cyanopica cyana* | 72.00 | 3.00 |
| *Dendrocitta vagabunda* | 100.00 | 2.80 |
| *Dryoscopus cubla* | 26.40 | 1.17 |
| *Falculuncus frontatus* | 26.10 | 1.28 |
| *Garrulus glandarius* | 168.89 | 4.15 |
| *Grallina cyanoleuca* | 89.00 | 1.74 |
| *Gymnorhina tibicen* | 314.00 | 4.82 |
| *Gymnorhinus cyanocephalus* | 103.00 | 3.64 |
| *Hypothymis azurea* | 10.20 | 0.54 |
| *Lalage sueurii* | 18.90 | 0.90 |
| *Laniarius erythrogaster* | 45.80 | 1.62 |
| *Lophorina superba* | 78.00 | 2.69 |
| *Monarcha guttula* | 15.50 | 0.70 |
| *Monarcha melanopsis* | 20.20 | 0.95 |
| *Monarcha trivirgatus* | 13.50 | 0.65 |
| *Myiagra alecto* | 24.00 | 0.76 |
| *Myiagra caledonica* | 11.00 | 0.61 |
| *Myiagra inquieta* | 14.30 | 0.79 |
| *Nucifraga caryocatactes* | 189.47 | 5.71 |
| *Oreoica gutturalis* | 62.00 | 1.73 |
| *Oriolus oriolus* | 71.90 | 1.50 |
| *Parotia lawesii* | 156.50 | 4.15 |
| *Pericrocotus ethologus* | 18.00 | 0.91 |
| *Perisoreus canadensis* | 69.00 | 2.62 |
| *Pica pica* | 206.13 | 5.34 |
| *Pitohui ferrugineus* | 94.00 | 2.57 |
| *Platysteira cyanea* | 11.84 | 0.60 |
| *Podoce hendersoni* | 149.00 | 3.40 |
| *Prionops plumatus* | 35.50 | 1.20 |
| *Psilorhinus morio* | 204.00 | 4.97 |
| *Psophodes occidentalis* | 42.00 | 1.38 |
| *Ptiloris paradiseus* | 125.50 | 2.88 |
| *Ptiloris victoriae* | 92.00 | 2.63 |
| *Pyrrhocorax graculus* | 223.50 | 3.20 |
| *Pyrrhocorax pyrrhocorax* | 324.00 | 6.61 |
| *Rhipidura leucophrys* | 27.70 | 0.60 |
| *Rhipidura rufifrons* | 10.20 | 0.37 |
| *Sphecotheres viridis* | 132.40 | 2.32 |
| *Strepera fuliginosa* | 300.00 | 5.91 |
| *Struthidea cinerea* | 134.30 | 3.09 |
| *Tchagra australis* | 36.30 | 1.27 |
| *Telophorus zeylonus* | 62.70 | 1.98 |
| *Tephrodornis pondicerianus* | 19.50 | 0.92 |
| *Urocissa erythrorhyncha* | 214.00 | 3.80 |
| *Smithornis capensis* | 25.50 | 0.81 |
| *Chamaeza campanisona* | 97.20 | 1.90 |
| *Formicarius analis* | 54.20 | 1.35 |
| *Formicarius colma* | 45.30 | 1.12 |
| *Grallaria quitensis* | 79.50 | 2.12 |
| *Agelaius phoeniceus* | 65.50 | 1.75 |
| *Agelaius ruficapillus* | 36.75 | 0.81 |
| *Aimophila cassinii* | 18.10 | 0.81 |
| *Amblycercus holosericeus* | 66.90 | 2.29 |
| *Amblyramphus holosericeus* | 69.00 | 2.41 |
| *Ammodramus savannarum* | 17.00 | 0.67 |
| *Amphispiza bilineata* | 13.80 | 0.70 |
| *Anisognathus somptuosus* | 42.00 | 1.54 |
| *Arremonops conirostris* | 37.30 | 1.34 |
| *Basileuterus culicivorus* | 8.90 | 0.60 |
| *Basileuterus fulvicauda* | 14.90 | 0.58 |
| *BuarremonAtlapetes brunneinucha* | 46.60 | 1.60 |
| *Cacicus cela* | 91.50 | 2.52 |
| *Calamospiza melanocorys* | 37.80 | 1.22 |
| *Calcarius lapponicus* | 27.50 | 0.90 |
| *Cardellina rubrifrons* | 9.80 | 0.45 |
| *Cardinalis cardinalis* | 44.10 | 1.57 |
| *Carduelis ambigua* | 14.10 | 0.66 |
| *Carduelis cannabina* | 18.78 | 0.67 |
| *Carduelis carduelis* | 15.00 | 0.59 |
| *Carduelis chloris* | 26.50 | 0.89 |
| *Carduelis flammea* | 12.13 | 0.60 |
| *Carduelis pinus* | 14.60 | 0.59 |
| *Carduelis spinus* | 12.08 | 0.56 |
| *Carduelis tristis* | 12.90 | 0.56 |
| *Carpodacus mexicanus* | 20.40 | 0.81 |
| *Carpodacus purpureus* | 25.60 | 0.93 |
| *Carpodacus roseus* | 21.15 | 1.01 |
| *Caryothraustes poliogaster* | 37.50 | 1.48 |
| *Chlorophonia cyanea* | 14.00 | 0.63 |
| *Chlorospingus ophthalmicus* | 19.00 | 1.02 |
| *Chlorothraupis carmioli* | 39.00 | 1.35 |
| *Cissopis leveriana* | 76.00 | 1.77 |
| *Coccothraustes coccothraustes* | 54.00 | 1.63 |
| *Coereba flaveola* | 8.50 | 0.46 |
| *Coryphospingus cucullatus* | 14.60 | 0.69 |
| *Curaeus curaeus* | 90.00 | 2.56 |
| *Cyanerpes cyaneus* | 14.00 | 0.57 |
| *Dacnis cayana* | 13.00 | 0.57 |
| *Dendroica caerulescens* | 9.40 | 0.45 |
| *Dendroica castanea* | 12.00 | 0.41 |
| *Dendroica magnolia* | 8.70 | 0.38 |
| *Dendroica pensylvanica* | 9.40 | 0.39 |
| *Dendroica petechia* | 9.80 | 0.54 |
| *Dives dives* | 96.20 | 2.28 |
| *Dolichonyx oryzivorus* | 38.70 | 1.11 |
| *Emberiza cirlus* | 23.10 | 0.79 |
| *Emberiza citrinella* | 28.58 | 0.82 |
| *Emberiza elegans* | 20.50 | 0.64 |
| *Emberiza rutilla* | 17.18 | 0.59 |
| *Emberiza schoeniclus* | 20.57 | 0.68 |
| *Emberiza spodocephala* | 18.00 | 0.96 |
| *Euneornis campestris* | 16.20 | 0.73 |
| *Euphagus carolinus* | 55.70 | 1.56 |
| *Euphagus cyanocephalus* | 68.50 | 1.49 |
| *Euphonia jamaica* | 16.10 | 0.67 |
| *Fringilla coelebs* | 24.26 | 0.77 |
| *Fringilla montifringilla* | 24.94 | 0.78 |
| *Gnorimopsar chopi* | 79.50 | 1.86 |
| *Gymnostinops montezuma* | 376.50 | 6.10 |
| *Habia fuscicauda* | 39.70 | 1.39 |
| *Helmitheros vermivorus* | 14.20 | 0.61 |
| *Hemithraupis guira* | 12.00 | 0.59 |
| *Himatione sanguinea* | 14.80 | 0.69 |
| *Icteria virens* | 24.90 | 0.94 |
| *Icterus galbula* | 33.30 | 1.17 |
| *Icterus spurius* | 20.40 | 0.89 |
| *Junco hyemalis* | 19.00 | 0.86 |
| *Leucosticte arctoa* | 25.60 | 1.36 |
| *Limnothlypis swainsonii* | 14.00 | 0.69 |
| *Loxia curvirostra* | 34.30 | 1.47 |
| *Loxia leucoptera* | 31.80 | 1.43 |
| *Loxigilla violacea* | 28.00 | 1.20 |
| *Loxipasser anoxanthus* | 11.30 | 0.59 |
| *Melopyrrha nigra* | 10.90 | 0.87 |
| *Melospiza georgiana* | 17.60 | 0.84 |
| *Melospiza melodia* | 20.50 | 0.96 |
| *Mitrospingus cassinii* | 40.40 | 1.36 |
| *Mniotilta varia* | 12.00 | 0.46 |
| *Molothrus ater* | 41.70 | 1.19 |
| *Molothrus badius* | 44.50 | 1.53 |
| *Myioborus pictus* | 9.80 | 0.40 |
| *Nesospingus speculiferus* | 36.20 | 1.48 |
| *Paroaria coronata* | 43.00 | 1.27 |
| *Passerculus sandwichensis* | 20.10 | 0.70 |
| *Passerina cyanea* | 12.20 | 0.67 |
| *Phaenocophilus palmarum* | 29.20 | 1.17 |
| *Pheucticus ludovicianus* | 45.60 | 1.37 |
| *Pinicola enucleator* | 65.70 | 1.64 |
| *Pipilo erythrophthalmus* | 39.20 | 1.41 |
| *Protonotaria citrea* | 15.50 | 0.61 |
| *Pseudoleistes virescens* | 70.40 | 2.08 |
| *Pyrrhula Pyrrhula* | 21.80 | 0.97 |
| *Quiscalus major* | 158.80 | 2.88 |
| *Quiscalus mexicanus* | 168.70 | 3.07 |
| *Quiscalus quiscula* | 110.20 | 2.68 |
| *Rhodinocichla rosea* | 46.70 | 1.40 |
| *Rhodospingus cruentus* | 10.80 | 0.54 |
| *Saltator maximus* | 46.60 | 1.44 |
| *Scaphidura oryzivora* | 190.50 | 3.25 |
| *Schistochlamys melanopis* | 33.00 | 1.26 |
| *Seiurus aurocapilla* | 22.10 | 0.73 |
| *Serinus burtoni* | 29.60 | 1.21 |
| *Serinus canaria* | 8.40 | 0.45 |
| *Serinus flaviventris* | 16.30 | 0.75 |
| *Serinus serinus* | 11.20 | 0.65 |
| *Setophaga ruticilla* | 7.80 | 0.37 |
| *Sicalis flaveola* | 19.70 | 0.73 |
| *Spindalis zena* | 42.50 | 1.36 |
| *Spiza americana* | 27.00 | 0.94 |
| *Spizella arborea* | 12.40 | 0.77 |
| *Sporophila americana* | 10.70 | 0.62 |
| *Sturnella magna* | 89.00 | 2.30 |
| *Sturnella neglecta* | 103.50 | 2.01 |
| *Tachyphonus delattrii* | 16.80 | 0.77 |
| *Tangara cyanicollis* | 17.00 | 0.66 |
| *Tangara icterocephala* | 15.00 | 0.59 |
| *Thraupis episcopus* | 31.10 | 1.15 |
| *Tiaris olivacea* | 8.50 | 0.50 |
| *Vermivora peregrina* | 9.50 | 0.45 |
| *Vermivora pinus* | 8.40 | 0.42 |
| *Volatinia jacarina* | 12.50 | 0.51 |
| *Xanthocephalus xanthocephalus* | 76.60 | 1.71 |
| *Zonotrichia albicollis* | 27.40 | 1.09 |
| *Zonotrichia capensis* | 20.30 | 0.80 |
| *Zonotrichia leucophrys* | 26.40 | 1.00 |
| *Anumbius annumbi* | 41.50 | 1.22 |
| *Aphrastura spinicauda* | 11.50 | 0.84 |
| *Automolus infuscatus* | 33.10 | 1.17 |
| *Automolus ochrolaemus* | 40.20 | 1.18 |
| *Campylorhamphus pusillus* | 43.30 | 1.39 |
| *Certhiaxis cinnamomea* | 14.80 | 0.71 |
| *Cinclodes fuscus* | 30.45 | 0.93 |
| *Cinclodes patagonicus* | 53.20 | 1.28 |
| *Coryphistera alaudina* | 34.30 | 0.97 |
| *Dendrocincla fuliginosa* | 39.40 | 1.10 |
| *Dendrocolaptes certhia* | 66.70 | 1.80 |
| *Furnarius rufus* | 63.70 | 1.35 |
| *Glyphorhynchus spirurus* | 14.80 | 0.64 |
| *Lepidocolaptes affinis* | 29.00 | 1.17 |
| *Lepidocolaptes souleyetii* | 25.70 | 1.08 |
| *Phacellodomus ruber* | 39.30 | 1.15 |
| *Phleocryptes melanops* | 14.20 | 0.71 |
| *Sclerurus mexicanus* | 21.10 | 0.82 |
| *Sittasomus griseicapillus* | 14.00 | 0.57 |
| *Xenops minutus* | 11.70 | 0.51 |
| *Xiphorhynchus guttatus* | 46.40 | 1.55 |
| *Xiphorhynchus picus* | 34.60 | 1.35 |
| *Delichon urbica* | 14.50 | 0.50 |
| *Hirundo rustica* | 17.54 | 0.58 |
| *Notiochelidon cyanoleuca* | 9.70 | 0.45 |
| *Progne subis* | 50.70 | 1.07 |
| *Riparia riparia* | 13.80 | 0.42 |
| *Stelgidopteryx ruficollis* | 15.20 | 0.55 |
| *Tachycineta bicolor* | 20.10 | 0.57 |
| *Tachycineta thalassina* | 15.10 | 0.48 |
| *Chloropsis hardwickii* | 23.75 | 0.76 |
| *Chloropsis palawanensis* | 30.50 | 1.09 |
| *Irena puella* | 58.37 | 1.12 |
| *Lanius collaris* | 35.85 | 1.11 |
| *Lanius collurio* | 29.90 | 0.99 |
| *Lanius excubitor* | 59.63 | 1.48 |
| *Lanius ludovicianus* | 47.40 | 1.59 |
| *Lanius senator* | 27.80 | 1.11 |
| *Amytornis goyderi* | 16.70 | 0.81 |
| *Malurus cyaneus* | 8.30 | 0.49 |
| *Malurus elegans* | 10.10 | 0.59 |
| *Malurus lamberti* | 9.20 | 0.48 |
| *Malurus leucopterus* | 8.00 | 0.40 |
| *Malurus melanocephalus* | 7.00 | 0.47 |
| *Malurus pulcherrimus* | 9.80 | 0.44 |
| *Malurus splendens* | 11.40 | 0.47 |
| *Toxorhamphus iliolophum* | 12.90 | 0.50 |
| *Toxorhamphus poliopterus* | 11.40 | 0.45 |
| *Acanthagenys rufogularis* | 50.20 | 1.20 |
| *Acanthorynchus tenuirostris* | 11.20 | 0.50 |
| *Anthochaera carunculata* | 108.50 | 2.28 |
| *Anthochaera chrysoptera* | 66.30 | 1.76 |
| *Ashbyia lovensis* | 17.50 | 0.69 |
| *Conopophila rufogularis* | 10.80 | 0.46 |
| *Entomyzon cyanotis* | 106.50 | 2.31 |
| *Epthianura aurifrons* | 10.30 | 0.45 |
| *Epthianura tricolor* | 10.70 | 0.46 |
| *Lichenostomus chrysops* | 17.50 | 0.75 |
| *Lichenostomus flavescens* | 12.60 | 0.54 |
| *Lichenostomus flavus* | 21.10 | 0.74 |
| *Lichenostomus keartlandi* | 15.30 | 0.71 |
| *Lichenostomus leucotis* | 22.10 | 0.92 |
| *Lichenostomus melanops* | 19.80 | 0.95 |
| *Lichenostomus ornatus* | 19.80 | 0.78 |
| *Lichenostomus penicillatus* | 19.20 | 0.86 |
| *Lichenostomus virescens* | 33.30 | 0.95 |
| *Lichmera indistincta* | 11.40 | 0.53 |
| *Manorina flavigula* | 59.80 | 1.42 |
| *Manorina melanocephala* | 60.90 | 1.93 |
| *Manorina melanophrys* | 25.50 | 1.18 |
| *Meliphaga lewinii* | 36.20 | 1.21 |
| *Meliphaga notata* | 26.40 | 0.90 |
| *Melithreptus albogularis* | 11.10 | 0.65 |
| *Melithreptus brevirostris* | 14.30 | 0.70 |
| *Melithreptus lunatus* | 14.10 | 0.62 |
| *Myzomela obscura* | 11.90 | 0.53 |
| *Myzomela sanguinolenta* | 7.80 | 0.35 |
| *Philemon buceroides* | 121.00 | 2.23 |
| *Philemon corniculatus* | 105.80 | 2.05 |
| *Phylidonyris albifrons* | 17.10 | 0.75 |
| *Phylidonyris melanops* | 18.30 | 0.64 |
| *Phylidonyris novaehollandiae* | 19.40 | 0.93 |
| *Ramsayornis modestus* | 10.60 | 0.53 |
| *Xanthotis flaviventer* | 33.60 | 1.09 |
| *Menura novaehollandiae* | 644.40 | 11.11 |
| *Alethe diademata* | 33.60 | 1.08 |
| *Brachypteryx montana* | 18.00 | 0.86 |
| *Catharus fuscescens* | 41.50 | 0.95 |
| *Catharus minimus* | 32.80 | 0.80 |
| *Catharus ustulatus* | 30.80 | 0.82 |
| *Cercotrichas coryphaeus* | 23.10 | 0.75 |
| *Cichlerminia lherminieri* | 100.00 | 2.28 |
| *Copsychus malabaricus* | 30.00 | 0.90 |
| *Copsychus sauIaris* | 33.20 | 1.14 |
| *Cossypha caffra* | 28.50 | 1.06 |
| *Culicicapa helianthea* | 7.50 | 0.32 |
| *Enicurus scouleri* | 14.00 | 0.79 |
| *Erithacus rubecula* | 16.75 | 0.66 |
| *Hylocichla mustelinus* | 47.40 | 1.30 |
| *Monticola saxatilis* | 48.50 | 1.34 |
| *Myadestes genibarbis* | 27.10 | 0.95 |
| *Myadestes townsendi* | 32.50 | 1.05 |
| *Phoenicurus ochruros* | 16.50 | 0.70 |
| *Rhinomyias gularis* | 18.00 | 1.09 |
| *Saxicola torquata* | 15.30 | 0.63 |
| *Sialia mexicana* | 26.40 | 0.87 |
| *Sigelus silens* | 28.30 | 1.04 |
| *Turdus merula* | 98.17 | 1.92 |
| *Turdus migratorius* | 80.20 | 1.70 |
| *Turdus philomelos* | 69.64 | 1.59 |
| *Zoothera lunulata* | 104.00 | 2.24 |
| *Aethopyga nipalensis* | 6.00 | 0.34 |
| *Anthreptes malacensis* | 11.90 | 0.60 |
| *Arachnothera longirostra* | 11.70 | 0.54 |
| *Dicaeum aeruginosum* | 11.80 | 0.52 |
| *Dicaeum hirundinaceum* | 8.00 | 0.38 |
| *Nectarinia jugularis* | 8.70 | 0.41 |
| *Nectarinia verticalis* | 14.00 | 0.52 |
| *Prionochilus plateri* | 7.90 | 0.41 |
| *Orthonyx temminckii* | 62.30 | 1.82 |
| *Acanthiza chrysorrhoa* | 10.00 | 0.45 |
| *Acanthiza lineata* | 6.40 | 0.53 |
| *Acanthiza pusilla* | 6.00 | 0.47 |
| *Acanthiza reguloides* | 7.50 | 0.40 |
| *Acanthiza uropygialis* | 6.50 | 0.37 |
| *Aphelocephala leucopsis* | 12.90 | 0.56 |
| *Dasyornis broadbenti* | 83.30 | 1.95 |
| *Pardalotus punctatus* | 9.20 | 0.41 |
| *Pardalotus striatus* | 11.60 | 0.54 |
| *Sericornis citreogularis* | 16.60 | 0.82 |
| *Sericornis magnirostris* | 8.50 | 0.62 |
| *Baeolophus bicolor* | 21.60 | 1.05 |
| *Parus ater* | 8.00 | 0.51 |
| *Parus caeruleus* | 10.79 | 0.65 |
| *Parus cristatus* | 10.20 | 0.70 |
| *Parus cyanus* | 9.95 | 0.66 |
| *Parus major* | 17.02 | 0.85 |
| *Parus montanus* | 10.20 | 0.79 |
| *Parus palustris* | 10.60 | 0.58 |
| *Parus venustulus* | 10.54 | 0.53 |
| *Poecile atricapillus* | 12.00 | 0.79 |
| *Poecile carolinensis* | 10.20 | 0.62 |
| *Poecile gambeli* | 11.30 | 0.78 |
| *Poecile hudsonicus* | 11.00 | 0.74 |
| *Amadina fasciata* | 15.40 | 0.61 |
| *Amandava amandava* | 9.60 | 0.39 |
| *Anthus berthelotii* | 15.99 | 0.52 |
| *Anthus campestris* | 23.00 | 0.48 |
| *Anthus novaeseelandiae* | 27.90 | 0.87 |
| *Anthus pratensis* | 18.40 | 0.53 |
| *Anthus trivialis* | 18.40 | 0.62 |
| *Bubalornis albirostris* | 64.50 | 2.21 |
| *Chloebia gouldiae* | 10.00 | 0.55 |
| *Erythrura trichroa* | 14.40 | 0.60 |
| *Estrilda astrild* | 7.50 | 0.35 |
| *Estrilda troglodytes* | 6.10 | 0.36 |
| *Euplectes orix* | 16.30 | 0.79 |
| *Foudia madagascariensis* | 16.00 | 0.78 |
| *Lagonosticta senegala* | 8.30 | 0.40 |
| *Lonchura bicolor* | 9.69 | 0.44 |
| *Lonchura cucullata* | 9.20 | 0.39 |
| *Lonchura flaviprymna* | 11.68 | 0.43 |
| *Lonchura malacca* | 12.60 | 0.62 |
| *Lonchura pallida* | 10.65 | 0.52 |
| *Lonchura spectabilis* | 7.90 | 0.49 |
| *Lonchura striata* | 12.30 | 0.48 |
| *Montifringilla nivalis* | 36.90 | 1.09 |
| *Motacilla alba* | 18.96 | 0.58 |
| *Motacilla flava* | 14.40 | 0.57 |
| *Neochmia phaethon* | 10.00 | 0.52 |
| *Neochmia temporalis* | 10.90 | 0.54 |
| *Padda oryzivora* | 24.50 | 0.88 |
| *Passer domesticus* | 28.49 | 0.92 |
| *Passer griseus* | 23.90 | 0.97 |
| *Passer hispaniolensis* | 27.13 | 0.93 |
| *Passer montanus* | 21.38 | 0.79 |
| *Passer rutilans* | 18.35 | 0.68 |
| *Plocepasser mahali* | 43.30 | 1.27 |
| *Ploceus cucullatus* | 40.90 | 1.42 |
| *Poephila acuticauda* | 14.00 | 0.53 |
| *Poephila cincta* | 16.10 | 0.49 |
| *Poephila personata* | 11.80 | 0.52 |
| *Prunella modularis* | 20.41 | 0.71 |
| *Pyrenestes sanguineous* | 14.27 | 0.97 |
| *Pytilia melba* | 13.50 | 0.59 |
| *Pytilia phoenicoptera* | 14.50 | 0.37 |
| *Spermophaga haematina* | 16.87 | 0.91 |
| *Staganopleura guttata* | 19.00 | 0.65 |
| *Taeniopygia bichenovii* | 10.50 | 0.41 |
| *Taeniopygia guttata* | 12.00 | 0.46 |
| *Uraeginthus bengalus* | 10.30 | 0.45 |
| *Vidua paradisaea* | 22.20 | 0.66 |
| *Drymodes brunneopygia* | 38.70 | 0.98 |
| *Eopsaltria australis* | 19.60 | 0.89 |
| *Eopsaltria griseogularis* | 21.10 | 0.86 |
| *Melanodryas cucullata* | 24.30 | 0.83 |
| *Microeca fascinans* | 11.40 | 0.56 |
| *Microeca flavigaster* | 12.70 | 0.53 |
| *Pachycephalopsis poliosoma* | 38.30 | 1.10 |
| *Petroica goodenovii* | 8.70 | 0.38 |
| *Petroica multicolor* | 9.60 | 0.54 |
| *Tragellasia leucops* | 16.00 | 0.61 |
| *Pitta brachyura* | 55.50 | 1.44 |
| *Pitta erythrogaster* | 48.35 | 0.95 |
| *Pitta guajana* | 69.51 | 1.42 |
| *Pitta sordida* | 51.51 | 1.25 |
| *Pitta versicolor* | 107.70 | 1.94 |
| *Pomatostomus halli* | 37.50 | 1.66 |
| *Pomatostomus isidorei* | 64.00 | 2.26 |
| *Pomatostomus ruficeps* | 56.00 | 2.43 |
| *Pomatostomus superciliosus* | 35.00 | 1.57 |
| *Pomatostomus temporalis* | 75.00 | 1.97 |
| *Ailuroedus crassirostris* | 204.00 | 4.15 |
| *Amblyornis macgregoriae* | 123.50 | 3.71 |
| *Chlamydera nuchalis* | 199.50 | 5.19 |
| *Ptilonorhynchus violaceus* | 217.00 | 4.89 |
| *Sericulus chrysocephalus* | 100.50 | 3.46 |
| *Alophoixus pallidus* | 46.00 | 1.28 |
| *Chlorocichla flavicollis* | 45.30 | 1.53 |
| *Nicator chloris* | 38.20 | 1.33 |
| *Pycnonotus barbatus* | 35.90 | 1.17 |
| *Pycnonotus jocosus* | 27.40 | 0.96 |
| *Regulus regulus* | 5.70 | 0.38 |
| *Rhinocrypta lanceolata* | 63.60 | 1.45 |
| *Sitta canadensis* | 10.50 | 0.59 |
| *Sitta carolinensis* | 17.80 | 0.89 |
| *Sitta europaea* | 23.03 | 1.11 |
| *Sitta pygmaea* | 10.40 | 0.57 |
| *Acridotheres tristis* | 115.60 | 2.57 |
| *Aplonis metallica* | 61.00 | 1.64 |
| *Dumetella carolinensis* | 37.80 | 1.18 |
| *Gracula religiosa* | 192.00 | 3.81 |
| *Lamprotornis purpureus* | 115.50 | 2.20 |
| *Lamprotornis superbus* | 54.15 | 1.78 |
| *Margarops fuscatus* | 109.40 | 2.43 |
| *Margarops fuscus* | 70.80 | 1.60 |
| *Melanoptila glabirostris* | 35.00 | 1.24 |
| *Mimus polyglottos* | 45.20 | 1.43 |
| *Poeoptera lugubris* | 38.00 | 1.03 |
| *Sarcops calvus* | 142.00 | 2.90 |
| *Sturnus roseus* | 66.50 | 1.48 |
| *Sturnus vulgaris* | 109.40 | 2.24 |
| *Toxostoma curvirostre* | 78.40 | 2.21 |
| *Abroscopus albogularis* | 5.00 | 0.33 |
| *Acrocephalus arundinaceus* | 29.90 | 0.94 |
| *Acrocephalus melanopogon* | 11.27 | 0.41 |
| *Acrocephalus orientalis* | 23.98 | 0.87 |
| *Acrocephalus palustris* | 11.70 | 0.39 |
| *Acrocephalus schoenobaenus* | 10.80 | 0.47 |
| *Acrocephalus scirpaceus* | 12.30 | 0.48 |
| *Chaemaea fasciata* | 14.70 | 0.80 |
| *Cincloramphus cruralis* | 43.00 | 1.16 |
| *Cincloramphus mathewsi* | 25.00 | 0.93 |
| *Garrulax leucolophus* | 123.50 | 2.55 |
| *Heterophasia melanoleuca* | 32.60 | 1.20 |
| *Hippolais icterina* | 12.74 | 0.54 |
| *Illadopsis fulvescens* | 24.40 | 1.23 |
| *Leiothrix argentauris* | 26.20 | 0.97 |
| *Leiothrix lutea* | 21.80 | 0.95 |
| *Locustella fluviatilis* | 18.80 | 0.45 |
| *Locustella luscinioides* | 15.00 | 0.57 |
| *Macronous gularis* | 13.50 | 0.67 |
| *Megalurus palustris* | 33.30 | 1.18 |
| *Minla ignotincta* | 14.30 | 0.74 |
| *Phylloscopus bonelli* | 7.40 | 0.35 |
| *Phylloscopus collybita* | 8.02 | 0.38 |
| *Phylloscopus sibiliatrix* | 7.20 | 0.37 |
| *Phylloscopus trochilus* | 9.92 | 0.31 |
| *Pomatorhinus ruficollis* | 31.70 | 1.59 |
| *Pteruthius flaviscapis* | 39.00 | 1.55 |
| *Stachyris whiteheadi* | 19.10 | 0.85 |
| *Sylvia atricapilla* | 19.37 | 0.67 |
| *Sylvia borin* | 18.22 | 0.62 |
| *Sylvia communis* | 14.09 | 0.56 |
| *Sylvia curruca* | 12.82 | 0.53 |
| *Sylvia hortensis* | 22.25 | 0.79 |
| *Sylvia melanocephala* | 10.98 | 0.53 |
| *Sylvia nisoria* | 22.80 | 0.71 |
| *Yuhina diademata* | 12.00 | 0.80 |
| *Cercomacra tyrannina* | 16.60 | 0.77 |
| *Dystithamnus mentalis* | 11.20 | 0.73 |
| *Formicivora grisea* | 9.30 | 0.47 |
| *Gymnocichla nudiceps* | 32.80 | 1.10 |
| *Gymnopithys leucaspis* | 31.10 | 0.79 |
| *Hylophylax naevia* | 12.50 | 0.61 |
| *Hylophylax poecilonota* | 16.60 | 0.66 |
| *Hypocnemis cantator* | 10.00 | 0.65 |
| *Myrmotherula axillaris* | 7.40 | 0.40 |
| *Myrmotherula fulviventris* | 10.20 | 0.50 |
| *Phaenostictus mcleannani* | 51.10 | 1.11 |
| *Pithys albifrons* | 19.70 | 0.64 |
| *Pyriglena leuconota* | 32.80 | 0.96 |
| *Sakesphorus luctuosus* | 31.00 | 1.19 |
| *Taraba major* | 67.50 | 1.57 |
| *Thamnomanes caesius* | 14.20 | 0.63 |
| *Thamnophilus caerulescens* | 20.00 | 1.02 |
| *Thamnophilus punctatus* | 22.40 | 0.97 |
| *Attila spadiceus* | 33.20 | 1.22 |
| *Camptostoma obsoletum* | 8.00 | 0.37 |
| *Chiroxiphia caudata* | 25.60 | 0.83 |
| *Chiroxiphia linearis* | 18.50 | 0.70 |
| *Cnemotriccus fuscatus* | 13.50 | 0.48 |
| *Colonia colonus* | 16.80 | 0.49 |
| *Contopus latirostris* | 10.60 | 0.39 |
| *Contopus virens* | 14.20 | 0.47 |
| *Elaenia frantzii* | 19.40 | 0.63 |
| *Elaenia martinica* | 18.60 | 0.67 |
| *Empidonax minimus* | 10.50 | 0.38 |
| *Empidonax virescens* | 12.90 | 0.45 |
| *Gymnoderus foetidus* | 275.00 | 3.66 |
| *Hemitriccus margaritaceiventer* | 7.70 | 0.47 |
| *Hymenops perspicillatus* | 23.10 | 0.79 |
| *Legatus leucophaius* | 24.40 | 0.68 |
| *Lessonia rufa* | 14.48 | 0.44 |
| *Lipaugus vociferans* | 82.20 | 1.69 |
| *Manacus candei* | 17.60 | 0.59 |
| *Manacus manacus* | 19.00 | 0.61 |
| *Manacus vitellinus* | 18.20 | 0.64 |
| *Mecocerculus leucophrys* | 13.90 | 0.50 |
| *Mionectes oligeneus* | 13.20 | 0.47 |
| *Mitrephanes phaeocercus* | 8.60 | 0.33 |
| *Muscisaxicola alpina* | 22.80 | 0.81 |
| *Myiarchus cinerascens* | 28.80 | 0.80 |
| *Myiarchus stolidus* | 19.30 | 0.67 |
| *Myiarchus tuberculifer* | 18.70 | 0.68 |
| *Myiarchus tyrannulus* | 35.30 | 1.02 |
| *Myiobius barbatus* | 11.90 | 0.36 |
| *Myiodynastes maculatus* | 45.90 | 1.17 |
| *Myiopagis cotta* | 13.00 | 0.44 |
| *Myiophobus fasciatus* | 9.90 | 0.38 |
| *Myiozetetes similis* | 27.80 | 0.85 |
| *Onychorhynchus coronatus* | 14.00 | 0.49 |
| *Pachyramphus cinnamomeus* | 20.30 | 0.91 |
| *Pachyramphus polychopterus* | 20.30 | 0.77 |
| *Perissocephalus tricolor* | 339.50 | 4.87 |
| *Phaeomyias murina* | 10.00 | 0.38 |
| *Phytotoma rara* | 40.00 | 1.20 |
| *Pipra erythrocephala* | 13.60 | 0.51 |
| *Pipra fasciicauda* | 14.30 | 0.64 |
| *Pipra mentalis* | 15.20 | 0.61 |
| *Pitangus sulphuratus* | 70.20 | 1.32 |
| *Platyrinchus concrominus* | 9.70 | 0.51 |
| *Pseudocolopteryx flaviventris* | 7.40 | 0.35 |
| *Pseudotriccus pelzelni* | 10.90 | 0.66 |
| *Pyrocephalus rubinus* | 12.70 | 0.48 |
| *Pyroderus scutatus* | 357.00 | 4.45 |
| *Querula purpurata* | 101.80 | 2.21 |
| *Rupicola peruviana* | 243.50 | 3.48 |
| *Sayornis nigricans* | 18.60 | 0.51 |
| *Sayornis phoebe* | 18.30 | 0.61 |
| *Sayornis saya* | 21.70 | 0.63 |
| *Schiffornis turdinus* | 30.80 | 1.11 |
| *Tityra cayana* | 73.90 | 1.67 |
| *Tityra semifasciata* | 79.30 | 1.95 |
| *Todirostrum cinereum* | 6.40 | 0.32 |
| *Tolmomyias sulphurescens* | 14.90 | 0.53 |
| *Tyrannus savanna* | 28.60 | 0.76 |
| *Tyrannus tyrannus* | 39.50 | 0.98 |
| *Xolmis irupero* | 29.80 | 0.85 |
| *Xolmis pyrope* | 42.80 | 1.33 |
| *Cyclarhis gujanensis* | 28.80 | 1.31 |
| *Vireo altiloquus* | 19.50 | 0.78 |
| *Vireo olivaceus* | 20.30 | 0.63 |
| *Vireo philadelphicus* | 12.20 | 0.48 |
| *Zosterops japonicus* | 10.20 | 0.56 |
| *Zosterops lateralis* | 10.6 | 0.49 |

**Table S1c: 33 insectivore species** [**^1^**](#_ENREF_1)

| **Species** | **Body (gram)** | **Brain (gram)** |
| --- | --- | --- |
| *Solenodon paradoxus* | 900 | 4.67 |
| *Tenrec Ecaudatus* | 832 | 2.57 |
| *Setifer setosus* | 248 | 1.51 |
| *Hemicentetes semispinosus* | 110 | 0.83 |
| *Echinops telfairi* | 87.5 | 0.62 |
| *Oryzorictes talpoides* | 44.2 | 0.58 |
| *Microgale cowani* | 15.2 | 0.42 |
| *Nesogale cowani* | 32.6 | 0.56 |
| *Nesogale talazaci* | 50.4 | 0.79 |
| *Limnogale mergulus* | 92 | 1.15 |
| *Potamogale velox* | 660 | 4.1 |
| *Erinaceus europaeus* | 8.60E+02 | 3.35 |
| *Sorex minutus* | 5.30E+00 | 0.11 |
| *Sorex araneus* | 1.03E+01 | 0.2 |
| *Blarina brevicaudata* | 18.5 | 0.37 |
| *Neomys fodiens* | 15.2 | 0.32 |
| *Sylvisorex megalura* | 5.3 | 0.15 |
| *Sylvisorex lunaris* | 18.5 | 0.34 |
| *Suncus murinus* | 35.5 | 0.38 |
| *Crocidura hildegardae* | 10.6 | 0.22 |
| *Crocidura russula* | 11 | 0.19 |
| *Crocidura niobe* | 11.5 | 0.28 |
| *Crocidura jacksoni* | 12.6 | 0.25 |
| *Crocidura occidentalis* | 28 | 0.44 |
| *Crocidura giffardi* | 82 | 0.55 |
| *Galemys pyrenaicus* | 57.5 | 1.33 |
| *Desmana moschata* | 440 | 4 |
| *Talpa europaea* | 76 | 1.02 |
| *Scalopus aquaticus* | 40 | 1.16 |
| *Chrysochloris asiatica* | 49 | 0.7 |
| *Chlorotalpa stuhlmanni* | 40 | 0.74 |
| *Elephantulus fuscipes* | 57 | 1.33 |
| *Rhynchocyon stuhlmanni* | 490 | 6.1 |

**Table S1d: 18 archaic mammalian species**[**^1^**](#_ENREF_1)

| **Species** | **Body (gram)** | **Brain (gram)** |
| --- | --- | --- |
| *Arctocyon primaevus* | 86000 | 38 |
| *Arctocyonides arenae* | 11000 | 8.3 |
| *Pleuraspidotherium* | 3300 | 6 |
| *Phenacodus primaevus* | 56000 | 31 |
| *Meniscotherium* | 6200 | 15 |
| *Hyopsodus miticulus* | 630 | 3.2 |
| *Pantolamba bathmodon* | 30000 | 19 |
| *Leptolambda schmidti* | 205000 | 69 |
| *Barylambda* | 620000 | 102 |
| *Coryphodon hamatus* | 270000 | 93 |
| *Coryphodon elephantopus* | 540000 | 90 |
| *Uintatherium anceps* | 1.40E+06 | 300 |
| *Uintatherium anceps* | 1.40E+06 | 250 |
| *Tetheopsis ingens* | 2.50E+06 | 350 |
| *Thinocyon Velox* | 800 | 5.7 |
| *Cynohyaenodon cayluxi* | 3000 | 8.3 |
| *Pterodon dasyuroides* | 42000 | 62 |
| *Hyaenodon horridus* | 56000 | 85 |

**Table S1e: 7 mesozoic mammals**[**^1^**](#_ENREF_1)

| **Species** | **Body (gram)** | **Brain (gram)** |
| --- | --- | --- |
| *Triconodon mordax* | 100 | 0.73 |
| *Ptilodus montanus* | 200 | 1 |
| *Neurogymnurus cayluxi* | 200 | 1.6 |
| *Setifer setosus* | 220 | 1.5 |
| *Echinosorex gymnurus* | 850 | 3.5 |
| *Didelphis marsupialis* | 5000 | 7.6 |
| *Rattus norvegicus* | 300 | 2 |

**Table S1a: 17 dinosaur species**[**^1^**](#_ENREF_1)

| **Species** | **Body (gram)** | **Brain (gram)** |
| --- | --- | --- |
| *S BrachiosaurusA* | 7.83E+07 | 186 |
| *S DiplodocusA* | 1.90E+07 | 57 |
| *A Euoplocephalus* | 1.90E+06 | 41 |
| *G StegosaurusA* | 3.10E+06 | 22.5 |
| *G Kentrosaurus* | 780000 | 24 |
| *C Protoceratops* | 200000 | 28 |
| *C TriceratopsA* | 9.00E+06 | 72.2 |
| *O Iguanodon* | 2.10E+06 | 125 |
| *O Camptosaurus* | 400000 | 23 |
| *O Anatosaurus* | 3.40E+06 | 150 |
| *T AllosaurusA* | 2.30E+06 | 168 |
| *T TyrannosaurusA* | 7.40E+06 | 202 |
| *E Edmontosaurus* | 6.20E+06 | 200 |
| *T DromicieomimusA* | 175000 | 87.85 |
| *T Troodon* | 45000 | 18 |
| *B ArchaeopteryxA* | 400 | 1.47 |
| *Old Troodon* | 45000 | 45 |

**Table S1g: 110 fish species**[**^6^**](#_ENREF_6)

| **Species** | **Body (gram)** | **Brain (gram)** |
| --- | --- | --- |
| *Abudefduf* | 60 | 0.209 |
| *Abudefduf* | 200 | 0.304 |
| *Acanthurus* | 140 | 0.489 |
| *Adontosternarchus* | 7 | 0.117 |
| *Acentrogobius* | 3.6 | 0.02 |
| *Amblycirrhitus* | 3.3 | 0.033 |
| *Anampses* | 2.5 | 0.0267 |
| *Amphiprion* | 32 | 0.102 |
| *Amoya* | 0.7 | 0.01 |
| *Aphareus* | 5200 | 2.875 |
| *Anyperodon* | 60 | 0.24 |
| *Antennablennius* | 1.3 | 0.0138 |
| *Apogon* | 2.5 | 0.027 |
| *Apogon* | 17.2 | 0.075 |
| *Aprion* | 1630 | 1.972 |
| *Atule* | 250 | 0.661 |
| *Awaous* | 3.6 | 0.0145 |
| *Balistapus* | 470 | 0.527 |
| *Bathygobius* | 10.8 | 0.0283 |
| *Calotomus* | 157 | 0.307 |
| *Carangoides* | 6200 | 2.934 |
| *Ctenochaetus* | 40 | 0.247 |
| *Gerres* | 29 | 0.158 |
| *Gobiopsis* | 0.2 | 0.0012 |
| *Halichoeres* | 15.7 | 0.0926 |
| *Katsuwonus* | 6500 | 4.659 |
| *Labroides* | 5.7 | 0.0642 |
| *Kyphosus* | 105 | 0.274 |
| *Lethrinus* | 500 | 1.663 |
| *Lethrinus* | 350 | 1.007 |
| *Lethrinus* | 1680 | 2.369 |
| *Lophogobius* | 8.9 | 0.0167 |
| *Lutjanus* | 970 | 1.75 |
| *Lutjanus* | 155 | 0.832 |
| *Macolor* | 2620 | 2.724 |
| *Malacanthus* | 6.4 | 0.0573 |
| *Macrodontogobius* | 0.2 | 0.0045 |
| *Meiacanthus* | 0.4 | 0.0076 |
| *Megalaspis* | 140 | 0.627 |
| *Naso* | 610 | 1.122 |
| *Naso* | 280 | 0.82 |
| *Nematogobius* | 0.6 | 0.0071 |
| *Neoniphon* | 169 | 0.612 |
| *Neoniphon* | 37 | 0.26 |
| *Oplopomus* | 0.3 | 0.0067 |
| *hyperoplus immaculatus* | 110 | 0.155 |
| *Hyperoplus* | 19 | 0.05 |
| *lepomis gibbosus* | 29 | 0.14 |
| *lepidion eques* | 80 | 0.3 |
| *Lutjanus* | 7400 | 2.992 |
| *Lumpenus* | 30 | 0.08 |
| *Lipophrys* | 4.2 | 0.037 |
| *Merluccius* | 2999 | 0.993 |
| *merlangius merlangus* | 800 | 0.77 |
| *Mugil* | 750 | 0.506 |
| *Molva* | 8995 | 2.3 |
| *Mola* | 4600 | 0.922 |
| *Nerophis* | 1.3 | 0.0034 |
| *Phycis* | 5998 | 1.513 |
| *Alosa* | 350 | 0.438 |
| *Alosa* | 84 | 0.234 |
| *Ammodytes* | 4.3 | 0.015 |
| *Amia* | 727 | 0.403 |
| *Belone* | 282 | 0.421 |
| *Brosme* | 600 | 0.622 |
| *Callionymus* | 8 | 0.0453 |
| *Ciliata* | 10 | 0.0469 |
| *Cottunculus* | 120 | 0.092 |
| *Gadus* | 610 | 0.759 |
| *Gymnogobius* | 1.5 | 0.01 |
| *Hyperlophus* | 1.7 | 0.016 |
| *Hypseleotris* | 0.8 | 0.006 |
| *Knipowitschia* | 0.5 | 0.0048 |
| *Lepidonotothen* | 300 | 0.338 |
| *Lepidotrigla* | 72 | 0.142 |
| *Lipophrys* | 5 | 0.044 |
| *Liza* | 51 | 0.149 |
| *Macrourus* | 115 | 0.405 |
| *Micromesistius* | 50 | 0.247 |
| *Odontobutis* | 20 | 0.03 |
| *Osmerus* | 74 | 0.116 |
| *Pollachius* | 9397 | 2.449 |
| *Pollachius* | 5998 | 2.218 |
| *Idiacanthus* | 6.3 | 0.0058 |
| *Latimeria* | 5850 | 0.2 |
| *Lampetra* | 174 | 0.015 |
| *Lophius* | 14400 | 0.621 |
| *Lophius* | 500 | 0.183 |
| *Myoxocephalus* | 80 | 0.12 |
| *Notacanthus* | 4500 | 0.812 |
| *Petromyzon* | 282 | 0.0175 |
| *Anarhichas* | 2900 | 0.272 |
| *Cyclopterus* | 925 | 0.192 |
| *Leptagonus* | 20 | 0.035 |
| *Myoxocephalus* | 80 | 0.121 |
| *Notothenia* | 1450 | 0.741 |
| *Anarhichas* | 2250 | 0.458 |
| *Malacosteus* | 24 | 0.016 |
| *Chauliodus* | 32 | 0.03 |
| *Gonostoma* | 60 | 0.051 |
| *Carcharhinus* | 36240 | 43.32 |
| *Carcharhinus* | 12000 | 20.76 |
| *Centrophorus* | 2820 | 4.31 |
| *Blackbelly* | 60 | 0.48 |
| *Ginglymostoma* | 45300 | 31.65 |
| *Galeocerdo* | 200000 | 107.5 |
| *Heterodontus* | 2930 | 4.3 |
| *Prionace* | 36100 | 21.21 |
| *Squaliolus* | 60 | 0.44 |
| *Rhizoprionodon* | 3750 | 7.18 |

**Table S1h: 71 reptilian species** [^7^](#_ENREF_7)

| **Species** | **Body (gram)** | **Brain (gram)** |
| --- | --- | --- |
| *Pyhllodactylus gerrophygus* | 0.68 | 0.018 |
| *Hemidactylus mabouia* | 2.5 | 0.033 |
| *Phelsuma cepediana* | 5 | 0.059 |
| *Tarentola mauritanica* | 7.8 | 0.07 |
| *Gekko gecko* | 54.8 | 0.198 |
| *Chalcides mionecton* | 6.4 | 0.03 |
| *Chalcides polylepis* | 7.9 | 0.037 |
| *Chalcides chalcides* | 18.8 | 0.0555 |
| *Chalcides ocellatus* | 32 | 0.09 |
| *Scincus scincus* | 34.1 | 0.116 |
| *Eumeces schneideri* | 51.7 | 0.1715 |
| *Psammodromus hispanicus* | 2.1 | 0.025 |
| *Zootoca vivipara* | 3.2 | 0.028 |
| *Lacerta muralis* | 4.2 | 0.046 |
| *Psammodromus algirus* | 4.3 | 0.045 |
| *Lacerta viridis* | 21.2 | 0.109 |
| *Lacerta lepida* | 70.8 | 0.224 |
| *Cordylus cordylus* | 56.5 | 0.175 |
| *Zonosaurus quadrilineatus* | 82.7 | 0.209 |
| *Zonosaurus maximus* | 386.4 | 0.565 |
| *Anguis fragilis* | 22 | 0.044 |
| *Ophisaurus apodus* | 498 | 0.342 |
| *Chalarodon madagascariensis* | 6.3 | 0.06 |
| *Anolis carolinensis* | 8.4 | 0.055 |
| *Anolis auratus* | 10.5 | 0.073 |
| *Liolaemus chiliensis* | 26 | 0.1035 |
| *Oplurus sebae* | 51 | 0.267 |
| *Iguana iguana* | 253.5 | 0.606 |
| *Amblyrhynchus cristatus* | 4190 | 1.44 |
| *Calotes cristatellus* | 1.7 | 0.029 |
| *Draco volans* | 4.5 | 0.058 |
| *Agama inermis* | 12.9 | 0.099 |
| *Calotes versicolor* | 14.6 | 0.097 |
| *Agama agama* | 29.3 | 0.173 |
| *Acanthosaura armata* | 43 | 0.182 |
| *Uromastix acanthinurus* | 164 | 0.335 |
| *Chamaeleo lateralis* | 10.9 | 0.061 |
| *Ameiva sp.* | 27.1 | 0.231 |
| *Callopistes trimaculatus* | 50.3 | 0.318 |
| *Tupinambis nigropunctatus* | 1034 | 1.563 |
| *Varanus griseus* | 254.2 | 0.722 |
| *Heloderma suspectum* | 514 | 0.729 |
| *Sphenodon punctatus* | 613 | 0.642 |
| *Boa constrictor* | 4460 | 0.649 |
| *Python molurus* | 6140 | 1.123 |
| *Natrix natrix* | 74.1 | 0.115 |
| *Thamnophis sirtalis* | 54.5 | 0.1 |
| *Natrix maura* | 86 | 0.095 |
| *Coronella girondica* | 117 | 0.088 |
| *Elaphe longissima* | 148.2 | 0.168 |
| *Coluber viridiflavus* | 285.1 | 0.209 |
| *Coluber constrictor* | 431 | 0.291 |
| *Zamenis viridus flavus* | 220 | 0.209 |
| *Naja melanoleuca* | 1770 | 0.646 |
| *Vipera aspis* | 68.7 | 0.1015 |
| *Cerastes vipera* | 62.1 | 0.076 |
| *Vipera berus* | 64.2 | 0.105 |
| *Agkistrodon piscivorous* | 728 | 0.64 |
| *Trogonophis wiegmanni* | 6.5 | 0.021 |
| *Chelydra serpentina* | 5125 | 0.98 |
| *Macroclemys temminckii* | 1848 | 1.01 |
| *Emys orbicularis* | 250 | 0.25 |
| *Pseudemys scripta* | 1418 | 0.738 |
| *Clemmys guttata* | 2163 | 1.36 |
| *Testudo graeca* | 267.5 | 0.318 |
| *Testudo hermanni* | 693.4 | 0.476 |
| *Chelonia mydas* | 114300 | 8.6 |
| *Caretta caretta* | 5443 | 2.7 |
| *Trionyx ferox* | 3253 | 2.5 |
| *Alligator mississippiensis* | 189000 | 12.64 |
| *Crocodylus acutus* | 122000 | 13.3 |

**Table S1i: 87 amphibian species** [^7^](#_ENREF_7)

| **Species** | **Body (gram)** | **Brain (gram)** |
| --- | --- | --- |
| *salamandra terrestris* | 12.2 | 0.0364 |
| *Salamandra atra* | 8.5 | 0.0273 |
| *Triturus alpestris* | 2.5 | 0.0142 |
| *Onychodactylus japonicus* | 5.1 | 0.0216 |
| *Hydromantes italicus* | 2.3 | 0.0194 |
| *Batrachoseps attenuatus* | 0.8 | 0.0035 |
| *Hyla versicolor* | 13.15 | 0.0423 |
| *Pseudacris triseriata* | 0.1 | 0.0023 |
| *Acris crepitans* | 0.3 | 0.0044 |
| *Agalychnis annae* | 6.04 | 0.065 |
| *Agalychnis callidryas* | 5.35 | 0.0614 |
| *Phyllomeduda bicolor* | 1.32 | 0.0201 |
| *Phyllomeduda sauvagii* | 12.4 | 0.0563 |
| *Hyla arborea* | 8.28 | 0.047 |
| *Hyla cinerea* | 6.27 | 0.0437 |
| *Hyla lineomaculata* | 1.82 | 0.0292 |
| *Hyla meridionalis* | 6.21 | 0.0392 |
| *Hyla pseudopuma* | 1.8 | 0.0305 |
| *Hyla raniceps* | 14.28 | 0.0752 |
| *Hyla rubra* | 6 | 0.0388 |
| *Hyla arborea savignyi* | 8.92 | 0.0548 |
| *Hyla uranochroa* | 0.9 | 0.0229 |
| *Phrynohyas venulosa* | 35.93 | 0.1132 |
| *Smilisca phaeota* | 2.84 | 0.0362 |
| *Smilisca sordida* | 4 | 0.0395 |
| *Platymantis vitiensis* | 0.3 | 0.0049 |
| *Rana sylvatica* | 0.37 | 0.0086 |
| *Rana pipiens* | 1.1 | 0.0204 |
| *Rana clamitans* | 96.3 | 0.1534 |
| *Rana catesbeiana* | 199.7 | 0.289 |
| *Rana esculenta* | 133.1 | 0.2074 |
| *Rana mascareniensis* | 7.25 | 0.04 |
| *Rana palmipes* | 68.4 | 0.1736 |
| *Rana ridibunda* | 31.7 | 0.1144 |
| *Rana temporaria* | 52.2 | 0.1122 |
| *Phrynobatrachus calcaratus* | 1.21 | 0.0124 |
| *Chiromantis xerampelina* | 18.08 | 0.0758 |
| *Xenopis laevis* | 32.37 | 0.0514 |
| *Rhinophrynus dorsalis* | 16.57 | 0.0329 |
| *Hemisus guineensis* | 18.84 | 0.0361 |
| *Alytes cisternasii* | 5.5 | 0.0338 |
| *Alytes obstetricans* | 6.9 | 0.0409 |
| *Bombina orientalis* | 6 | 0.0332 |
| *Bombina variegata* | 6.8 | 0.0339 |
| *Discoglossus pictus* | 22.1 | 0.0666 |
| *Pelobates cultripes* | 25.69 | 0.0809 |
| *Pelobates syriacus* | 28.77 | 0.1033 |
| *Pelodytes punctatus* | 15.5 | 0.045 |
| *Adelotus brevis* | 4.73 | 0.0224 |
| *Mixophyes fasciolatus* | 51.24 | 0.1108 |
| *Uperoleia marmorata* | 1.05 | 0.0116 |
| *Leptodactylus occelatus* | 20.61 | 0.0808 |
| *Leptodactylus pentadactylus* | 195 | 0.2667 |
| *Leptodactylus wagneri* | 5.25 | 0.0382 |
| *Dendrobates histrionicus* | 2.22 | 0.0277 |
| *Dendrobates pumilio* | 0.52 | 0.0115 |
| *Dendrobates speciosus* | 0.94 | 0.0152 |
| *Phyllobates lugubris* | 1.29 | 0.0209 |
| *Atelopus chiriquiensis* | 2.01 | 0.0208 |
| *Atelopus flavescens* | 2 | 0.0178 |
| *Atelopus varius* | 3.29 | 0.0199 |
| *Bufo bufo* | 128.2 | 0.1546 |
| *Bufo calamita* | 28.59 | 0.0743 |
| *Bufo marinus* | 355 | 0.2433 |
| *Bufo regularis Afrique* | 100 | 0.1495 |
| *Bufo regularis Reunion* | 27.45 | 0.0853 |
| *Bufo spinulosus* | 223 | 0.132 |
| *Bufo typhonius* | 11.22 | 0.0363 |
| *Bufo viridis arabicus* | 48.75 | 0.1401 |
| *Bufo woodhousei* | 28.1 | 0.0742 |
| *Nectophrynoides occidentalis* | 0.75 | 0.0112 |
| *Pseudis paradoxus* | 24.1 | 0.0738 |
| *Centrolenella fleischmanni* | 0.78 | 0.0213 |
| *Litoria bicolor* | 0.97 | 0.0224 |
| *Litoria caerulea* | 112.9 | 0.1535 |
| *Litoria lesuerii* | 1.78 | 0.0274 |
| *Litoria peronii* | 9.63 | 0.0727 |
| *Pseudohemisus pustulosus* | 20.52 | 0.0533 |
| *Breviceps gibbosus* | 6.01 | 0.0427 |
| *Phrynomerus bifasciatus* | 4.57 | 0.029 |
| *Arthroleptis nimbaensis* | 1.5 | 0.0173 |
| *Afrixalus dorsalis* | 1.13 | 0.0149 |
| *Afrixalus fulvovittatus* | 1.06 | 0.0166 |
| *Hyperolius concolor* | 1.17 | 0.0149 |
| *Kassina senegalensis* | 3.95 | 0.028 |
| *Kassina wealii* | 1.01 | 0.0168 |

**References**

1 Jerison, H. J. *Evolution of the brain and intelligence*. (Academic Press, 1973).

2 Weisbecker, V. & Goswami, A. Brain size, life history, and metabolism at the marsupial/placental dichotomy. *Proceedings of the National Academy of Sciences*, doi:10.1073/pnas.0906486107 (2010).

3 Striedter, G. F. *Principles of brain evolution*. (Sinauer Associates, 2005).

4 Boddy, A. M. *et al.* Comparative analysis of encephalization in mammals reveals relaxed constraints on anthropoid primate and cetacean brain scaling. *Journal of evolutionary biology* **25**, 981-994, doi:10.1111/j.1420-9101.2012.02491.x (2012).

5 Sol, D. *et al.* Evolutionary Divergence in Brain Size between Migratory and Resident Birds. *PloS one* **5**, e9617, doi:10.1371/journal.pone.0009617 (2010).

6 Froese, R. a. D. P. E. FishBase, World Wide Web electronic publication. www.fishbase.org, version. (2012).

7 Amiel, J. J., Tingley, R. & Shine, R. Smart moves: effects of relative brain size on establishment success of invasive amphibians and reptiles. *PloS one* **6**, e18277, doi:10.1371/journal.pone.0018277 (2011).
