# Supplementary material for: Effect of temperature and glia in brain size enlargement and origin of allometric body-brain size scaling in vertebrates
Source: BMC Evol Biol. 2014 Oct 3;14:178. doi: 10.1186/s12862-014-0178-z (PMC4193995; doi:10.1186/s12862-014-0178-z)
Supplement: Additional file 2: — Animal datasets for the allometric scaling relation of fish Brain-to-Body mass in different water conditions. Database www.fishbase.org was searched for fish brain-body mass data, of which 35 species living in polar ocean (0–5°C), 70 species living in temperate ocean (10 ~ 20°C), 88 species living in tropical water condition (20 ~ 30°C), and 17 shark species in sub-tropical water condition (15 ~ 25°C) are randomly choosed. These data are listed in Table S2a, S2b, S2c, and S2d. [file 12862_2014_178_MOESM2_ESM.docx]

**Supplement Table S2 Dataset:**

**Allometric scaling relation of fish Brain-to-Body mass in different water conditions**

Database [www.fishbase.org](http://www.fishbase.org) was searched for fish brain-body mass data, of which 35 species living in polar ocean (0-5 ^o^C), 70 species living in temperate ocean (10 ~20 ^o^C), 88 species living in tropical water condition (20~30 ^o^C), and 17 shark species in sub-tropical water condition (15~25 ^o^C) are randomly choosed. These data are listed in **Table S2a, S2b, S2c, and S2d** in the following.

**Table S2a. Fish living in cold water (0-5 ^o^C): 35 species**

| **Species** | **Body weight (gram)** | **Brain weight(gram)** |
| --- | --- | --- |
| *Idiacanthus fasciola* | 6.3 | 0.0058 |
| *Latimeria chalumnae* | 5850 | 0.2 |
| *Latimeria chalumnae* | 30000 | 1.3 |
| *Lampetra fluviatilis* | 174 | 0.015 |
| *Lophius piscatorius* | 14400 | 0.621 |
| *Lophius piscatorius* | 140 | 0.111 |
| *Lophius budegassa* | 500 | 0.183 |
| *Myoxocephalus scorpius* | 80 | 0.12 |
| *Myoxocephalus scorpius* | 550 | 0.286 |
| *Myoxocephalus scorpius* | 1170 | 0.345 |
| *Notacanthus chemnitzii* | 4500 | 0.812 |
| *Petromyzon marinus* | 282 | 0.0175 |
| *Anarhichas denticulatus* | 2900 | 0.272 |
| *Anarhichas denticulatus* | 6000 | 0.346 |
| *Anarhichas denticulatus* | 10500 | 0.637 |
| *Cyclopterus lumpus* | 925 | 0.192 |
| *Cyclopterus lumpus* | 3040 | 0.229 |
| *Leptagonus decagonus* | 20 | 0.035 |
| *Myoxocephalus scorpius* | 80 | 0.121 |
| *Myoxocephalus scorpius* | 550 | 0.286 |
| *Myoxocephalus scorpius* | 1170 | 0.345 |
| *Notothenia rossii* | 1450 | 0.741 |
| *Notothenia rossii* | 2800 | 1.2 |
| *Notothenia rossii* | 450 | 0.52 |
| *Anarhichas minor* | 2250 | 0.458 |
| *Anarhichas minor* | 6400 | 0.754 |
| *Anarhichas minor* | 21000 | 0.967 |
| *Malacosteus niger* | 24 | 0.016 |
| *Stomias boa ferox* | 11 | 0.007 |
| *Stomias boa ferox* | 60 | 0.0166 |
| *Stomias boa ferox* | 27 | 0.02 |
| *Chauliodus sloani* | 32 | 0.03 |
| *Chauliodus sloani* | 66 | 0.0372 |
| *Gonostoma elongatum* | 60 | 0.051 |

**Table S2b. Fish living in temperate water (10-20 ^o^C): 70 species**

| **Species** | **Body weight (gram)** | **Brain weight(gram)** |
| --- | --- | --- |
| *Alosa immaculata* | 0.438 | 350 |
| *Alosa caspia* | 0.234 | 84 |
| *Ammodytes marinus* | 0.015 | 4.3 |
| *Amia calva* | 0.403 | 727 |
| *Belone belone* | 0.421 | 282 |
| *Brosme brosme* | 0.622 | 600 |
| *Callionymus maculatus* | 0.0453 | 8 |
| *Callionymus maculatus* | 0.049 | 20 |
| *Ciliata septentrionalis* | 0.0469 | 10 |
| *Cottunculus microps* | 0.092 | 120 |
| *Gadus morhua* | 0.759 | 610 |
| *Gadus morhua* | 2.223 | 6998 |
| *Gymnogobius castaneus* | 0.01 | 1.5 |
| *Hyperlophus translucidus* | 0.016 | 1.7 |
| *Hypseleotris galii* | 0.006 | 0.8 |
| *Knipowitschia caucasica* | 0.0048 | 0.5 |
| *Lepidonotothen squamifrons* | 0.338 | 300 |
| *Lepidotrigla papilio* | 0.142 | 72 |
| *Lipophrys pholis* | 0.044 | 5 |
| *Liza aurata* | 0.149 | 51 |
| *Macrourus berglax* | 0.405 | 115 |
| *Macrourus berglax* | 1.432 | 1500 |
| *Micromesistius poutassou* | 0.247 | 50 |
| *Micromesistius poutassou* | 0.55 | 250 |
| *Molva molva* | 0.88 | 1500 |
| *Molva molva* | 2.09 | 5998 |
| *Molva molva* | 2.53 | 13000 |
| *Odontobutis obscura* | 0.03 | 20 |
| *Osmerus mordax* | 0.116 | 74 |
| *Pollachius virens* | 2.449 | 9397 |
| *Pollachius virens* | 1.129 | 780 |
| *Pollachius pollachius* | 2.218 | 5998 |
| *Pollachius pollachius* | 0.422 | 122 |
| *Pollachius pollachius* | 0.711 | 214 |
| *Pollachius pollachius* | 0.164 | 20 |
| *Pomatoschistus minutus* | 0.0087 | 1.7 |
| *Pomatoschistus minutus* | 0.032 | 8.5 |
| *Scaphirhynchus platorynchus* | 0.24 | 510 |
| *Scorpaena porcus* | 0.07 | 35 |
| *Scorpaena porcus* | 0.13 | 180 |
| *Sprattus sprattus* | 0.13 | 20 |
| *Squalus acanthias* | 3.87 | 4200 |
| *Squatina squatina* | 2.06 | 6000 |
| *Symphodus melops* | 0.082 | 10.5 |
| *Symphodus melops* | 0.335 | 127 |
| *Tridentiger trigonocephalus* | 0.009 | 1.4 |
| *Conger conger* | 0.405 | 950 |
| *hyperoplus immaculatus* | 0.155 | 110 |
| *Hyperoplus lanceolatus* | 0.05 | 19 |
| *lepomis gibbosus* | 0.14 | 29 |
| *lepidion eques* | 0.3 | 80 |
| *lepidion eques* | 0.678 | 400 |
| *lepidion eques* | 0.429 | 196 |
| *Lutjanus argentimaculatus* | 2.992 | 7400 |
| *Lumpenus lampretaeformis* | 0.08 | 30 |
| *Lipophrys pholis* | 0.037 | 4.2 |
| *Lipophrys pholis* | 0.053 | 10.6 |
| *Merluccius merluccius* | 0.993 | 2999 |
| *Merluccius merluccius* | 1.409 | 10000 |
| *merlangius merlangus* | 0.77 | 800 |
| *Mugil cephalus* | 0.506 | 750 |
| *Mugil cephalus* | 0.3 | 420 |
| *Molva dypterygia* | 2.3 | 8995 |
| *Molva dypterygia* | 1.1 | 1500 |
| *Molva dypterygia* | 0.18 | 55 |
| *Mola mola* | 0.922 | 4600 |
| *Nerophis lumbriciformis* | 0.0034 | 1.3 |
| *Phycis blennoides* | 1.513 | 5998 |
| *Phycis blennoides* | 0.824 | 1250 |
| *Phycis blennoides* | 0.354 | 110 |

**Table S2c.Fish living in Tropical water (20-30 ^o^C): 88 species**

| **Species** | **Body weight (gram)** | **Brain weight(gram)** |
| --- | --- | --- |
| *Abudefduf abdominalis* | 60 | 0.209 |
| *Abudefduf septemfasciatus* | 200 | 0.304 |
| *Acanthurus achilles* | 140 | 0.489 |
| *Adontosternarchus balaenops* | 7 | 0.117 |
| *Acentrogobius viridipunctatus* | 3.6 | 0.02 |
| *Amblycirrhitus bimacula* | 3.3 | 0.033 |
| *Anampses melanurus* | 2.5 | 0.0267 |
| *Amphiprion allardi* | 32 | 0.102 |
| *Amoya gracilis* | 0.7 | 0.01 |
| *Aphareus rutilans* | 5200 | 2.875 |
| *Anyperodon leucogrammicus* | 60 | 0.24 |
| *Anyperodon leucogrammicus* | 690 | 0.711 |
| *Antennablennius australis* | 1.3 | 0.0138 |
| *Apogon cookii* | 2.5 | 0.027 |
| *Apogon ellioti* | 17.2 | 0.075 |
| *Aprion virescens* | 1630 | 1.972 |
| *Atule mate* | 250 | 0.661 |
| *Awaous ocellaris* | 3.6 | 0.0145 |
| *Balistapus undulatus* | 470 | 0.527 |
| *Bathygobius soporator* | 10.8 | 0.0283 |
| *Calotomus spinidens* | 157 | 0.307 |
| *Carangoides fulvoguttatus* | 6200 | 2.934 |
| *Ctenochaetus binotatus* | 40 | 0.247 |
| *Gerres oyena* | 29 | 0.158 |
| *Gobiopsis macrostoma* | 0.2 | 0.0012 |
| *Halichoeres biocellatus* | 15.7 | 0.0926 |
| *Katsuwonus pelamis* | 6500 | 4.659 |
| *Labroides dimidiatus* | 5.7 | 0.0642 |
| *Kyphosus bigibbus* | 105 | 0.274 |
| *Kyphosus bigibbus* | 1510 | 1.299 |
| *Lethrinus lentjan* | 500 | 1.663 |
| *Lethrinus variegatus* | 350 | 1.007 |
| *Lethrinus xanthochilus* | 1680 | 2.369 |
| *Lophogobius cyprinoides* | 8.9 | 0.0167 |
| *Lutjanus gibbus* | 970 | 1.75 |
| *Lutjanus bohar* | 155 | 0.832 |
| *Lutjanus bohar* | 3300 | 2.62 |
| *Macolor niger* | 2620 | 2.724 |
| *Malacanthus brevirostris* | 6.4 | 0.0573 |
| *Macrodontogobius wilburi* | 0.2 | 0.0045 |
| *Meiacanthus anema* | 0.4 | 0.0076 |
| *Megalaspis cordyla* | 140 | 0.627 |
| *Naso brevirostris* | 610 | 1.122 |
| *Naso lituratus* | 280 | 0.82 |
| *Nematogobius maindroni* | 0.6 | 0.0071 |
| *Neoniphon opercularis* | 169 | 0.612 |
| *Neoniphon argenteus* | 37 | 0.26 |
| *Oplopomus oplopomus* | 0.3 | 0.0067 |
| *Oplopomus oplopomus* | 2.9 | 0.015 |
| *Parupeneus barberinus* | 578 | 0.72 |
| *Parupeneus barberinus* | 6.2 | 0.09 |
| *Plagiotremus ewaensis* | 1.8 | 0.018 |
| *Pomatoschistus marmoratus* | 0.4 | 0.0055 |
| *Pomacentrus pavo* | 5.5 | 0.058 |
| *Pseudochromis olivaceus* | 1.4 | 0.021 |
| *Selaroides leptolepis* | 450 | 1.289 |
| *Selaroides leptolepis* | 13 | 0.133 |
| *Sphyraena forsteri* | 300 | 0.737 |
| *Sphyraena forsteri* | 1170 | 1.158 |
| *hippocamp histrix* | 6 | 0.012 |
| *hime japonica* | 72 | 0.16 |
| *Heterotis niloticus* | 8.1 | 0.12 |
| *Heterotis niloticus* | 343 | 1.625 |
| *Hologymnosus annulatus* | 350 | 0.671 |
| *hipposcarus harid --* | 1720 | 1.062 |
| *Hyporthodus septemfasciatus* | 8 | 0.051 |
| *Istigobius ornatus* | 1.2 | 0.0043 |
| *Istigobius decoratus* | 0.6 | 0.0045 |
| *Istigobius decoratus* | 2.7 | 0.0098 |
| *Iracundus signifer* | 16.2 | 0.075 |
| *Kraemeria samoensis* | 0.1 | 0.0016 |
| *Katsuwonus pelamis* | 6500 | 4.66 |
| *Labrisomus bucciferus* | 1.5 | 0.025 |
| *Labrisomus bucciferus* | 4.2 | 0.032 |
| *Labrichthys unilineatus* | 20 | 0.1 |
| *Lethrinus microdon* | 2250 | 1.7 |
| *lutjanus fulvus* | 380 | 0.957 |
| *Melichthys vidua* | 81.5 | 0.341 |
| *Megalaspis cordyla* | 140 | 0.627 |
| *Malacanthus brevirostris* | 6.4 | 0.0573 |
| *Lutjanus lemniscatus* | 212 | 0.6 |
| *Myripristis violacea* | 190 | 0.426 |
| *Myripristis violacea* | 48 | 0.27 |
| *Monotaxis grandoculis* | 371 | 0.888 |
| *Naso* | 610 | 1.122 |
| *Osteoglossum bicirrhosum* | 2.9 | 0.1 |
| *Osteoglossum bicirrhosum* | 275 | 1.7 |
| *Pimelodus pictus* | 2.35 | 0.047 |

**Table S2d. Sharks living in sub-Tropical water (15-25 ^o^C): 17species**

| **Species** | **Body weight (gram)** | **Brain weight(gram)** |
| --- | --- | --- |
| *Carcharhinus longimanus* | 40000 | 35.4 |
| *Carcharhinus leucas* | 83800 | 54.36 |
| *Carcharhinus isodon* | 10870 | 18.75 |
| *Carcharias taurus* | 123000 | 82.55 |
| *Centrophorus granulosus* | 1360 | 3.16 |
| *Centrophorus granulosus* | 40000 | 35.4 |
| *Centrophorus granulosus* | 36240 | 43.32 |
| *Carcharhinus falciformis* | 36240 | 43.32 |
| *Carcharhinus obscurus* | 12000 | 20.76 |
| *Centrophorus harrissoni* | 2820 | 4.31 |
| *Blackbelly lanternshark* | 60 | 0.48 |
| *Ginglymostoma cirratum* | 45300 | 31.65 |
| *Galeocerdo cuvier* | 200000 | 107.5 |
| *Heterodontus francisci* | 2930 | 4.3 |
| *Prionace glauca* | 36100 | 21.21 |
| *Squaliolus laticaudus* | 60 | 0.44 |
| *Rhizoprionodon porosus* | 3750 | 7.18 |
