# Supplementary material for: Effect of temperature and glia in brain size enlargement and origin of allometric body-brain size scaling in vertebrates
Source: BMC Evol Biol. 2014 Oct 3;14:178. doi: 10.1186/s12862-014-0178-z (PMC4193995; doi:10.1186/s12862-014-0178-z)
Supplement: Additional file 3: — Animal datasets for the allometric scaling relation of metabolism-to-body for endothermic and ectothermic animals. Basal metabolic rates for amphibians (Table S3a) and reptiles (Table S3b) were modified from references [37,42]; for fishes (Table S3c) from references [37,42,44]; for birds (Table S3d) [37,42]; and for mamals [43] (which is Additional file 1: Table S1 in this reference, and data is not listed here). [file 12862_2014_178_MOESM3_ESM.docx]

**Supplement Table S3 Dataset:**

**Allometric scaling relation of metabolism-to-body for endothermic and ectothermic animals.** Basal metabolic rates for amphibians (Table S3a) and reptiles (Table S3b) were modified from references[^1^](#_ENREF_1)^,^[^2^](#_ENREF_2); for fishes (Table S3c) from references[^1-3^](#_ENREF_1); for birds(Table S3d) [^1^](#_ENREF_1)^,^[^2^](#_ENREF_2); and for mamals[^4^](#_ENREF_4) (which is supplemental table S1 in this reference, and data is not listed here).

**Table S3a: Standard metabolic rates in amphibians**[**^1^**](#_ENREF_1)**^,^**[**^2^**](#_ENREF_2)

| Number | Species | Body mass (gram) | T (^o^C) | Basal metabolic rate (watts) |
| --- | --- | --- | --- | --- |
| *1* | *Acris crepitans* | 1.59 | 15 | 2.66E-04 |
| *2* | *Agalychnis callidryas* | 5.65 | 20 | 0.00187 |
| *3* | *Ambystoma gracile* | 30.39 | 15 | 0.00353 |
| *4* | *Ambystoma jeffersonianum* | 8.92 | 15 | 1.76E-03 |
| *5* | *Ambystoma macrodactylum* | 3.414 | 15 | 6.11E-04 |
| *6* | *Ambystoma maculatum* | 15.83 | 15 | 0.00201 |
| *7* | *Ambystoma mexicanum* | 21.3 | 22 | 1.77E-03 |
| *8* | *Ambystoma opacum* | 4.65 | 14 | 4.79E-04 |
| *9* | *Ambystoma talpoideum* | 7 | 15 | 3.11E-03 |
| *10* | *Ambystoma tigrinum* | 10.78 | 15 | 7.65E-04 |
| *11* | *Amphiuma means* | 352 | 5 | 0.00493 |
| *12* | *Amphiuma tridactylum* | 652 | 15 | 1.37E-02 |
| *13* | *Aneides ferreus* | 2.73 | 15 | 4.12E-04 |
| *14* | *Aneides flavipunctatus* | 4.57 | 15 | 6.67E-04 |
| *15* | *Aneides hardii* | 0.97 | 20 | 2.85E-04 |
| *16* | *Aneides lugubris* | 5.58 | 15 | 8.31E-04 |
| *17* | *Batrachoseps attenuatus* | 0.93 | 25 | 4.45E-04 |
| *18* | *Bolitoglossa franklini* | 3.18 | 15 | 3.05E-04 |
| *19* | *Bolitoglossa morio* | 2.09 | 15 | 2.47E-04 |
| *20* | *Bolitoglossa occidentalis* | 0.97 | 15 | 1.13E-04 |
| *21* | *Bolitoglossa subpalmata* | 1.63 | 5 | 8.80E-05 |
| *22* | *Bombina orientalis* | 2.6 | 20 | 8.09E-04 |
| *23* | *Boulengerula taitanus* | 5 | 35 | 0.00478 |
| *24* | *Bufo alvaris* | 150.8 | 15 | 0.0659 |
| *25* | *Bufo americanus* | 50 | 20 | 0.0077 |
| *26* | *Bufo boreas* | 57.8 | 20 | 0.00584 |
| *27* | *Bufo bufo* | 76.13 | 15 | 0.01584 |
| *28* | *Bufo calamita* | 8.7 | 20 | 0.0028 |
| *29* | *Bufo cognatus* | 40.2 | 25 | 1.64E-02 |
| *30* | *Bufo debilus* | 0.66 | 15 | 4.69E-04 |
| *31* | *Bufo marinus* | 145 | 15 | 0.00855 |
| *32* | *Bufo terrestris* | 19.8 | 25 | 0.01245 |
| *33* | *Bufo viridis* | 35 | 20 | 4.87E-03 |
| *34* | *Bufo woodhousii* | 56.3 | 25 | 1.24E-02 |
| *35* | *Ceratophrys calcarata* | 55.5 | 25 | 1.65E-02 |
| *36* | *Chiromantis petersi* | 11.2 | 25 | 4.41E-03 |
| *37* | *Chiropterotriton bromeliacia* | 0.59 | 15 | 8.02E-05 |
| *38* | *Colostethus inguinalis* | 1.57 | 20 | 7.58E-04 |
| *39* | *Colostethus nubicola* | 0.27 | 25 | 2.42E-04 |
| *40* | *Colostethus trinitatus* | 1 | 25 | 0.00112 |
| *41* | *Conraua goliath* | 251 | 25 | 1.05E-01 |
| *42* | *Crinia parinsignifera* | 0.452 | 30 | 9.50E-04 |
| *43* | *Crinia signifera* | 0.621 | 25 | 8.28E-04 |
| *44* | *Cryptobranchus alleganiensis* | 423 | 25 | 7.28E-02 |
| *45* | *Cyclorana platycephala* | 11 | 15 | 3.28E-03 |
| *46* | *Dendrobates auratus* | 1.77 | 25 | 8.85E-04 |
| *47* | *Desmognathus fuscus* | 2.09 | 20 | 4.97E-04 |
| *48* | *Desmognathus monticola* | 3.43 | 20 | 8.09E-04 |
| *49* | *Desmognathus ochrophaes* | 1.6 | 17.5 | 2.37E-04 |
| *50* | *Desmognathus quadramaculatus* | 23.41 | 5 | 9.13E-04 |
| *51* | *Dicamptodon ensatus* | 101.25 | 15 | 0.00729 |
| *52* | *Discoglossus pictus* | 30.7 | 20 | 6.32E-03 |
| *53* | *Eleutherodactylus coqui* | 4.06 | 20 | 9.30E-04 |
| *54* | *Eleutherodactylus portoricensis* | 3.7 | 15 | 1.42E-03 |
| *55* | *Ensatina eschscholtzi* | 5.3 | 25 | 1.98E-03 |
| *56* | *Eurycea bislineata* | 1.14 | 5 | 8.78E-05 |
| *57* | *Eurycea longicauda* | 1.57 | 15 | 2.79E-04 |
| *58* | *Eurycea multiplicata* | 0.71 | 15 | 8.59E-05 |
| *59* | *Eurycea nana* | 0.154 | 25 | 7.33E-05 |
| *60* | *Eurycea neotenes* | 0.243 | 25 | 1.43E-04 |
| *61* | *Eurycea pterophila?* | 0.196 | 25 | 9.78E-05 |
| *62* | *Gastrophryne carolinesis* | 1.9 | 20 | 5.49E-04 |
| *63* | *Geotrypetes seraphini* | 1.93 | 20 | 3.98E-04 |
| *64* | *Gyrinophilus danielsi* | 14.44 | 15 | 0.00179 |
| *65* | *Gyrinophilus porphyrictus* | 12.4 | 15.5 | 1.04E-03 |
| *66* | *Hydromantes sp.* | 3.23 | 15 | 6.88E-04 |
| *67* | *Hyla arborea* | 7.78 | 18.5 | 0.01358 |
| *68* | *Hyla arenicolor* | 3.37 | 20 | 1.67E-03 |
| *69* | *Hyla chrysoscelis* | 3.9 | 10 | 8.23E-04 |
| *70* | *Hyla cinerea* | 4.5 | 25 | 0.00255 |
| *71* | *Hyla crepitans* | 9.9 | 25 | 6.38E-03 |
| *72* | *Hyla crucifer* | 1.15 | 20 | 7.03E-04 |
| *73* | *Hyla gratiosa* | 13.9 | 29 | 0.00734 |
| *74* | *Hyla maxima* | 41.4 | 25 | 2.19E-02 |
| *75* | *Hyla regilla* | 2.27 | 21 | 4.95E-04 |
| *76* | *Hyla squirella* | 2.2 | 27 | 2.02E-03 |
| *77* | *Hyla versicolor* | 8.62 | 19 | 3.73E-03 |
| *78* | *Hyperolius marmoratus* | 1 | 20 | 3.73E-04 |
| *79* | *Hyperolius parallelus* | 1 | 20 | 4.25E-04 |
| *80* | *Hyperolius tuberilinguis* | 1 | 20 | 3.77E-04 |
| *81* | *Hyperolius viridiflavus* | 0.9 | 20 | 4.70E-04 |
| *82* | *Kaloula pulchra* | 30.7 | 20 | 0.00442 |
| *83* | *Kassina senegalensis* | 3.02 | 20 | 0.00127 |
| *84* | *Kassina weali* | 6.25 | 20 | 0.00177 |
| *85* | *Lepidobatrachus llanensis* | 88.5 | 25 | 0.0477 |
| *86* | *Leptodactylus typhonius* | 5.1 | 25 | 3.26E-03 |
| *87* | *Microhyla carolinensis* | 3.5 | 15 | 0.00128 |
| *88* | *Molga torosa?* | 17.5 | 18.5 | 0.01517 |
| *89* | *Necturus maculosus* | 125 | 25 | 1.38E-02 |
| *90* | *Notophthalmus viridescens* | 3 | 20 | 7.50E-04 |
| *91* | *Occidozyga martensii* | 9.3 | 35 | 0.00599 |
| *92* | *Odontophrynus americanus* | 15.24 | 20 | 3.05E-03 |
| *93* | *Osteopilus septentrionalis* | 5 | 20 | 1.84E-03 |
| *94* | *Phyllomedusa sauvagei* | 17.5 | 10 | 2.82E-03 |
| *95* | *Physalaemus pustulosus* | 2.9 | 25 | 1.89E-03 |
| *96* | *Plethodon cinereus* | 0.6 | 17.5 | 9.36E-05 |
| *97* | *Plethodon dorsalis* | 0.69 | 25 | 1.32E-04 |
| *98* | *Plethodon glutinosus* | 5.01 | 20 | 9.27E-04 |
| *99* | *Plethodon jordani* | 3.1 | 5 | 1.49E-04 |
| *100* | *Plethodon neomexicanus* | 2.47 | 25 | 8.37E-04 |
| *101* | *Plethodon spp.* | 3.25 | 22 | 1.03E-03 |
| *102* | *Pseudacris nigrita* | 1 | 25 | 9.00E-04 |
| *103* | *Pseudacris triseriata* | 0.94 | 5 | 7.80E-05 |
| *104* | *Pseudobranchus striatus* | 2.19 | 25 | 6.20E-04 |
| *105* | *Pseudoeurycea belli* | 24.45 | 25 | 3.99E-03 |
| *106* | *Pseudoeurycea brunnata* | 3.33 | 15 | 3.65E-03 |
| *107* | *Pseudoeurycea cephalica* | 1.45 | 15 | 1.97E-04 |
| *108* | *Pseudoeurycea cochranae* | 2.23 | 15 | 2.54E-04 |
| *109* | *Pseudoeurycea gadovii* | 2.88 | 15 | 4.72E-04 |
| *110* | *Pseudoeurycea goebeli* | 3.78 | 5 | 2.00E-04 |
| *111* | *Pseudoeurycea leprosa* | 2.46 | 15 | 3.10E-04 |
| *112* | *Pseudoeurycea rex* | 1.86 | 15 | 2.18E-04 |
| *113* | *Pseudoeurycea smithii* | 6.66 | 15 | 6.59E-04 |
| *114* | *Pseudotriton ruber* | 10.81 | 5 | 3.78E-04 |
| *115* | *Pternohyla fodiens* | 15.1 | 20 | 0.00269 |
| *116* | *Pyxicephalus adspersus* | 562.3 | 20 | 0.07928 |
| *117* | *Rana arvalis* | 17 | 5 | 0.00235 |
| *118* | *Rana aspersa* | 563 | 18.5 | 1.76E-01 |
| *119* | *Rana berlandieri* | 70 | 29 | 0.03906 |
| *120* | *Rana blythi* | 88.7 | 25 | 0.0243 |
| *121* | *Rana cancrivora* | 20.45 | 30 | 1.26E-02 |
| *122* | *Rana catesbeiana* | 262 | 5 | 7.34E-03 |
| *123* | *Rana chalconota* | 4.1 | 25 | 0.00217 |
| *124* | *Rana clamitans* | 32.5 | 25 | 0.01502 |
| *125* | *Rana cyanophlyctis* | 0.294 | 29 | 1.20E-03 |
| *126* | *Rana erythraea* | 19 | 15 | 9.88E-04 |
| *127* | *Rana esculenta* | 15.2 | 23 | 0.0014 |
| *128* | *Rana hexadactyla* | 51.9 | 29 | 0.06659 |
| *129* | *Rana magna* | 34.2 | 30 | 1.58E-02 |
| *130* | *Rana muscosa* | 13.5 | 4 | 5.54E-04 |
| *131* | *Rana nicobariensis* | 2.6 | 25 | 0.00142 |
| *132* | *Rana palustris* | 36 | 21.5 | 2.04E-02 |
| *133* | *Rana pipiens* | 34.8 | 10 | 0.00271 |
| *134* | *Rana ridibunda* | 35 | 20 | 0.00623 |
| *135* | *Rana sylvatica* | 6 | 25 | 0.0036 |
| *136* | *Rana temporaria* | 39 | 15 | 1.56E-03 |
| *137* | *Rana virgatipes* | 7 | 5 | 7.77E-04 |
| *138* | *Rhyacotriton olympicus* | 2.6 | 15 | 0.00139 |
| *139* | *Salamandra maculosa* | 28.32 | 18.5 | 0.02025 |
| *140* | *Salamandra salamandra* | 75 | 16 | 6.30E-03 |
| *141* | *Scaphiopus bombifrons* | 13.3 | 15 | 0.01028 |
| *142* | *Scaphiopus couchii* | 24.6 | 21 | 1.01E-02 |
| *143* | *Scaphiopus hammondii* | 12.74 | 21 | 6.37E-03 |
| *144* | *Scaphiopus holbrooki* | 14 | 15 | 0.00651 |
| *145* | *Siren intermedia* | 13.7 | 25 | 2.51E-03 |
| *146* | *Siren lacertina* | 269 | 25 | 0.01695 |
| *147* | *Smilisca baudinii* | 23.7 | 15 | 8.79E-03 |
| *148* | *Taricha granulosa* | 6.56 | 15 | 8.92E-04 |
| *149* | *Taricha rivularis* | 10.83 | 15 | 1.67E-03 |
| *150* | *Taricha torosa* | 9.87 | 10 | 4.94E-04 |
| *151* | *Telmatobius culeus* | 122.3 | 10 | 0.00954 |
| *152* | *Telmatobius marmoratus* | 18.4 | 10 | 5.12E-03 |
| *153* | *Thorius sp.* | 0.31 | 15 | 4.43E-05 |
| *154* | *Triturus cristatus* | 7 | 10 | 0.00214 |
| *155* | *Triturus vulgaris* | 8.75 | 25 | 0.00904 |
| *156* | *Typhlonectes compressicauda* | 30.62 | 25 | 0.00802 |
| *157* | *Xenopus laevis* | 139 | 20 | 0.02127 |
| *158* | *Xenopus mulleri* | 24 | 17 | 0.01394 |
|  |  |  |  |  |

**Table S3b: Standard metabolic rates in reptiles**[**^1^**](#_ENREF_1)**^,^**[**^2^**](#_ENREF_2)

| Number | Species | Body mass (gram) | T (^o^C) | Basal metabolic rate (watts) |
| --- | --- | --- | --- | --- |
| *1* | *Acabthodactylus boskianus* | 7.8 | 40 | 0.013 |
| *2* | *Acanthodactylus erythrurus* | 9 | 35 | 0.01751 |
| *3* | *Acanthodactylus opheodurus* | 3.8 | 30 | 0.00439 |
| *4* | *Acanthodactylus pardalis* | 9.7 | 40 | 0.03772 |
| *5* | *Acanthodactylus schmidti* | 14.5 | 35 | 0.01788 |
| *6* | *Acanthodactylus schreiberi* | 10.9 | 40 | 0.0169 |
| *7* | *Acanthodactylus scutellatus* | 6.6 | 40 | 0.01687 |
| *8* | *Acanthophis praelongus* | 105.5 | 30 | 0.02754 |
| *9* | *Acontias meleagris* | 7.3 | 33 | 0.00377 |
| *10* | *Acrantophis dumerili* | 2548.5 | 20 | 0.10704 |
| *11* | *Acrochordus aradurae* | 1047.7 | 30 | 0.13725 |
| *12* | *Aligator mississippiensis* | 1287 | 10 | 0.0399 |
| *13* | *Amblyrhynchus cristatus* | 747.5 | 25 | 0.15174 |
| *14* | *Amphibolurus barbatus* | 373 | 37 | 0.29019 |
| *15* | *Amphibolurus nuchalis* | 24.65 | 40 | 0.03301 |
| *16* | *Anarbylus switaki* | 9.48 | 25 | 0.00389 |
| *17* | *Anguis fragilis* | 12.067 | 30 | 0.00395 |
| *18* | *Anniella pulchra* | 4.8466 | 25 | 0.00176 |
| *19* | *Anolis acutus* | 4.3 | 30 | 0.00693 |
| *20* | *Anolis bonariensis* | 12 | 27 | 0.00433 |
| *21* | *Anolis carolinensis* | 4.5 | 30 | 4.70E-03 |
| *22* | *Anolis limifrons* | 1.5 | 20 | 6.66E-04 |
| *23* | *Antaresia childreni* | 331.7 | 24 | 0.06435 |
| *24* | *Antaresia stimsoni* | 349.9 | 30 | 0.11092 |
| *25* | *Aspidites melanocephalus* | 1027.5 | 24 | 0.20961 |
| *26* | *Blanus cinereus* | 2.4 | 30 | 0.00235 |
| *27* | *Boa constrictor* | 7815.5 | 30 | 0.70339 |
| *28* | *Bunopus tuberculatus* | 2.5 | 35 | 0.01421 |
| *29* | *Candoia carinatus* | 521.9 | 20 | 0.0214 |
| *30* | *Chalcides ocellatus* | 22.06 | 33 | 0.01152 |
| *31* | *Chelydra serpentina* | 3473 | 10 | 0.06599 |
| *32* | *Chironius quadricarinatus* | 61 | 20 | 0.01153 |
| *33* | *Cnemidophorus murinus* | 85 | 40 | 0.08644 |
| *34* | *Cnemidophorus tigris* | 18 | 30 | 0.014 |
| *35* | *Coleonyx variegatus* | 3.43 | 25 | 0.00279 |
| *36* | *Coluber constrictor* | 262 | 35 | 0.09327 |
| *37* | *Corallus caninus* | 556 | 20 | 0.02724 |
| *38* | *Corallus enhydris* | 802 | 30 | 0.10426 |
| *39* | *Cosymbotus platyurus* | 3.5 | 27 | 0.00195 |
| *40* | *Crotalus viridis* | 301 | 35 | 0.10716 |
| *41* | *Crotaphytus collaris* | 30 | 37 | 0.04668 |
| *42* | *Ctenotus labillardieri* | 2.8 | 20 | 0.00111 |
| *43* | *Cyclagras gigas* | 2680 | 20 | 0.47704 |
| *44* | *Diadophis punctatus* | 4.4 | 30 | 0.00323 |
| *45* | *Diplometopon zarudnyi* | 6.34 | 35 | 0.00539 |
| *46* | *Dipsas albifrons* | 22 | 20 | 0.0044 |
| *47* | *Dipsosaurus dorsalis* | 40.36 | 45 | 0.05606 |
| *48* | *Egernia cunninghami* | 261 | 20 | 0.04933 |
| *49* | *Elaphe guttata* | 800 | 25 | 0.5024 |
| *50* | *Epicrates cenchria* | 416 | 30 | 0.06739 |
| *51* | *Eryx colubrinus* | 85.85 | 20 | 0.00704 |
| *52* | *Eumeces fasciatus* | 7 | 30 | 0.00933 |
| *53* | *Eumeces inexpectatus* | 9.6 | 30 | 0.00816 |
| *54* | *Eumeces obsoletus* | 30 | 20 | 0.00834 |
| *55* | *Eunectes murinus* | 1130 | 20 | 0.13221 |
| *56* | *Eunectes notaeus* | 14400 | 20 | 1.20E+00 |
| *57* | *Garthia gaudichaudi* | 0.805 | 25 | 4.27E-04 |
| *58* | *Gekko gecko* | 61.5 | 30 | 0.03296 |
| *59* | *Gerrhonotus multicarinatus* | 29 | 20 | 0.00916 |
| *60* | *Gonotodes antillensis* | 1.8 | 34 | 0.00166 |
| *61* | *Helicops modestus* | 196 | 30 | 0.05998 |
| *62* | *Hemidactylus frenatus* | 2 | 27 | 0.00117 |
| *63* | *Iguana iguana* | 795 | 37 | 0.66224 |
| *64* | *Klauberina riversiana* | 19 | 35 | 0.00211 |
| *65* | *Lacerta agilis* | 8.4 | 35 | 0.02273 |
| *66* | *Lacerta sicula* | 9 | 20 | 0.00272 |
| *67* | *Lacerta trilineata* | 71 | 20 | 0.00902 |
| *68* | *Lacerta viridis* | 31 | 20 | 0.00465 |
| *69* | *Lacerta vivipara* | 3.95 | 30 | 0.0052 |
| *70* | *Lampropeltis getulus* | 1217 | 26 | 0.18985 |
| *71* | *Lampropeltis miliaris* | 401 | 25 | 0.10707 |
| *72* | *Leimadophis poecilogyrus* | 42 | 20 | 9.32E-03 |
| *73* | *Lepidophyma gaigeae* | 5 | 15 | 7.50E-04 |
| *74* | *Lepidophyma smithi* | 25 | 30 | 0.00973 |
| *75* | *Liasis fuscus* | 1306.9 | 30 | 0.15944 |
| *76* | *Liasis olivaceus* | 3000.7 | 24 | 0.24906 |
| *77* | *Lichanura roseofusca* | 314 | 32 | 0.12215 |
| *78* | *Lichanura trivirgata* | 182 | 20 | 0.01329 |
| *79* | *Masticodryas bifossatus* | 735 | 20 | 0.17566 |
| *80* | *Masticophis flagellum* | 262 | 35 | 1.23743 |
| *81* | *Morelia spilota variegata* | 2173.5 | 30 | 3.13E-01 |
| *82* | *Natrix maura* | 22.5 | 5 | 8.10E-04 |
| *83* | *Natrix natrix helretica* | 82.5 | 5 | 0.00182 |
| *84* | *Nerodia rhombifera* | 238 | 30 | 0.12566 |
| *85* | *Ophisaurus ventralis* | 32.185 | 25 | 0.00689 |
| *86* | *Oxyrhopus trigeminus* | 98 | 20 | 0.01901 |
| *87* | *Pelamis platurus* | 116 | 30 | 0.03863 |
| *88* | *Philodryas olfersii* | 176 | 20 | 0.03819 |
| *89* | *Philodryas patagoniensis* | 388 | 20 | 0.1098 |
| *90* | *Philodryas serra* | 135 | 20 | 0.02619 |
| *91* | *Phrynosoma cornutum* | 35 | 35 | 0.03227 |
| *92* | *Phrynosoma douglassi* | 28 | 35 | 0.0266 |
| *93* | *Phrynosoma m'calli* | 16 | 37 | 0.02445 |
| *94* | *Physignathus lesueurii* | 504 | 37 | 0.39211 |
| *95* | *Pituophis melanolecus* | 548 | 20 | 0.05809 |
| *96* | *Pitupophis catenifer affinis* | 548 | 30 | 1.22E-01 |
| *97* | *Podarcis hispanica* | 3.43 | 10 | 3.81E-04 |
| *98* | *Podarcis lilfordi brauni* | 7.4 | 20 | 0.003 |
| *99* | *Podarcis muralis* | 5.5 | 35 | 0.01305 |
| *100* | *Psammodromus algirus* | 5.2 | 20 | 0.00355 |
| *101* | *Pseudemys scripta* | 305 | 10 | 0.00641 |
| *102* | *Pseudonaja nuchalis* | 214.1 | 33 | 0.088 |
| *103* | *Ptyodactylus hasselquistii* | 8.5 | 30 | 0.00689 |
| *104* | *Python curtis* | 2373.5 | 20 | 0.09731 |
| *105* | *Python molurus* | ######### | 20 | 0.83376 |
| *106* | *Python regius* | 787 | 20 | 0.03856 |
| *107* | *Python reticulatus* | 14326 | 20 | 0.7163 |
| *108* | *Python sebae* | 16140 | 20 | 0.62946 |
| *109* | *Salvadora hexalepis* | 65 | 30 | 0.03861 |
| *110* | *Sauromalus hispidus* | 574 | 20 | 0.05683 |
| *111* | *Sauromalus obesus* | 150 | 20 | 0.02415 |
| *112* | *Sceloporus graciosus* | 5 | 25 | 0.00245 |
| *113* | *Sceloporus occidentalis* | 10.105 | 25 | 0.00606 |
| *114* | *Sceloporus olivaceus* | 24 | 20 | 8.78E-03 |
| *115* | *Sceloporus undulatus* | 3.8 | 20 | 8.25E-04 |
| *116* | *Sceloporus variabilis* | 12.215 | 10 | 0.0112 |
| *117* | *Scelotes gronovii* | 1.1 | 33 | 1.39E-03 |
| *118* | *Scincella lateralis* | 1 | 20 | 6.67E-04 |
| *119* | *Scinus mitranus* | 14.6 | 35 | 0.01216 |
| *120* | *Sibynomorphis mikanii* | 11 | 20 | 0.00751 |
| *121* | *Spalerosophis cliffordi* | 300 | 35 | 3.17E-01 |
| *122* | *Sphaerodactylus beattyi* | 0.4 | 30 | 5.00E-04 |
| *123* | *Sphaerodactylus cinereus* | 0.4 | 27 | 2.22E-04 |
| *124* | *Sphaerodactylus macrolepis* | 0.5 | 30 | 7.23E-04 |
| *125* | *Sphenodon punctatum* | 430 | 25 | 0.07396 |
| *126* | *Sphenops sepsoides* | 7.4 | 30 | 0.00325 |
| *127* | *Storeria dekayi* | 7.2 | 30 | 0.00648 |
| *128* | *Tarentola mauritanica* | 6.6 | 35 | 0.00667 |
| *129* | *Terrapene ornata ornata* | 354 | 10 | 0.00142 |
| *130* | *Thamnodyastes strigatus* | 55 | 20 | 0.01254 |
| *131* | *Thamnophis butleri* | 19.02 | 25 | 0.0065 |
| *132* | *Thamnophis proximus* | 31 | 30 | 0.02582 |
| *133* | *Thamnophis sirtalis* | 200 | 29 | 0.0366 |
| *134* | *Tiliqua rugosa* | 508.6 | 35 | 0.40688 |
| *135* | *Tiliqua scincoides* | 493 | 20 | 0.07395 |
| *136* | *Trachydosaurus rugosus* | 461 | 20 | 0.07699 |
| *137* | *Trogonophis weigmanni* | 4.985 | 25 | 0.00107 |
| *138* | *Uromastyx microlepis* | 289.84 | 20 | 0.01942 |
| *139* | *Uta mearnsi* | 14 | 20 | 0.00304 |
| *140* | *Uta stansburiana* | 3.625 | 37 | 0.00604 |
| *141* | *Varanus albigularis* | 963 | 35 | 0.82914 |
| *142* | *Varanus bengalensis* | 3440 | 30 | 0.97352 |
| *143* | *Varanus exanthematicus* | 3836 | 25 | 10.52215 |
| *144* | *Varanus giganteus* | 2496 | 25.9 | 0.59405 |
| *145* | *Varanus gilleni* | 27.5 | 37 | 0.02978 |
| *146* | *Varanus gouldi* | 674 | 20 | 0.08964 |
| *147* | *Varanus mertensi* | 904 | 35 | 0.39143 |
| *148* | *Varanus panoptes* | 2005 | 20.5 | 0.30476 |
| *149* | *Varanus varius* | 4410 | 30 | 1.22598 |
| *150* | *Varnus rosenbergi* | 1269.3 | 35 | 0.9304 |
| *151* | *Vipera berus* | 63 | 25 | 3.15E-02 |
| *152* | *Xantusia henshawi* | 3.5 | 15 | 7.21E-04 |
| *153* | *Xantusia vigilis* | 1.5 | 30 | 0.00144 |
| *154* | *Xenodon guentheri* | 50 | 20 | 0.0097 |
| *155* | *Xenodon merremii* | 502 | 20 | 0.11998 |
|  |  |  |  |  |
|  |  |  |  |  |

**Table S3c: Standard metabolic rates in fishes**[**^2^**](#_ENREF_2)**^,^**[**^3^**](#_ENREF_3)**.**

| Number | Species | Body mass (gram) | T (^o^C) | Basal metabolic rate (watts) |
| --- | --- | --- | --- | --- |
| 1 | Abramis brama | 102.9 | 18 | 0.05404 |
| 2 | Acanthopagrus schlegelii schlegelii | 254 | 25 | 0.02964 |
| 3 | Acipenser gueldenstaedtii | 208 | 18 | 0.05664 |
| 4 | Acipenser nudiventris | 89 | 19 | 0.04708 |
| 5 | Acipenser ruthenus | 258 | 20 | 0.03514 |
| 6 | Acipenser stellatus | 6500 | 20 | 1.7446 |
| 7 | Acipenser transmontanus | 950 | 15 | 0.01112 |
| 8 | Aequidens pulcher | 5 | 25 | 0.00237 |
| 9 | Alburnus alburnus | 9 | 8 | 0.0028 |
| 10 | Ambassis interrupta | 5 | 25 | 0.00158 |
| 11 | Ameiurus melas | 50 | 17 | 0.0142 |
| 12 | Ameiurus natalis | 20 | 15 | 0.00405 |
| 13 | Ameiurus nebulosus | 116 | 10 | 0.00902 |
| 14 | Anabas testudineus | 27.62 | 28 | 5.39E-04 |
| 15 | Anarhichas minor | 27.5 | 1.35 | 0.00385 |
| 16 | Anguilla anguilla | 71.1 | 14 | 0.00525 |
| 17 | Anguilla australis australis | 700 | 20 | 0.05992 |
| 18 | Anguilla japonica | 325 | 14.5 | 0.02528 |
| 19 | Anguilla rostrata | 315 | 15 | 0.02574 |
| 20 | Anoplogaster cornuta | 36.7 | 5 | 4.29E-04 |
| 21 | Aphanius dispar dispar | 0.56 | 17 | 7.28E-04 |
| 22 | Arapaima gigas | 2300 | 28 | 0.22379 |
| 23 | Aristostomias lunifer | 21.1 | 5 | 0.00123 |
| 24 | Bajacalifornia burragei | 24.9 | 5 | 7.74E-04 |
| 25 | Balistes capriscus | 320 | 17.5 | 0.10458 |
| 26 | Bathylagus antarcticus | 27.15 | 0.25 | 0.00274 |
| 27 | Bathylagus stilbius | 8.55 | 5 | 0.00116 |
| 28 | Bathylagus wesethi | 1.5 | 10 | 7.94E-04 |
| 29 | Benthalbella elongata | 35.3 | 0.25 | 0.00728 |
| 30 | Boreogadus saida | 110 | 1 | 0.0077 |
| 31 | Borostomias panamensis | 110.25 | 5 | 0.01073 |
| 32 | Brevoortia tyrannus | 78.4 | 10 | 0.03111 |
| 33 | Callionymus lyra | 105 | 11.5 | 0.00858 |
| 34 | Campostoma anomalum | 19.25 | 17.9 | 0.014 |
| 35 | Caranx hippos | 38.3 | 14.7 | 0.03352 |
| 36 | Carassius auratus auratus | 3.8 | 5 | 1.77E-04 |
| 37 | Carassius carassius | 12.5 | 5 | 4.86E-04 |
| 38 | Catostomus commersonii | 100 | 10 | 0.01362 |
| 39 | Catostomus tahoensis | 43.73 | 8 | 0.01072 |
| 40 | Centropristis striata | 84 | 25 | 0.03268 |
| 41 | Chaenocephalus aceratus | 1130 | 2 | 0.0791 |
| 42 | Channa marulius | 93 | 30 | 0.01447 |
| 43 | Channa orientalis | 30 | 30 | 0.0063 |
| 44 | Channa punctata | 12.5 | 26 | 8.75E-04 |
| 45 | Channa striata | 82 | 30 | 0.01372 |
| 46 | Channichthys rhinoceratus | 200 | 5.5 | 0.05368 |
| 47 | Chanos chanos | 0.7 | 25 | 0.00188 |
| 48 | Chiasmodon niger | 76.6 | 2.5 | 0.01162 |
| 49 | Chiloscyllium plagiosum | 880 | 23 | 0.16086 |
| 50 | Chromis chromis | 10.4 | 16 | 0.00538 |
| 51 | Cichlasoma bimaculatum | 11 | 22 | 0.00252 |
| 52 | Cirrhinus cirrhosus | 1821 | 21.5 | 0.32578 |
| 53 | Citharichthys stigmaeus | 15 | 15 | 0.00467 |
| 54 | Clarias batrachus | 77 | 26 | 0.01648 |
| 55 | Clinocottus analis | 37 | 20 | 0.01756 |
| 56 | Colisa fasciata | 0.92 | 28 | 3.72E-04 |
| 57 | Colossoma macropomum | 1760 | 25 | 0.34232 |
| 58 | Conger conger | 545 | 13 | 0.1823 |
| 59 | Coregonus autumnalis | 234 | 7.2 | 0.12561 |
| 60 | Coregonus fera | 0.11 | 14 | 2.57E-04 |
| 61 | Coregonus sardinella | 365 | 9.2 | 0.15476 |
| 62 | Coryphaena equiselis | 0.03 | 23 | 1.66E-04 |
| 63 | Coryphaena hippurus | 0.9 | 13.5 | 0.00158 |
| 64 | Coryphaenoides armatus | 1200 | 3 | 0.01872 |
| 65 | Cottus gobio | 2.9 | 18 | 0.004 |
| 66 | Ctenopharyngodon idella | 16.05 | 22 | 0.00687 |
| 67 | Cyclothone acclinidens | 0.87 | 3 | 8.13E-05 |
| 68 | Cyclothone microdon | 0.78 | 0.25 | 6.98E-05 |
| 69 | Cyprinodon variegatus variegatus | 2.4 | 25 | 0.00159 |
| 70 | Cyprinus carpio carpio | 174 | 10 | 0.0115 |
| 71 | Dactylopterus volitans | 0.02 | 23 | 2.59E-04 |
| 72 | Dasyatis sabina | 503 | 22.2 | 0.12329 |
| 73 | Diaphus theta | 2.65 | 5 | 0.00158 |
| 74 | Diplodus sargus sargus | 25.75 | 14 | 0.01893 |
| 75 | Dorosoma cepedianum | 87.7 | 13.4 | 0.02047 |
| 76 | Echiichthys vipera | 10.5 | 18.5 | 0.00939 |
| 77 | Electrona antarctica | 7.9 | 0.25 | 0.00184 |
| 78 | Embiotoca lateralis | 599 | 15 | 0.2493 |
| 79 | Encheliophis homei | 7.5 | 30 | 0.00251 |
| 80 | Engraulis japonicus | 628.79 | 16.2 | 0.51121 |
| 81 | Epinephelus akaara | 281 | 25 | 0.0492 |
| 82 | Erimyzon oblongus | 25.8 | 18.5 | 0.01536 |
| 83 | Erpetoichthys calabaricus | 27.4 | 27 | 0.01226 |
| 84 | Esomus danricus | 0.62 | 27.5 | 0.00117 |
| 85 | Esox lucius | 600 | 5 | 0.04668 |
| 86 | Esox masquinongy | 12.2 | 5 | 0.00479 |
| 87 | Etheostoma blennioides | 12.35 | 17.5 | 0.01009 |
| 88 | Euthynnus affinis | 2260 | 24 | 4.42214 |
| 89 | Exodon paradoxus | 3.85 | 20 | 5.69E-04 |
| 90 | Fundulus grandis | 31.5 | 25 | 0.0011 |
| 91 | Fundulus heteroclitus heteroclitus | 9 | 5 | 4.55E-04 |
| 92 | Fundulus parvipinnis | 6 | 20 | 1.40E-04 |
| 93 | Fundulus similis | 21 | 25 | 6.53E-04 |
| 94 | Gadus morhua | 6650 | 3 | 0.72418 |
| 95 | Gadus ogac | 180 | 0 | 0.04482 |
| 96 | Gambusia affinis | 0.24 | 10 | 1.56E-04 |
| 97 | Gambusia holbrooki | 0.3 | 27 | 1.00E-04 |
| 98 | Gasterosteus aculeatus aculeatus | 1.95 | 10 | 8.34E-04 |
| 99 | Genyagnus monopterygius | 164 | 17 | 0.00638 |
| 100 | Gilchristella aestuaria | 1.44 | 15 | 9.13E-04 |
| 101 | Gillichthys mirabilis | 16.7 | 20 | 3.89E-04 |
| 102 | Girella nigricans | 210 | 18 | 0.07025 |
| 103 | Glossogobius giuris | 15 | 26 | 0.00198 |
| 104 | Gnathonemus petersii | 5.85 | 26 | 0.00232 |
| 105 | Gobio gobio gobio | 17 | 12 | 0.00972 |
| 106 | Gobionotothen gibberifrons | 470 | 0.25 | 0.04206 |
| 107 | Gobius paganellus | 10.5 | 23.1 | 0.00894 |
| 108 | Gymnocephalus cernuus | 64.8 | 17 | 0.0358 |
| 109 | Gymnodraco acuticeps | 87.2 | -0.3 | 0.01188 |
| 110 | Gymnoscopelus braueri | 11.4 | 0.25 | 0.00168 |
| 111 | Gymnoscopelus opisthopterus | 23.55 | 0.25 | 0.00293 |
| 112 | Harpagifer georgianus | 4.13 | 1.5 | 3.22E-05 |
| 113 | Hemichromis bimaculatus | 3 | 25 | 0.00225 |
| 114 | Heteropneustes fossilis | 45 | 18 | 0.00648 |
| 115 | Hippocampus hippocampus | 10 | 18 | 0.00502 |
| 116 | Hippoglossoides platessoides | 390 | 3.5 | 0.02578 |
| 117 | Hoplerythrinus unitaeniatus | 243 | 28.5 | 0.03686 |
| 118 | Ichthyomyzon fossor | 3.78 | 18 | 0.00225 |
| 119 | Ictalurus punctatus | 825 | 18 | 0.13481 |
| 120 | Katsuwonus pelamis | 632 | 23.5 | 0.51628 |
| 121 | Kuhlia sandvicensis | 55.97 | 23 | 0.01285 |
| 122 | Labeo calbasu | 0.3 | 27.5 | 6.65E-05 |
| 123 | Labeo capensis | 357.96 | 8 | 0.0543 |
| 124 | Labeo rohita | 5 | 29 | 0.00467 |
| 125 | Labeobarbus aeneus | 325.5 | 10 | 0.02152 |
| 126 | Labrus bergylta | 125 | 18 | 0.04134 |
| 127 | Lagodon rhomboides | 13.5 | 25 | 0.00305 |
| 128 | Lampetra fluviatilis | 1.43 | 4.4 | 9.45E-05 |
| 129 | Lampetra planeri | 2.79 | 5.3 | 1.52E-04 |
| 130 | Leiostomus xanthurus | 11.1 | 25 | 0.00242 |
| 131 | Lepidocephalichthys guntea | 1.27 | 24 | 6.92E-04 |
| 132 | Lepidogalaxias salamandroides | 0.62 | 20 | 1.30E-04 |
| 133 | Lepomis cyanellus | 10 | 15 | 0.00506 |
| 134 | Lepomis gibbosus | 30 | 5 | 0.00268 |
| 135 | Lepomis macrochirus | 133 | 30 | 0.02587 |
| 136 | Leporinus fasciatus | 3.95 | 25 | 0.00214 |
| 137 | Leucaspius delineatus | 1.52 | 20 | 0.0026 |
| 138 | Leuciscus cephalus | 14 | 15 | 0.0061 |
| 139 | Leuciscus idus | 600 | 5 | 0.04668 |
| 140 | Leuciscus leuciscus | 9.95 | 16.5 | 0.01393 |
| 141 | Limanda limanda | 400 | 5 | 0.02336 |
| 142 | Lipolagus ochotensis | 3.4 | 10 | 0.00159 |
| 143 | Lipophrys pholis | 20.2 | 16 | 0.00707 |
| 144 | Liza dumerili | 42.5 | 18 | 0.01157 |
| 145 | Liza macrolepis | 8 | 29 | 0.00467 |
| 146 | Liza richardsonii | 39.2 | 13 | 0.01967 |
| 147 | Lota lota | 213 | 11.3 | 0.06132 |
| 148 | Lutjanus campechanus | 365.5 | 15 | 0.1038 |
| 149 | Lycodichthys dearborni | 44.61 | -1.5 | 0.00139 |
| 150 | Macrognathus aculeatus | 53 | 21 | 0.0101 |
| 151 | Melamphaes acanthomus | 17.4 | 5 | 0.00122 |
| 152 | Melanocetus johnsonii | 50.55 | 2.5 | 0.00472 |
| 153 | Melanogrammus aeglefinus | 155.9 | 10 | 0.02063 |
| 154 | Melanonus zugmayeri | 31.5 | 5 | 0.00306 |
| 155 | Melanostigma gelatinosum | 47.2 | 0.25 | 0.00551 |
| 156 | Melanostigma pammelas | 10 | 3 | 3.89E-04 |
| 157 | Micropterus salmoides | 178 | 15 | 0.01315 |
| 158 | Microstomus kitt | 229 | 5 | 0.02583 |
| 159 | Misgurnus fossilis | 28.5 | 18 | 0.01364 |
| 160 | Monopterus cuchia | 172.88 | 25 | 0.0195 |
| 161 | Mugil cephalus | 221 | 14.5 | 0.04471 |
| 162 | Mugil curema | 140 | 20 | 0.05446 |
| 163 | Myoxocephalus octodecemspinosus | 200 | 10 | 0.03112 |
| 164 | Myoxocephalus scorpius | 87.5 | 2.4 | 0.01464 |
| 165 | Mystus armatus | 9.2 | 30 | 0.00555 |
| 166 | Mystus cavasius | 45 | 29 | 0.03168 |
| 167 | Mystus gulio | 12 | 27 | 0.00728 |
| 168 | Mystus vittatus | 7.4 | 20 | 0.00314 |
| 169 | Myxine glutinosa | 38.4 | 7 | 0.00717 |
| 170 | Nannobrachium regale | 2.9 | 5 | 1.80E-04 |
| 171 | Nannobrachium ritteri | 1.8 | 5 | 4.13E-04 |
| 172 | Naucrates ductor | 0.07 | 23 | 5.63E-04 |
| 173 | Notothenia coriiceps | 1000 | 0.25 | 0.1128 |
| 174 | Notothenia cyanobrancha | 200 | 4.5 | 0.08558 |
| 175 | Notothenia rossii | 159.7 | 3 | 0.01615 |
| 176 | Oligolepis acutipennis | 6.3 | 26 | 7.60E-04 |
| 177 | Oncorhynchus mykiss | 450 | 5 | 0.06827 |
| 178 | Oncorhynchus nerka | 36.7 | 5 | 0.00585 |
| 179 | Oncorhynchus tshawytscha | 26 | 16 | 0.0174 |
| 180 | Oneirodes acanthias | 4.2 | 5 | 1.96E-04 |
| 181 | Ophiodon elongatus | 1591 | 12.1 | 0.1548 |
| 182 | Opsanus tau | 325 | 20 | 0.5057 |
| 183 | Oreochromis aureus | 697 | 26 | 0.17892 |
| 184 | Oreochromis mossambicus | 144.3 | 16 | 0.02076 |
| 185 | Oreochromis niloticus niloticus | 310 | 26 | 0.06873 |
| 186 | Orthodon microlepidotus | 650 | 12 | 0.08092 |
| 187 | Oryzias latipes | 0.27 | 25 | 1.94E-04 |
| 188 | Osphronemus goramy | 12.5 | 28 | 0.00433 |
| 189 | Pagetopsis macropterus | 76 | 0 | 0.0071 |
| 190 | Pagothenia borchgrevinki | 90.15 | 0.5 | 0.01052 |
| 191 | Paranotothenia magellanica | 200 | 5.25 | 0.07314 |
| 192 | Parophrys vetulus | 70 | 15 | 0.02723 |
| 193 | Parvilux ingens | 9.4 | 5 | 6.58E-04 |
| 194 | Perca fluviatilis | 11 | 16 | 9.85E-04 |
| 195 | Petromyzon marinus | 22.8 | 5 | 0.0047 |
| 196 | Pimephales promelas | 2 | 15 | 8.87E-04 |
| 197 | Platichthys flesus | 350 | 9 | 0.01635 |
| 198 | Platichthys stellatus | 1290 | 9 | 0.08024 |
| 199 | Plecoglossus altivelis altivelis | 10.7 | 19 | 0.03334 |
| 200 | Pleuronectes platessa | 288.6 | 5 | 0.02245 |
| 201 | Poecilia latipinna | 5.6 | 25 | 7.41E-04 |
| 202 | Pollachius pollachius | 870 | 18.5 | 0.81223 |
| 203 | Pomadasys commersonnii | 2627 | 15 | 0.45999 |
| 204 | Pomoxis annularis | 11.5 | 17.4 | 0.00407 |
| 205 | Poromitra crassiceps | 17.1 | 5 | 0.00106 |
| 206 | Protopterus annectens annectens | 368 | 30 | 0.11452 |
| 207 | Psenes whiteleggii | 1.3 | 13.5 | 0.00329 |
| 208 | Psetta maxima | 320 | 15 | 0.14192 |
| 209 | Pseudobathylagus milleri | 41.1 | 5 | 0.00256 |
| 210 | Pseudochaenichthys georgianus | 35.7 | 0.5 | 0.00555 |
| 211 | Pseudopleuronectes americanus | 26.15 | 12 | 4.08E-04 |
| 212 | Pterophyllum scalare | 19.03 | 25 | 0.00585 |
| 213 | Rhinogobiops nicholsii | 4.04 | 15 | 4.87E-04 |
| 214 | Rhodeus amarus | 0.71 | 16 | 5.36E-04 |
| 215 | Rhodeus sericeus | 3 | 12 | 0.00214 |
| 216 | Rutilus rutilus | 27 | 8 | 0.00788 |
| 217 | Sagamichthys abei | 5.7 | 5 | 5.10E-04 |
| 218 | Salmo salar | 25 | 7 | 0.00564 |
| 219 | Salmo trutta fario | 350 | 10 | 0.07896 |
| 220 | Salmo trutta trutta | 575 | 10 | 0.09844 |
| 221 | Salvelinus alpinus alpinus | 210 | 15 | 0.06535 |
| 222 | Salvelinus fontinalis | 690 | 10 | 0.05906 |
| 223 | Salvelinus namaycush | 82.8 | 9.2 | 0.00741 |
| 224 | Sander lucioperca | 600 | 5 | 0.07002 |
| 225 | Sander vitreus | 291 | 20 | 0.07359 |
| 226 | Sarda chiliensis lineolata | 2530 | 22 | 16.96719 |
| 227 | Sarotherodon galilaeus galilaeus | 283 | 26 | 0.07706 |
| 228 | Scardinius erythrophthalmus | 53 | 20 | 0.04474 |
| 229 | Scopelengys tristis | 49.8 | 5 | 0.00252 |
| 230 | Scopelogadus mizolepis mizolepis | 3.6 | 5 | 2.80E-04 |
| 231 | Scophthalmus rhombus | 145 | 18.5 | 0.07333 |
| 232 | Scorpaena porcus | 50 | 20 | 0.01283 |
| 233 | Scyliorhinus canicula | 857 | 7 | 0.07336 |
| 234 | Scyliorhinus stellaris | 2530 | 18.3 | 0.03947 |
| 235 | Sebastes diploproa | 2 | 10 | 9.10E-04 |
| 236 | Sebastolobus altivelis | 198 | 5.7 | 0.00309 |
| 237 | Seriola quinqueradiata | 989 | 19.2 | 0.56937 |
| 238 | Serranus scriba | 4.1 | 16 | 0.00265 |
| 239 | Solea solea | 185 | 14 | 0.07557 |
| 240 | Sparus aurata | 78 | 18 | 0.0616 |
| 241 | Spinachia spinachia | 0.3 | 18 | 4.13E-04 |
| 242 | Squalus acanthias | 1812 | 9 | 0.14804 |
| 243 | Stenobrachius leucopsarus | 4.3 | 5 | 0.001 |
| 244 | Stomias atriventer | 9.3 | 10 | 0.0021 |
| 245 | Stomias danae | 13.8 | 2.5 | 0.00225 |
| 246 | Symbolophorus californiensis | 0.8 | 5 | 2.68E-04 |
| 247 | Synbranchus marmoratus | 151 | 25 | 0.02114 |
| 248 | Syngnathus acus | 7.6 | 18.5 | 0.00574 |
| 249 | Tarletonbeania crenularis | 3.29 | 8 | 0.00175 |
| 250 | Tautogolabrus adspersus | 50 | 20 | 0.02762 |
| 251 | Theragra chalcogramma | 70 | 1 | 0.00899 |
| 252 | Thorichthys meeki | 15 | 25 | 0.00479 |
| 253 | Thymallus arcticus arcticus | 283 | 4 | 0.07485 |
| 254 | Tilapia rendalli | 50 | 17 | 0.01634 |
| 255 | Tilapia zillii | 315 | 26 | 0.07229 |
| 256 | Tinca tinca | 541 | 16 | 0.13049 |
| 257 | Torpedo marmorata | 448 | 16 | 0.03835 |
| 258 | Torpedo torpedo | 315 | 15 | 0.07966 |
| 259 | Trematomus bernacchii | 55.2 | 0.1 | 0.00279 |
| 260 | Trematomus hansoni | 145 | -0.9 | 0.03722 |
| 261 | Trematomus pennellii | 183 | -0.9 | 0.03133 |
| 262 | Trichogaster trichopterus | 7.97 | 27 | 0.00518 |
| 263 | Triphoturus mexicanus | 1.15 | 5 | 1.61E-04 |
| 264 | Typhlogobius californiensis | 3.54 | 15 | 2.76E-05 |
| 265 | Xiphophorus hellerii | 2 | 25 | 0.00167 |
|  |  |  |  |  |

**Table S3d: Standard metabolic rates in birds**[**^2^**](#_ENREF_2)**^,^**[**^3^**](#_ENREF_3)**.**

| Number | Species | Body mass (gram) | T (^o^C) | Basal metabolic rate (watts) |
| --- | --- | --- | --- | --- |
| 1 | Acanthis cannabina | 20.1 | 16.9 | 0.33969 |
| 2 | Acanthis flammea | 20.4 | 14.0 | 0.2856 |
| 3 | Acanthorhynchus tenuirostris | 25.7 | 9.7 | 0.24929 |
| 4 | Accipiter nisus | 7.0 | 135 | 0.945 |
| 5 | Acrcocephalus palustris | 18.9 | 10.8 | 0.20412 |
| 6 | Acridotheres cristatellus | 11.0 | 109.4 | 1.2034 |
| 7 | Acrocephalus arundinaceus | 11.7 | 21.9 | 0.25623 |
| 8 | Acrocephalus bistrigiceps | 16.6 | 7.9 | 0.13114 |
| 9 | Acrocephalus palustris | 18.8 | 10.8 | 0.20304 |
| 10 | Acrocephalus schoenobaenus | 18.9 | 11.5 | 0.21735 |
| 11 | Aegithalos caudatus | 22.4 | 8.9 | 0.19936 |
| 12 | Aegolius acadicus | 5.3 | 124 | 0.6572 |
| 13 | Aethopyga christinae | 22.7 | 5.2 | 0.11804 |
| 14 | Agapornis fisheri | 9.3 | 56.7 | 0.52731 |
| 15 | Agapornis roseicollis | 9.6 | 48.4 | 0.46464 |
| 16 | Agelaius phoeniceus | 14.7 | 56.7 | 0.83349 |
| 17 | Agleactis cupripennis | 35.0 | 7.2 | 0.252 |
| 18 | Aix sponsa | 5.0 | 448 | 2.24 |
| 19 | Alaemon alaudipes | 11.3 | 37.7 | 0.42601 |
| 20 | Alauda arvensis | 22.8 | 31.7 | 0.72276 |
| 21 | Alcedo atthis | 11.0 | 34.3 | 0.3773 |
| 22 | Alectoris graeca | 4.0 | 633 | 2.532 |
| 23 | Alophoixus bres | 10.1 | 35 | 0.3535 |
| 24 | Amadina erythrocephala | 9.5 | 22.4 | 0.2128 |
| 25 | Amadina fasciata | 12.4 | 17.2 | 0.21328 |
| 26 | Ammodramus savannarum | 12.9 | 13.8 | 0.17802 |
| 27 | Amphispiza bilineata | 17.0 | 11.6 | 0.1972 |
| 28 | Anas acuta | 6.1 | 721 | 4.3981 |
| 29 | Anas penelope | 3.9 | 723 | 2.8197 |
| 30 | Anas platyrhynchos | 4.0 | 1020 | 4.08 |
| 31 | Anas strepera | 7.8 | 791 | 6.1698 |
| 32 | Anhinga rufa (anhinga) | 3.1 | 1040 | 3.224 |
| 33 | Anser anser | 3.3 | 3250 | 10.725 |
| 34 | Anthracothorax nigricollis | 39.6 | 7.7 | 0.30492 |
| 35 | Anthus campestris | 17.6 | 21.8 | 0.38368 |
| 36 | Anthus pratensis | 15.9 | 18.9 | 0.30051 |
| 37 | Anthus trivialis | 17.2 | 19.7 | 0.33884 |
| 38 | Aptenodytes patagonica | 2.0 | 11080 | 22.16 |
| 39 | Apteryx australis | 1.7 | 2380 | 4.046 |
| 40 | Apteryx haasti | 1.7 | 2450 | 4.165 |
| 41 | Apteryx owenii | 1.9 | 1096 | 2.0824 |
| 42 | Apus apus | 9.7 | 44.9 | 0.43553 |
| 43 | Arachnothera longirostra | 14.5 | 13 | 0.1885 |
| 44 | Ardea herodias | 3.3 | 1870 | 6.171 |
| 45 | Arenaria interpres | 10.2 | 90 | 0.918 |
| 46 | Asio flammeus | 3.2 | 406 | 1.2992 |
| 47 | Asio otus | 3.8 | 252 | 0.9576 |
| 48 | Authus pratensis | 15.9 | 18.9 | 0.30051 |
| 49 | Barnardius zonarius | 5.2 | 137 | 0.7124 |
| 50 | Bombycilla garrulus | 13.2 | 72.5 | 0.957 |
| 51 | Bonasa umbellus | 3.7 | 644 | 2.3828 |
| 52 | Botaurus lentigosus | 4.5 | 600 | 2.7 |
| 53 | Bubo virginianus | 3.6 | 1450 | 5.22 |
| 54 | Buteo buteo | 3.7 | 1012 | 3.7444 |
| 55 | Buteo lineatus | 3.2 | 658 | 2.1056 |
| 56 | Cacactua tenuirostris | 5.8 | 549.9 | 3.18942 |
| 57 | Cacatua galerita | 4.4 | 776.1 | 3.41484 |
| 58 | Cacomantis variolosus | 5.1 | 23.8 | 0.12138 |
| 59 | Calidris canutus | 6.8 | 130 | 0.884 |
| 60 | Callipepla gambelii | 6.0 | 126.1 | 0.7566 |
| 61 | Caprimulgus europeus | 8.3 | 77.4 | 0.64242 |
| 62 | Cardinalis cardinalis | 12.3 | 41 | 0.5043 |
| 63 | Cardinalis sinuata | 12.3 | 32 | 0.3936 |
| 64 | Carduelis carduelis | 21.1 | 16.5 | 0.34815 |
| 65 | Carduelis pinus | 20.8 | 14 | 0.2912 |
| 66 | Carduelis tristis | 24.6 | 13.6 | 0.33456 |
| 67 | Carpodacus cassinii | 12.4 | 27.4 | 0.33976 |
| 68 | Carpodacus erythrinus | 16.6 | 21.6 | 0.35856 |
| 69 | Carpodacus mexicanus | 15.2 | 20.4 | 0.31008 |
| 70 | Casurarius bennetti | 1.4 | 17600 | 24.64 |
| 71 | Catharactus skua | 4.9 | 970 | 4.753 |
| 72 | Centropus senegalensis | 8.6 | 175 | 1.505 |
| 73 | Certhilauda erythrochlamys | 15.1 | 27.3 | 0.41223 |
| 74 | Chalcophaps inidica | 6.4 | 124 | 0.7936 |
| 75 | Charadius dubius | 10.9 | 44 | 0.4796 |
| 76 | Chauna chavaria | 2.6 | 2620 | 6.812 |
| 77 | Chloris chloris | 16.8 | 28.2 | 0.47376 |
| 78 | Chloropsis sonnerati | 9.5 | 39.7 | 0.37715 |
| 79 | Chlorostilbon mellisugus | 50.0 | 2.9 | 0.145 |
| 80 | Chordeiles minor | 6.1 | 72 | 0.4392 |
| 81 | Cinclus mexicanus | 9.2 | 50.2 | 0.46184 |
| 82 | Coccothraustes coccothraustes | 14.5 | 48.3 | 0.70035 |
| 83 | Coereba flaveola | 21.5 | 10 | 0.215 |
| 84 | Coleus monedula | 7.3 | 209.0 | 1.5257 |
| 85 | Colinus virginianus | 5.7 | 194 | 1.1058 |
| 86 | Colius castanotus | 15.0 | 69 | 1.035 |
| 87 | Colius colius | 5.0 | 35.1 | 0.1755 |
| 88 | Colius striatus | 4.6 | 51 | 0.2346 |
| 89 | Columba leucomela | 5.3 | 456 | 2.4168 |
| 90 | Columba livia | 4.5 | 368 | 1.656 |
| 91 | Columba palumbus | 4.0 | 493 | 1.972 |
| 92 | Columba unicincta | 5.4 | 318 | 1.7172 |
| 93 | Contopus virens | 18.5 | 13.9 | 0.25715 |
| 94 | Copsychus saularis | 6.9 | 33.5 | 0.23115 |
| 95 | Cornus ruficollis | 5.1 | 660.0 | 3.366 |
| 96 | Corvus brachyrhynchos | 8.5 | 384.8 | 3.2708 |
| 97 | Corvus corax | 4.6 | 1203.0 | 5.5338 |
| 98 | Corvus corone cornix | 6.4 | 518.0 | 3.3152 |
| 99 | Corvus frugilegus | 6.7 | 390.0 | 2.613 |
| 100. | Coturnix chinensis | 8.2 | 44.9 | 0.36818 |
| 101. | Coturnix coturnix | 7.6 | 109 | 0.8284 |
| 102. | Coturnix japonica | 8.5 | 115 | 0.9775 |
| 103. | Coturnix pectoralis | 6.6 | 95.8 | 0.63228 |
| 104. | Crax alberti | 2.4 | 2800 | 6.72 |
| 105. | Crax daubentoni | 2.6 | 2800 | 7.28 |
| 106. | Crex crex | 8.2 | 96 | 0.7872 |
| 107. | Cuculus canorus | 7.5 | 111.6 | 0.837 |
| 108. | Cyanocitta cristata | 10.3 | 80.8 | 0.83224 |
| 109. | Cygnus buccinator | 2.3 | 8800 | 20.24 |
| 110. | Dendragapus obscurus | 4.4 | 1131 | 4.9764 |
| 111. | Dendrocopus major | 8.9 | 117.0 | 1.0413 |
| 112. | Dendroica coronata | 16.4 | 11.5 | 0.1886 |
| 113. | Dendroica dominica | 16.3 | 9.8 | 0.15974 |
| 114. | Dendroica palmarum | 15.8 | 9.8 | 0.15484 |
| 115. | Dendroica pinus | 14.9 | 12 | 0.1788 |
| 116. | Diomedea chrysostoma | 2.3 | 3753 | 8.6319 |
| 117 | Diomedea exulans | 2.5 | 8130 | 20.325 |
| 118 | Diomedea immutabilis | 3 | 2522 | 7.566 |
| 119 | Emberiza citrinella | 16.3 | 26.8 | 0.43684 |
| 120 | Emberiza hortulana | 15.1 | 27 | 0.4077 |
| 121 | Emberiza schoeniclus | 17.1 | 17.6 | 0.30096 |
| 122 | Empidonax virescens | 14.6 | 12.3 | 0.17958 |
| 123 | Eolophus roseicapillus | 4.6 | 268.7 | 1.23602 |
| 124 | Eremalauda dunni | 13.5 | 20.6 | 0.2781 |
| 125 | Eremophila alpestris | 11.9 | 26 | 0.3094 |
| 126 | Erithacus rubecula | 16 | 17.6 | 0.2816 |
| 127 | Estrilda melpoda | 17.5 | 7.5 | 0.13125 |
| 128 | Estrilda troglodytes | 20.1 | 7.5 | 0.15075 |
| 129 | Eudocimus albus (Guara alba) | 4.4 | 940 | 4.136 |
| 130 | Eudynamys scolopacea | 8.8 | 188 | 1.6544 |
| 131 | Eudyptes chrysolophus | 2.2 | 3870 | 8.514 |
| 132 | Eudyptes cristatus | 2.5 | 2330 | 5.825 |
| 133 | Eudyptula minor | 3.9 | 960 | 3.744 |
| 134 | Eurostopodus argus | 4.6 | 88 | 0.4048 |
| 135 | Excalfactoria chinensis | 9.2 | 44 | 0.4048 |
| 136 | Falco sparverius | 7.2 | 117 | 0.8424 |
| 137 | Falco subbuteo | 6.2 | 208 | 1.2896 |
| 138 | Falco tinnunculus | 5.9 | 131 | 0.7729 |
| 139 | Ficedula hypoleuca | 19.9 | 11.7 | 0.23283 |
| 140 | Fregata magnificens | 2.6 | 1078 | 2.8028 |
| 141 | Fringilla coelebs | 17.7 | 21 | 0.3717 |
| 142 | Fringilla montifringilla | 18.2 | 21 | 0.3822 |
| 143 | Fulica atra | 5 | 412 | 2.06 |
| 144 | Gallus gallus | 2.2 | 2710 | 5.962 |
| 145 | Garrulus glandarius | 9.1 | 153 | 1.3923 |
| 146 | Geococcyx californianus | 5.1 | 284.7 | 1.45197 |
| 147 | Geopelia cuneata | 6.8 | 39 | 0.2652 |
| 148 | Geopelia placida | 6.8 | 52 | 0.3536 |
| 149 | Geophaps plumifera | 4.9 | 81 | 0.3969 |
| 150 | Geophaps smithii | 4.4 | 198 | 0.8712 |
| 151 | Glaucidium cuculoides | 5.3 | 163 | 0.8639 |
| 152 | Glaucidium gnoma | 8.2 | 54 | 0.4428 |
| 153 | Grus canadensis | 2.1 | 3890 | 8.169 |
| 154 | Grus paradisea | 2.6 | 4030 | 10.478 |
| 155 | Gypaetus barbatus | 2.2 | 5070 | 11.154 |
| 156 | Haematopus ostralegus | 5.3 | 554 | 2.9362 |
| 157 | Himatione sanguinea | 22.2 | 13.5 | 0.2997 |
| 158 | Hippolais icterina | 20.2 | 12.5 | 0.2525 |
| 159 | Hirundo rustica | 16.4 | 18.4 | 0.30176 |
| 160 | Hirundo tahitica | 12.7 | 14.1 | 0.17907 |
| 161 | Icterus galbula | 13.4 | 37.5 | 0.5025 |
| 162 | Jabiru mycteria | 2.4 | 5470 | 13.128 |
| 163 | Junco hyemalis | 16.4 | 18 | 0.2952 |
| 164 | Lagopus lagopus | 5.1 | 567 | 2.8917 |
| 165 | Lagopus leucurus | 7.2 | 326 | 2.3472 |
| 166 | Lanius collurio | 14.2 | 27 | 0.3834 |
| 167 | Lanius excubitor | 11.2 | 72.4 | 0.81088 |
| 168 | Larus argentatus | 4.8 | 1000 | 4.8 |
| 169 | Larus atricilla | 6.8 | 275.6 | 1.87408 |
| 170 | Larus canus | 5.2 | 431 | 2.2412 |
| 171 | Larus ridibundus | 6.1 | 306 | 1.8666 |
| 172 | Leptoptilos javanicus | 2.6 | 5710 | 14.846 |
| 173 | Leptotila verreauxi | 6.8 | 131 | 0.8908 |
| 174 | Leucosarcia melanoleuca | 3.8 | 445 | 1.691 |
| 175 | Lichenostomus virescens | 14.2 | 25 | 0.355 |
| 176 | Lichmera indistincta | 23.1 | 9 | 0.2079 |
| 177 | Lonchura fuscans | 10.2 | 9.5 | 0.0969 |
| 178 | Lonchura maja | 11.7 | 12.8 | 0.14976 |
| 179 | Lonchura malacca | 11.9 | 11.8 | 0.14042 |
| 180 | Lonchura striata | 19.7 | 10.1 | 0.19897 |
| 181 | Loxia curvirostra | 15.2 | 39.4 | 0.59888 |
| 182 | Loxia pytiopsittacus | 14.9 | 53.7 | 0.80013 |
| 183 | Loxoides baileui | 12.9 | 36 | 0.4644 |
| 184 | Lullula arborea | 14.7 | 33.2 | 0.48804 |
| 185 | Luscinia svecica | 17.3 | 20.8 | 0.35984 |
| 186 | Macronectes giganteus | 2.8 | 4780 | 13.384 |
| 187 | Malacopteron cinereum | 13.5 | 15.8 | 0.2133 |
| 188 | Manacus vitellinus | 15 | 15.5 | 0.2325 |
| 189 | Megadyptes antipodes | 2.4 | 4800 | 11.52 |
| 190 | Meitihreptus lunatus | 17.4 | 14.3 | 0.24882 |
| 191 | Melanerpes formicivorus | 10.1 | 73 | 0.7373 |
| 192 | Melopsittacus undulatus | 9.8 | 33.6 | 0.32928 |
| 193 | Melospiza georgiana | 14.2 | 14.9 | 0.21158 |
| 194 | Melospiza melodia | 13.1 | 19.1 | 0.25021 |
| 195 | Merops viridis | 8.7 | 33.8 | 0.29406 |
| 196 | Motacilla alba | 15.5 | 18.2 | 0.2821 |
| 197 | Motacilla flava | 17.5 | 14.7 | 0.25725 |
| 198 | Muscicapa striata | 17.1 | 14.4 | 0.24624 |
| 199 | Myiarchus crinitus | 11.3 | 33.9 | 0.38307 |
| 200 | Nectarinia venusta | 19.7 | 7.1 | 0.13987 |
| 201 | Neophema petrophila | 13.1 | 48.4 | 0.63404 |
| 202 | Nucifraga caryocatactes | 9.2 | 147 | 1.3524 |
| 203 | Nyctea scandiaca | 2.1 | 2026 | 4.2546 |
| 204 | Nymphicus hollandicus | 8 | 85.6 | 0.6848 |
| 205 | Oceanodroma furcata | 10.1 | 44.6 | 0.45046 |
| 206 | Ocyphaps lophotes | 5.8 | 187 | 1.0846 |
| 207 | Oreotrochilus estella | 22.9 | 8.4 | 0.19236 |
| 208 | Oriolus oriolus | 10 | 64.9 | 0.649 |
| 209 | Otus asio | 3.5 | 166 | 0.581 |
| 210 | Otus trichopsis | 3.7 | 120 | 0.444 |
| 211 | Padda oryzivora | 12.1 | 25.4 | 0.30734 |
| 212 | Parus ater | 22 | 10.8 | 0.2376 |
| 213 | Parus atricapillus | 24.5 | 10.3 | 0.25235 |
| 214 | Parus major | 20.1 | 16.4 | 0.32964 |
| 215 | Parus varius | 20.3 | 17.7 | 0.35931 |
| 216 | Passer domesticus bactrianus | 15.9 | 23.2 | 0.36888 |
| 217 | Passer montanus | 17.9 | 22 | 0.3938 |
| 218 | Passerculus sandwichensis | 13.9 | 15.9 | 0.22101 |
| 219 | Patagona gigas | 15 | 19.1 | 0.2865 |
| 220 | Pelacanoides urinatrix | 10.3 | 136 | 1.4008 |
| 221 | Pelecanus conspicullatus | 3.6 | 5090 | 18.324 |
| 222 | Pelecanus occidentalis | 3.4 | 3038 | 10.3292 |
| 223 | Penelope purpurescens | 2.7 | 2040 | 5.508 |
| 224 | Perdix perdix | 4.3 | 501 | 2.1543 |
| 225 | Perisoreus canadensis | 9.5 | 71.2 | 0.6764 |
| 226 | Pernis apivorus | 3.6 | 652 | 2.3472 |
| 227 | Phalacrocorax auritus | 4.1 | 1330 | 5.453 |
| 228 | Phalaenoptilus nuttalli | 4.4 | 35 | 0.154 |
| 229 | Phaps chalcoptera | 5 | 304 | 1.52 |
| 230 | Phaps elegans | 6.5 | 190 | 1.235 |
| 231 | Phaps histrionica | 5 | 257 | 1.285 |
| 232 | Philidonyris novaehollandiae | 18.3 | 17.3 | 0.31659 |
| 233 | Phoenicopterus ruber | 5 | 3040 | 15.2 |
| 234 | Phoeniculus purpureus | 2.3 | 74.07 | 0.17036 |
| 235 | Phoenicurus ochruros | 17.4 | 13.9 | 0.24186 |
| 236 | Phoenicurus phoenicurus | 17.9 | 13 | 0.2327 |
| 237 | Phylidonyris melanops | 15.6 | 18.8 | 0.29328 |
| 238 | Phylloscopus collybita | 20 | 8.2 | 0.164 |
| 239 | Phylloscopus sibilatrix | 19 | 9.2 | 0.1748 |
| 240 | Phylloscopus trochilus | 19.5 | 10.7 | 0.20865 |
| 241 | Pica nuttalli | 9.7 | 151.9 | 1.47343 |
| 242 | Pica pica | 7.5 | 158.9 | 1.19175 |
| 243 | Picoides major | 8.9 | 117 | 1.0413 |
| 244 | Picoides pubescens | 17.7 | 21.7 | 0.38409 |
| 245 | Pinicola enucleator | 13.8 | 78.4 | 1.08192 |
| 246 | Pipra mentalis | 15.8 | 12.3 | 0.19434 |
| 247 | Pluvialis dominica | 5.5 | 118 | 0.649 |
| 248 | Pluvialis squatarola | 7.9 | 226 | 1.7854 |
| 249 | Podargus ocellatus | 3.9 | 145 | 0.5655 |
| 250 | Podargus strigoides | 2.7 | 380.3 | 1.02681 |
| 251 | Pooectes gramineus | 12.6 | 21.5 | 0.2709 |
| 252 | Protonotaria citrea | 15.5 | 12.8 | 0.1984 |
| 253 | Prunella modularls | 19.4 | 16.8 | 0.32592 |
| 254 | Psaltriparus minimus | 22 | 5.5 | 0.121 |
| 255 | Pterocles orientalis | 5 | 386.4 | 1.932 |
| 256 | Pterodroma phaeopygia | 12.4 | 425 | 5.27 |
| 257 | Ptilinopus melanospila | 5 | 98 | 0.49 |
| 258 | Ptilinopus superbus | 6.3 | 120.4 | 0.75852 |
| 259 | Puffinus griseus | 3.9 | 740 | 2.886 |
| 260 | Pycnonotus finlaysoni | 8.4 | 26.3 | 0.22092 |
| 261 | Pycnonotus goiavier | 8.6 | 28.6 | 0.24596 |
| 262 | Pygoscelis adeliae | 3.1 | 3970 | 12.307 |
| 263 | Pygoscelis papua | 3 | 6290 | 18.87 |
| 264 | Pyrrhula pyrrhula | 18.2 | 30.4 | 0.55328 |
| 265 | Regulus regulus | 26.5 | 5.5 | 0.14575 |
| 266 | Riparia riparia | 17.1 | 13.6 | 0.23256 |
| 267 | Saxicola rubetra | 16.9 | 14.3 | 0.24167 |
| 268 | Sayornis phoebe | 15.9 | 21.6 | 0.34344 |
| 269 | Scardefella inca | 6.2 | 40.5 | 0.2511 |
| 270 | Scolopax minor | 6.8 | 156.7 | 1.06556 |
| 271 | Scolopax rusticola | 5 | 430 | 2.15 |
| 272 | Sephanoides sephaniodes | 17.8 | 5.74 | 0.10217 |
| 273 | Serinus canaria | 17.1 | 13.3 | 0.22743 |
| 274 | Sialia mexicana | 15.4 | 27.5 | 0.4235 |
| 275 | Spermestes cucullatus | 7.3 | 10.62 | 0.07753 |
| 276 | Spheniscus humboldti | 2.5 | 3870 | 9.675 |
| 277 | Spinus spinus | 20.8 | 14 | 0.2912 |
| 278 | Spizella arborea | 19.8 | 16.6 | 0.32868 |
| 279 | Spizella passerina | 16.3 | 11.9 | 0.19397 |
| 280 | Sterna maxima | 6.7 | 373 | 2.4991 |
| 281 | Streptopelia senegalensis | 7.9 | 108 | 0.8532 |
| 282 | Streptopelia turtur | 7.4 | 154 | 1.1396 |
| 283 | Strix aluco | 4 | 520 | 2.08 |
| 284 | Strix occidentalis | 4.7 | 571 | 2.6837 |
| 285 | Struthio camelus | 0.6 | 100000 | 60 |
| 286 | Sturnus vulgaris | 12 | 75 | 0.9 |
| 287 | Sula dactylatra | 4.3 | 1289 | 5.5427 |
| 288 | Sylvia atricapilla | 19 | 21.9 | 0.4161 |
| 289 | Sylvia borin | 16.8 | 24.8 | 0.41664 |
| 290 | Sylvia curruca | 18.8 | 10.6 | 0.19928 |
| 291 | Sylvia nisoria | 15.1 | 21.4 | 0.32314 |
| 292 | Taeniopygia castanotis | 19.5 | 11.7 | 0.22815 |
| 293 | Tarsiger cyanurus | 16 | 14.8 | 0.2368 |
| 294 | Tetrao urogallus | 2.9 | 4010 | 11.629 |
| 295 | Thamnophilus punctatus | 16.4 | 21 | 0.3444 |
| 296 | Thinocorus rumicivorus | 5.6 | 55.5 | 0.3108 |
| 297 | Tiaris canora | 19.9 | 7.8 | 0.15522 |
| 298 | Tringa ochropus | 10.2 | 90 | 0.918 |
| 299 | Troglodytes troglodytes | 23.7 | 9 | 0.2133 |
| 300 | Trogon rufus | 8.1 | 53 | 0.4293 |
| 301 | Tudus viscivorus | 10.2 | 108.2 | 1.10364 |
| 302 | Turdus iliacus | 12.5 | 58 | 0.725 |
| 303 | Turdus merula | 11.3 | 82.6 | 0.93338 |
| 304 | Turdus philomelos | 11.6 | 62.8 | 0.72848 |
| 305 | Turdus viscivorus | 10.2 | 108.2 | 1.10364 |
| 306 | Turnix suscitator | 6.7 | 58.1 | 0.38927 |
| 307 | Tyrannus tyrannus | 12.2 | 35.7 | 0.43554 |
| 308 | Upupa epops | 8.2 | 67 | 0.5494 |
| 309 | Uraeginthus bengalis | 17 | 9.1 | 0.1547 |
| 310 | Uria aalge | 7.1 | 956 | 6.7876 |
| 311 | Uria lomvia | 6.9 | 989 | 6.8241 |
| 312 | Vermivora pinus | 19.2 | 7.8 | 0.14976 |
| 313 | Vidua paradisaea | 18.5 | 10.5 | 0.19425 |
| 314 | Vultur gryphus | 1.6 | 10320 | 16.512 |
| 315 | Xiphorhynchus guttatus | 9.9 | 45.2 | 0.44748 |
| 316 | Yynx torquilla | 11.3 | 31.8 | 0.35934 |
| 317 | Zenaida macroura | 6 | 123 | 0.738 |
| 318 | Zonotricha querula | 13.4 | 33.3 | 0.44622 |
| 319 | Zonotrichia albicollis | 13.8 | 20.2 | 0.27876 |
| 320 | Zonotrichia leucophrys | 12.9 | 26.1 | 0.33669 |

**References**

1 White, C. R., Phillips, N. F. & Seymour, R. S. The scaling and temperature dependence of vertebrate metabolism. *Biol Lett* **2**, 125-127, doi:10.1098/rsbl.2005.0378 (2006).

2 Makarieva, A. M. *et al.* Mean mass-specific metabolic rates are strikingly similar across life's major domains: Evidence for life's metabolic optimum. *Proceedings of the National Academy of Sciences of the United States of America* **105**, 16994-16999, doi:10.1073/pnas.0802148105 (2008).

3 Froese, R. a. D. P. E. FishBase, World Wide Web electronic publication. www.fishbase.org, version. (2012).

4 Clarke, A., Rothery, P. & Isaac, N. J. Scaling of basal metabolic rate with body mass and temperature in mammals. *J Anim Ecol* **79**, 610-619, doi:10.1111/j.1365-2656.2010.01672.x

JAE1672 [pii] (2010).
