# Supplementary material for: Effect of temperature and glia in brain size enlargement and origin of allometric body-brain size scaling in vertebrates
Source: BMC Evol Biol. 2014 Oct 3;14:178. doi: 10.1186/s12862-014-0178-z (PMC4193995; doi:10.1186/s12862-014-0178-z)
Supplement: Additional file 4: — Animal datasets for the allometric scaling relationship between brain mass and total neuronal number for endothermic and ectothermic animals. Data from 12 mammals (Table S4a), 11 primates (Table S4b) and 7 ectotherms (Table S4c). [file 12862_2014_178_MOESM4_ESM.docx]

**Supplement Table S4 Dataset:**

**Allometric scaling relationship between brain mass and total neuronal number for endothermic and ectothermic animals.** Data from 12 mammals (Table S4a), 11 primates (Table S4b) and 7 ectotherms (Table S4c).

**Table S4a: brain mass vs total neuron number for mammals**[**^1^**](#_ENREF_1)**^,^**[**^2^**](#_ENREF_2)

| **Number** | **Species** | **Brain mass (gram)** | **Neurons (million)** | **Non-neurons (million)** |
| --- | --- | --- | --- | --- |
| *1* | *Mouse* | 0.416 | 70.89 | 37.81 |
| *2* | *Hamster* | 1.02 | 89.97 | 76.15 |
| *3* | *Rat* | 1.802 | 200.13 | 131.52 |
| *4* | *Guinea pig* | 3.759 | 239.62 | 238.25 |
| *5* | *Agouti* | 18.365 | 856.74 | 1084.72 |
| *6* | *Capybara* | 76.036 | 1601.12 | 3265.32 |
| *7* | *Tree shrew* | 2.752 | 261.4 | 199.65 |
| *8* | *Smoky shrew* | 0.1893 | 39.49 | 25.62 |
| *9* | *Short-tailed shrew* | 0.375 | 58.83 | 37.42 |
| *10* | *Hairy-tailed mole* | 0.802 | 140.35 | 89.17 |
| *11* | *Star-nosed mole* | 0.845 | 141.87 | 90.51 |
| *12* | *Eastern mole* | 1.146 | 238.39 | 119.52 |

**Table S4a: brain mass vs total neuron number for primates**[**^3^**](#_ENREF_3)**^,^**[**^4^**](#_ENREF_4)

| **Number** | **Species** | **Brain mass (gram)** | **Neurons (million)** | **Non-neurons (million)** |
| --- | --- | --- | --- | --- |
| *1* | *Marmoset* | 7.780 | 635.80 | 590.74 |
| *2* | *Galago* | 10.150 | 936.00 | 666.59 |
| *3* | *Owl monkey* | 15.73 | 1468.41 | 1195.13 |
| *4* | *Squirrel monkey* | 30.216 | 3246.43 | 2075.03 |
| *5* | *Capuchin monkey* | 52.208 | 3690.52 | 3297.74 |
| *6* | *Macaque monkey* | 87.346 | 6376.16 | 7162.9 |
| *7* | *Microcebus murinus* | 1.799 | 254.71 | 138.95 |
| *8* | *Macaca fascicularis* | 46.16 | 3440 | 3150 |
| *9* | *Macaca radiata* | 61.47 | 3780 | 4880 |
| *10* | *Papio cynocephalus* | 151.19 | 10950 | 9180 |
| *11* | *Humans* | 1508 | 86000 | 85000 |

**Table S4c: brain mass vs total neuron number for ectotherms**

| **Number** | **Species** | **Brain mass (gram)** | **Neurons (million)** | **Non-neurons (million)** |
| --- | --- | --- | --- | --- |
| *1* | Frog Rana esculenta[^5^](#_ENREF_5) | 0.109 | 15.8 |  |
| *2* | Bull frog[^5^](#_ENREF_5) | 0.24 | 15.8 |  |
| *3* | Zebrafish[^6^](#_ENREF_6) | 0.2 | 10 |  |
| *4* | Apteronotus leptorhynchus[^7^](#_ENREF_7) | 0.118 | 62.8 |  |
| *5* | Apteronotus leptorhynchus[^7^](#_ENREF_7) | 0.06262 | 32 |  |
| *6* | Octopus vulgaris[^8^](#_ENREF_8) | 1.598 | 364 |  |
| *7* | Octopus vulgaris[^8^](#_ENREF_8) | 0.128 | 60.25 |  |
| *8* | Octopus vulgaris[^8^](#_ENREF_8) | 0.351 | 129 |  |

**References**

1 Sarko, D. K., Catania, K. C., Leitch, D. B., Kaas, J. H. & Herculano-Houzel, S. Cellular scaling rules of insectivore brains. *Frontiers in neuroanatomy* **3**, 8, doi:10.3389/neuro.05.008.2009 (2009).

2 Herculano-Houzel, S., Mota, B. & Lent, R. Cellular scaling rules for rodent brains. *Proceedings of the National Academy of Sciences of the United States of America* **103**, 12138-12143, doi:10.1073/pnas.0604911103 (2006).

3 Gabi, M. *et al.* Cellular scaling rules for the brains of an extended number of primate species. *Brain, behavior and evolution* **76**, 32-44, doi:10.1159/000319872 (2010).

4 Herculano-Houzel, S., Collins, C. E., Wong, P. & Kaas, J. H. Cellular scaling rules for primate brains. *Proceedings of the National Academy of Sciences of the United States of America* **104**, 3562-3567, doi:10.1073/pnas.0611396104 (2007).

5 Kemali, M. & Braitenberg, V. *Atlas of the frog's brain*. (Springer, 1969).

6 Froese, R. a. D. P. E. FishBase, World Wide Web electronic publication. www.fishbase.org, version. (2012).

7 Zupanc, G. K. & Horschke, I. Proliferation zones in the brain of adult gymnotiform fish: a quantitative mapping study. *The Journal of comparative neurology* **353**, 213-233, doi:10.1002/cne.903530205 (1995).

8 Packard, A. & Albergoni, V. Relative growth, nucleic acid content and cell numbers of the brain in Octopus vulgaris (Lamarck). *The Journal of experimental biology* **52**, 539-552 (1970).
