# Supplementary material for: Effect of temperature and glia in brain size enlargement and origin of allometric body-brain size scaling in vertebrates
Source: BMC Evol Biol. 2014 Oct 3;14:178. doi: 10.1186/s12862-014-0178-z (PMC4193995; doi:10.1186/s12862-014-0178-z)
Supplement: Additional file 5: — Animal datasets for the ratios of brain metabolism to resting body metabolism. Data from 26 endotherms (Table S5a) and 7 ectotherms (Table S5b). [file 12862_2014_178_MOESM5_ESM.docx]

**Supplement Table S5 Dataset:**

**Ratios of brain metabolism to resting body metabolism.** Data from 26 endotherms (Table S5a) and 7 ectotherms (Table S5b).

**Table S5a: brain mass, basal metabolic rate (BMR), total neuron number, individual neuronal metabolic cost, body mass and body BMR for endotherms**[**^1^**](#_ENREF_1)

| **Species** | **Body**  **Weight**  **(g)** | **Brain**  **Weight**  **(g)** | **Body**  **BMR**  **(watts)** | **Brain**  **BMR**  **(watts)** | **BMR**  **Ratios** | **Total**  **Neurons (million)** | **Energy cost of individual neurons (x 10^-10^ watts)** |
| --- | --- | --- | --- | --- | --- | --- | --- |
| Mouse | 16.5 | 0.425 | 0.1541 | 0.0114 | 0.07933 | 70.89 | 1.603 |
| Rat | 278 | 2.3 | 1.2111 | 0.0561 | 0.04763 | 200.13 | 2.88233 |
| Cat | 2673 | 27.56 | 6.666 | 0.396 | 0.0633 | 1000 | 4.09266 |
| Macaque | 3627 | 93.1 | 9.174 | 1.122 | 0.12415 | 7300 | 1.55719 |
| Baboon | 6619 | 147 | 14.619 | 1.8381 | 0.07657 | 10910 | 1.02711 |
| Squirrel | 878 | 5.63 | 2.607 | 0.1076 | 0.04125 | 472.44 | 2.27695 |
| Shrew | 5.3 | 0.11 | 0.2805 | 0.0036 | 0.01139 | 31 | 1.13584 |
| Chimpanzee | 24500 | 430.5 | 33.876 | 4.674 | 0.138 | 6700 | 6.976 |
| Human | 54333 | 1273 | 68.67 | 13.863 | 0.202 | 86000 | 1.612 |
| Balaenoptera physalus | 4.8E7 | 8100 | 11220 | 60.06 | 0.0054 |  |  |
| Elephant | 3.05E6 | 4717 | 2032.8 | 37.62 | 0.01851 |  |  |
| Cattle | 506000 | 420 | 290.4 | 4.587 | 0.0158 |  |  |
| Dolphin | 120000 | 1296 | 254.1 | 12.190 | 0.04797 |  |  |
| Sheep | 40230 | 109 | 49.17 | 1.416 | 0.0288 |  |  |
| Pig | 51500 | 100 | 72.6 | 1.313 | 0.01809 |  |  |
| Seal | 39680 | 255 | 109.89 | 2.963 | 0.02696 |  |  |
| Chicken | 1800 | 3.68 | 5.016 | 0.0743 | 0.01481 |  |  |
| Dog | 10784 | 78.07 | 21.252 | 0.875 | 0.04117 |  |  |
| Orangutan | 21000 | 316 | 33.33 | 3.564 | 0.10693 |  |  |
| Pigeon | 326 | 9.3 | 1.5609 | 0.0396 | 0.02537 |  |  |
| Fox | 3385 | 44.5 | 9.867 | 0.650 | 0.06588 |  |  |
| Marmot | 1980 | 13.2 | 4.7619 | 0.226 | 0.04746 |  |  |
| Guinea pig | 649.44 | 4 | 2.145 | 0.08 | 0.0373 |  |  |
| Sparrow | 27 | 1 | 0.528 | 0.0238 | 0.04508 |  |  |
| Camel | 453590 | 569.5 | 240.9 | 5.966 | 0.02477 |  |  |
| Horse | 548870 | 650 | 445.5 | 6.7 | 0.01504 |  |  |
| Bat | 28 | 0.936 | 0.3432 | 0.0226 | 0.06585 |  |  |
| Mole | 39.6 | 1.16 | 0.4752 | 0.0272 | 0.05724 |  |  |

**Table S5b: brain mass, basal metabolic rate (BMR), total neuron number, individual neuronal metabolic cost, body mass and body BMR for ectotherms**[**^1^**](#_ENREF_1)

| **Species** | **Body**  **Weight**  **(g)** | **Brain**  **Weight**  **(g)** | **Body**  **BMR**  **(watts)** | **Brain**  **BMR**  **(watts)** | **BMR**  **Ratios** | **Total**  **Neurons (million)** | **Energy cost of individual neurons (x 10^-11^ watts)** |
| --- | --- | --- | --- | --- | --- | --- | --- |
| Goldfish | 9.52 | 0.097 | 0.01386 | 8.91E-4 | 0.06429 | 15 | 5.94 |
| Toad | 44.5 | 0.073 | 0.01455 | 6.93E-4 | 0.04763 | 13 | 5.3308 |
| Frog | 45.86 | 0.097 | 0.02277 | 8.91E-4 | 0.03913 | 15.8 | 5.6392 |
| Bullfrog | 520 | 0.4625 | 0.11154 | 0.00353 | 0.03165 |  |  |
| Turtle | 849.8 | 0.727 | 0.1876 | 0.00521 | 0.02777 |  |  |
| Squalus acanthias | 4800 | 3.87 | 1.1121 | 0.02234 | 0.02009 |  |  |
| Alligator | 52400 | 7.23 | 2.0757 | 0.04026 | 0.0194 |  |  |

**References**

1 Mink, J. W., Blumenschine, R. J. & Adams, D. B. Ratio of central nervous system to body metabolism in vertebrates: its constancy and functional basis. *American Journal of Physiology - Regulatory, Integrative and Comparative Physiology* **241**, R203-R212 (1981).
